# Aging and Charge Compensation Effects of the Rechargeable Aqueous Zinc/Copper Hexacyanoferrate Battery Elucidated Using In Situ X-ray Techniques

**DOI:** 10.1021/acsami.1c19167

**Published:** 2021-12-08

**Authors:** Mikaela Görlin, Dickson O. Ojwang, Ming-Tao Lee, Viktor Renman, Cheuk-Wai Tai, Mario Valvo

**Affiliations:** † Department of Chemistry-Ångström Laboratory, Uppsala University, P.O. Box 538, SE-75121 Uppsala, Sweden; ‡ Department of Materials Science and Engineering, Norwegian University of Science and Technology, NO-7491 Trondheim, Norway; § Department of Materials and Environmental Chemistry, Stockholm University, SE-106 91 Stockholm, Sweden

**Keywords:** aqueous rechargeable zinc-ion batteries, zinc copper hexacyanoferrate, Prussian blue analogues, aging effects, charge compensation process, in situ X-ray absorption spectroscopy, X-ray photoelectron spectroscopy, X-ray diffraction

## Abstract

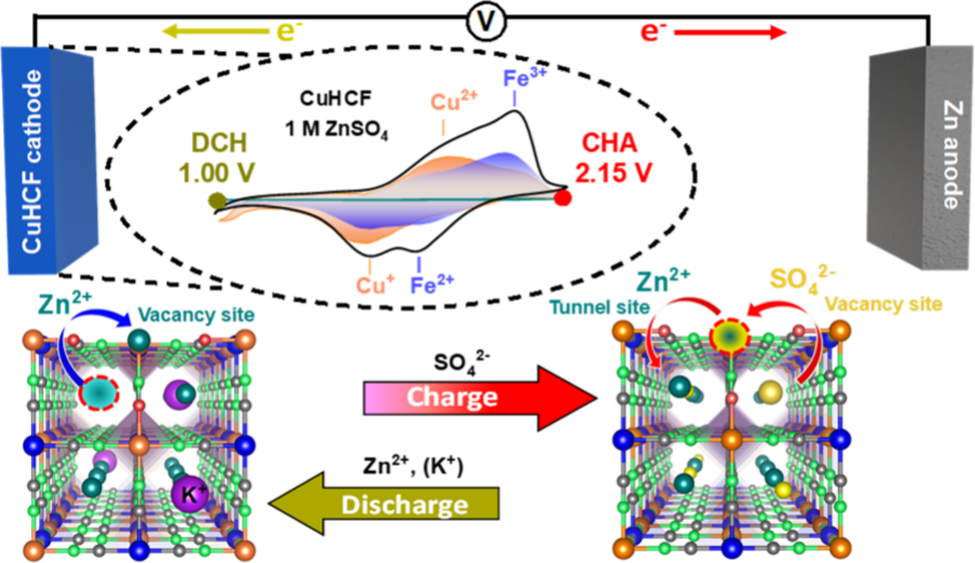

The zinc/copper hexacyanoferrate (Zn/CuHCF) cell has gained attention as an aqueous rechargeable zinc-ion battery (ZIB) owing to its open framework, excellent rate capability, and high safety. However, both the Zn anode and the CuHCF cathode show unavoidable signs of aging during cycling, though the underlying mechanisms have remained somewhat ambiguous. Here, we present an in-depth study of the CuHCF cathode by employing various X-ray spectroscopic techniques. This allows us to distinguish between structure-related aging effects and charge compensation processes associated with electroactive metal centers upon Zn^2+^ ion insertion/deinsertion. By combining high-angle annular dark-field-scanning electron transmission microscopy, X-ray absorption spectroscopy (XAS), X-ray photoelectron spectroscopy, and elemental analysis, we reconstruct the picture of both the bulk and the surface. First, we identify a set of previously debated X-ray diffraction peaks appearing at early stages of cycling (below 200 cycles) in CuHCF. Our data suggest that these peaks are unrelated to hypothetical Zn_*x*_Cu_1–*x*_HCF phases or to oxidic phases, but are caused by partial intercalation of ZnSO_4_ into graphitic carbon. We further conclude that Cu is the unstable species during aging, whose dissolution is significant at the surface of the CuHCF particles. This triggers Zn^2+^ ions to enter newly formed Cu vacancies, in addition to native Fe vacancies already present in the bulk, which causes a reduction of nearby metal sites. This is distinct from the charge compensation process where both the Cu^2+^/Cu^+^ and Fe^3+^/Fe^2+^ redox couples participate throughout the bulk. By tracking the K-edge fluorescence using operando XAS coupled with cyclic voltammetry, we successfully link the aging effect to the activation of the Fe^3+^/Fe^2+^ redox couple as a consequence of Cu dissolution. This explains the progressive increase in the voltage of the charge/discharge plateaus upon repeated cycling. We also find that SO_4_^2–^ anions reversibly insert into CuHCF during charge. Our work clarifies several intriguing structural and redox-mediated aging mechanisms in the CuHCF cathode and pinpoints parameters that correlate with the performance, which will hold importance for the development of future Prussian blue analogue-type cathodes for aqueous rechargeable ZIBs.

## 1 Introduction

The use of renewable energies is imperative to lower the greenhouse gas emissions and progressively develop a society free of fossil fuel dependence.^1^ Storage of electricity produced via renewable energies is a critical link toward greener energy production and distribution worldwide. Harnessing electricity generated by renewable sources requires the development of versatile and robust energy storage systems (ESSs). Attaining both low-cost, minimum environmental footprint, and high safety is important to facilitate the use of these systems on a larger scale.^2^ Rechargeable batteries are the fastest-growing EESs owing to their efficient use in electric vehicles (EVs), where Li-ion batteries (LIBs) currently is the dominating technology.^3−5^ However, less attention has been paid to “greener” EES options for stationary storage applications. Rechargeable aqueous zinc-ion batteries (ZIBs) are considered one of the most promising upcoming technologies for stationary applications such as grid storage and uninterruptible power supply applications due to their nontoxicity, low price, high safety (i.e., nonflammability), and abundance of the zinc metal.^6−11^

Despite the relatively low energy density and short cycle life compared to that of, for example, LIBs, the zinc metal has a high gravimetric capacity of 820 mAh g^–1^ and a low reduction potential (−0.78 V vs standard hydrogen electrode).^12^ This allows for high operational voltages within the electrochemical stability window of water.

Prussian blue analogues (PBAs) have recently gained interest as cathode materials due to their open framework, which allows for reversible Zn^2+^ ion insertion/deinsertion, easy synthesis routes, and compatibility with a variety of metal cations.^13−22^ These open framework compounds open up for the use of electrolytes with a mild pH, thus avoiding strongly acidic or alkaline conditions.

Here, we investigate the zinc/copper hexacyanoferrate (Zn/CuHCF) system, a PBA suggested as a possible ZIB cathode material in 2015 by Trócoli and La Mantia^18^ and Jia et al.,^17^ being known at the time to host other cations.^19,23−25^ CuHCF has a typical gravimetric capacity of around 60 mAh g^–1^, a high operating potential of ∼1.7 V, and negligible structural changes and volume expansion during Zn^2+^ ion insertion (i.e., near-zero strain). This allows for fast ion insertion and makes this type of ZIBs more suitable for high power applications compared to, for example, manganese dioxide batteries (Zn/MnO_2_), which exhibit generally higher gravimetric capacities instead.

PBAs are structural analogues with perovskites, where bridged cyanide ligands (CN) form a three-dimensional network in an octahedral arrangement around the central transition metal atom. The generic formula can be written as A_*x*_M[M′(CN)_6_]_1–*y*_·□_*y*_·*n*H_2_O (0 < *x* < 1, *y* < 1), where in CuHCF, A = Zn^2+^ or zeolitic water, M = Cu^2+^, Ḿ = Fe^3+^, and □ = Fe(CN)_6_ vacancy.^13,26^ The outer M site (Cu^2+^) is coordinated to the N-end, while the inner M′ site (Fe^3+^) is coordinated to the C-end. In our CuHCF material, one-third of the Fe(CN)_6_ vacancies in the native structure are attributed to the Cu/Fe ratio of ∼1.5, which typically are occupied by coordinating water molecules.^13,27^

Despite the relatively short cycle life and low energy density that comes with CuHCF and PBA-type cathodes in rechargeable aqueous systems, several advantageous features such as environmental compatibility, low cost, safety, moderate capacity, and fast ion insertion still motivate the use of these compounds in future ZIBs. The development of aqueous PBA-based ZIBs is still at its infant stage, and despite considerable advances in the recent past, there are various processes related to aging and capacity fade that are still not well-understood.^28−32^ This is what motivates this study.

Dissolution of metal species from CuHCF (especially Cu) has been observed during cycling, although the extent of this is still debated. Furthermore, Zn^2+^ ions have been reported to become irreversibly trapped in CuHCF, which is proposed to trigger phase segregation and formation of new nonstoichiometric Zn-rich phases (Zn_*x*_Cu_1–*x*_CuHCF).^28,29,32,33^ These phases are thought to be responsible for new X-ray diffraction (XRD) peaks that appear with cycling, although some of the peaks have not yet been conclusively identified.^28,30,34^ The Zn^2+^ trapping and formation of new Zn-rich phases have been proposed to be responsible for shifting the Zn^+^ ion insertion (or the charge/discharge voltage plateaus) to higher potentials.^29,32^ Nevertheless, uncertainties still remain around both the mechanism of Cu dissolution and the origin of these new diffraction peaks, especially at early stages of cycling.^11^ A new charge compensation mechanism based on operando XRD was earlier put forward by Renman et al.,^31^ where trapped Zn^2+^ ions were proposed to swap between interstitial tunnel sites (i.e., Wyckoff notation as 8c) and Fe(CN)_6_ vacancies (Wyckoff notation as 4a), thus explaining why Zn^2+^ ions do not exit CuHCF during charge.

During monovalent cation insertion, typically only one metal center is electroactive in PBAs (i.e., Fe^3+^/Fe^2+^); however, during divalent cation insertion (Zn^2+^), the second metal center may also become electroactive (i.e., Cu^2+^/Cu^+^).^16^ Currently, very few studies exist that metodically probe redox-active metal centers in the aqueous Zn/CuHCF cell. To the best of our knowledge, we are only aware of the investigation by Lim et al.,^30^ where electron energy loss spectroscopy revealed the participation of both Cu^2+^/Cu^+^ and Fe^3+^/Fe^2+^ redox couples during Zn^2+^ insertion. Otherwise, both electroactive Cu and Fe centers have been reported during Li^+^ ion insertion^35,36^ and K^+^ ion insertion in similar CuHCF materials.^37^

Here, we employ various in situ X-ray spectroscopic techniques to clarify the aging, charge compensation, and degradation processes in the aqueous Zn/CuHCF cell up to 200 cycles. We employ a combination of X-ray absorption spectroscopy (XAS), X-ray photoelectron spectroscopy (XPS), X-ray diffraction (XRD), aberration-corrected high-angle annular dark-field scanning transmission electron microscopy (HAADF-STEM), and elemental analysis. In short, we resolve the electroactivity related to both the Cu^2+^/Cu^+^ and Fe^3+^/Fe^2+^ redox couples during Zn^2+^ insertion, which we link to the redox peaks in the cyclic voltammetric profile in a potentiodynamic fashion. We further reveal new information on the extent and location of Cu dissolution, we relate previously debated XRD peaks to graphitic carbon, and we make new relevant observations of SO_4_^2–^ anions participating in the charge compensation process. Our findings and discussion provide an in-depth understanding of the limiting factors for this intriguing aqueous Zn/CuHCF system. The detailed analyses, combined with our tailored X-ray approach, pave the way toward the development of aimed methodologies for accurate insights into the key components of these ZIBs, thus enabling future improvements of this challenging rechargeable Zn-ion technology.

## 2 Results and Discussion

### 2.1 Electrochemical Behavior of the Aqueous Zn/CuHCF Cell

**Figure 1:**
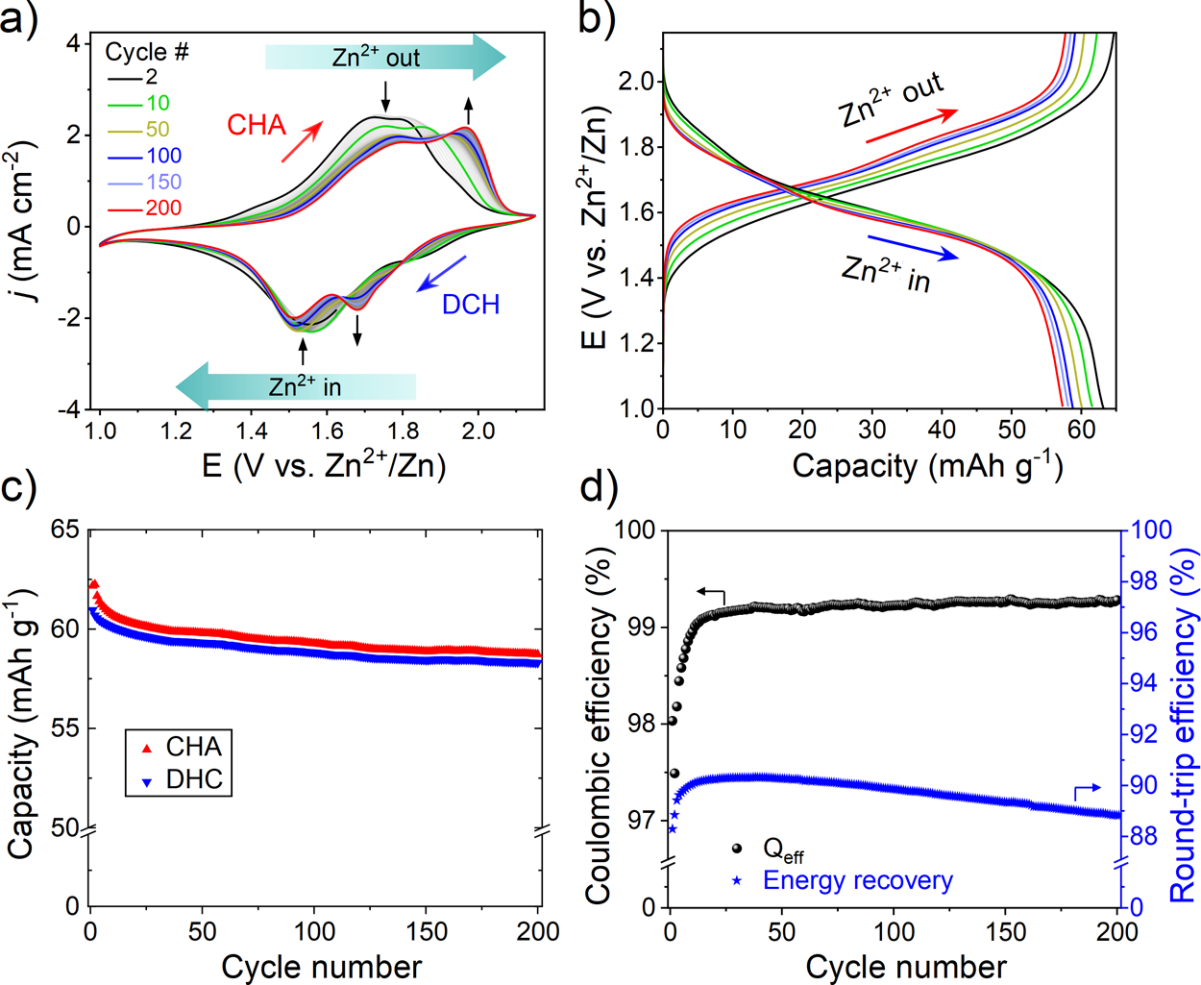
Voltammetric and galvanostatic charge/discharge profiles of the Zn/CuHCF cell in 1 M ZnSO_4_. (a) CV curves at 2.5 mV s^–1^ showing the first 200 cycles of the Zn/CuHCF cell. (b) Galvanostatic charge/discharge voltage profiles obtained at around 8C rate. (c) Evolution of the charge/discharge capacities in each cycle obtained from galvanostatic cycling at around 8C rate. (d) Coulombic efficiency (left) and round-trip efficiency (right). Note the gradual increase of the voltage profiles for the charge curves in (b) with the development of a feature resembling a two-step voltage plateau with increasing cycle number. The first cycle has been omitted for clarity. All experiments were performed between 1.00 and 2.15 V vs Zn^2+^/Zn. The red arrows in (a–b) indicate the charge process (CHA), and the blue arrows indicate the discharge process (DCH).

The electrochemical characteristics of the aqueous Zn/CuHCF cell are shown in Figure 1a,d. A depotassiated form of CuHCF was employed as the cathode (K_2*x*/3_Cu^2+^[Fe^3+^(CN)_6_]_2/3_·3.3H_2_O, *x* ≈ 0), and metallic Zn as the anode. The cathode is made up of 75 wt % CuHCF, 15 wt % polyvinyl alcohol (PVA) binder, and 10 wt % carbon black (CB). The experimental Cu/Fe ratio is ∼1.5 in the pristine CuHCF material, which was determined from inductively coupled plasma-optical emission spectroscopy (ICP-OES). This ratio is known to result in ∼1/3 of Fe(CN)_6_ vacancies in the native crystal structure. The water content was estimated using thermogravimetric analysis, which further allowed for the determination of the molecular weight (267.07 g mol^–1^) (see Supporting Information Figure S1).

Cyclic voltammetry (CV) of the Zn/CuHCF cell at a sweep rate of 2.5 mV s^–1^ (corresponding to ∼8C) in 1 M ZnSO_4_ reveals a typical double-peak feature during discharge/charge as Zn^2+^ ions insert/deinsert into the CuHCF framework (Figure 1a), in accordance with previous studies.^29,32^ On the anodic sweep (i.e., during charge/Zn^2+^ ion “deinsertion” or swapping to 8c interstitial tunnel sites), there are two redox features at ∼1.7 V and at ∼1.8 V versus Zn^2+^/Zn (Figure 1a). A weak prefeature is sometimes visible at ∼1.4 V; however, this usually disappears after only a few cycles. The first feature at 1.7 V has been attributed to the Cu^2+^/Cu^+^ redox couple and the second feature at 1.8 V to the Fe^3+^/Fe^2+^ redox couple.^38,39^ Both these peaks progressively shift to higher average potentials with cycling, although the first feature decreases in size significantly, whereas the second feature instead increases with cycling while shifting to higher potential (i.e., from 1.8 V to 1.9–2.0 V), see Figure 1a. The same set of peaks is present on the cathodic sweep (i.e., during discharge/Zn^2+^ “insertion” or swapping to 4a vacancy sites), although the peaks shift to slightly lower average potentials with cycling, instead. There are still uncertainties regarding these double features and the underlying mechanism that causes them to change with cycling. It was speculated early on whether these are influenced by electrostatic repulsion from the insertion of the divalent Zn^2+^ ion^18^ and more recently by the evolution of new Zn-rich phases (Zn_x_Cu_1-x_HCF) due to the irreversible trapping of Zn^2+^ ions in Fe(CN)_6_ vacancies of CuHCF.^28,29,32,33^ A thorough investigation of these peaks and how they correlate with the electroactivity of Cu and Fe sites will be presented in this study.

The galvanostatic voltage profiles at ∼8C cycling rate are shown in Figure 1b. Based on the loadings of the active material determined from ICP-OES, the gravimetric capacity of our CuHCF material is on average 64 ± 10 mAh g^–1^ at 8C, which is in line with the proposed theoretical capacity of this material.^18^ We note that at slower rates, more capacity can be accessed, and at 1C, the cell delivers a practical charge/discharge capacity of 81 ± 9 mAh g^–1^ (Figure S2a,b). This is slightly higher than the initially proposed capacity; however, our calculations support these as the expected capacities based on the differences in the electron transfer number at different cycling rates (Figure S2c,d and eqs S1–S5). It is also in line with the study by Jiang et al.,^26^ who reported a theoretical capacity of 87 mAh g^–1^, and with Naveed et al.,^40^ who reported a capacity of 73 mAh g^–1^ for their Zn/CuHCF cells. It should be kept in mind that the capacity depends on several parameters, such as the number of water molecules in the structure (related to the molecular weight), the electron transfer number (i.e., *n*, the number of e^–^ transferred per metal site), and the loading of the active material. These are often challenging to determine precisely by experiments, since they may vary during the electrochemical reaction. Thus, the delivered practical charge/discharge capacities may not always perfectly agree with the theoretical or expected capacities. The operational voltage of our Zn/CuHCF cell is initially ∼1.65 V, with an average Coulombic efficiency of ∼99.2% over 200 cycles and a round-trip efficiency of ∼89.7% (Figure 1d and eq S6). The capacity nevertheless fades with cycling, and ∼15–20% of the initial capacity is lost after 200 cycles at 8C (ca. 56 h of continuous cycling). This is linked to the appearance of a sloping two-phase plateau, which is often reported as a characteristic aging effect in CuHCF (Figure 1b,c).^31^ The charging plateau shifts to higher average potentials compared to the discharge curve (Figures 1b and S2e). This highlights an asymmetry between the charge/discharge processes that evolves with cycling, which is likely related to differences between the anode Zn stripping/plating or the cathode Zn deinsertion/insertion processes. Electrochemical impedance spectroscopy confirmed an increase in the cell resistance (*R*_u_, iR-drop) by ∼60% from ∼5 to ∼8 Ω after 200 cycles (Figure S2f). Recovery tests by introducing an aimed pause of 24 h after completing 200 cycles show that the initial capacity cannot be recovered by this protocol and should therefore be regarded as an irreversible charge loss (Figure S3). Contributions from other processes, such as the stability of CuHCF and the redox activity of Cu and Fe centers to this aging behavior, will be investigated in detail in the sections below.

### 2.2 Structure-Related Aging Effects of CuHCF

**Figure 2:**
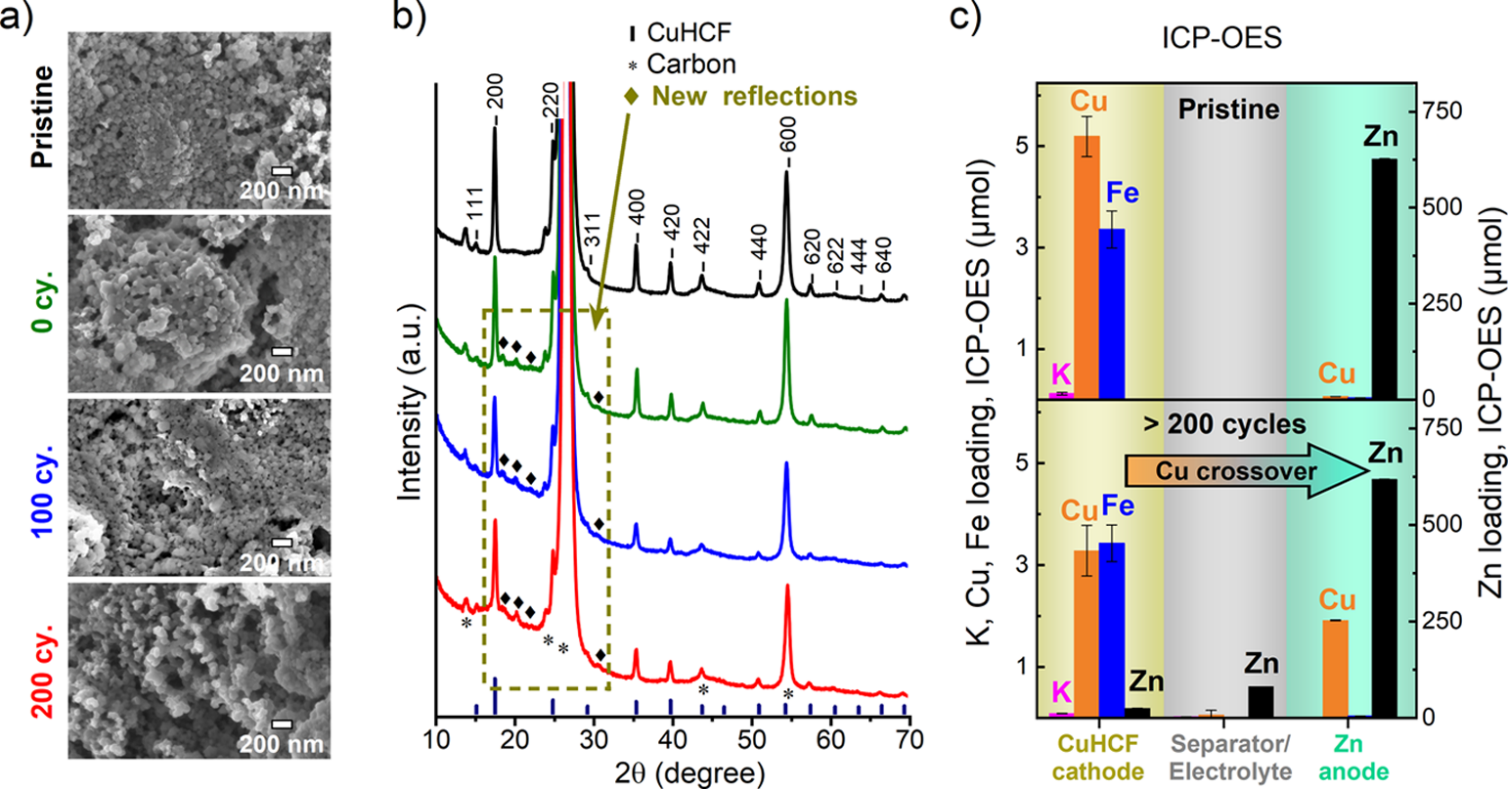
Aging of the aqueous Zn/CuHCF cell after cycling in 1 M ZnSO_4_ between 1.00 and 2.15 V at ∼8C and stopping the cells at OCP. (a) SEM micrographs of the CuHCF cathode; pristine (black), 0 cycles (i.e., resting 1 h at OCP, green), after 100 cycles (blue), and after 200 cycles (red). (b) Corresponding XRD patterns of the CuHCF cathode between 10 and 70° in the 2θ angular range; the color code in (a) also applies here. (c) Elemental analysis of the Zn/CuHCF cell parts using ICP-OES (cathode, separator/electrolyte, and anode). Top: the pristine state. Bottom: after 200 CV cycles. The bars represent the metal loadings of K, Cu, Fe, and Zn, where the cathode area is shaded in yellow (left), the separator is shaded in gray (middle), and the Zn anode is shaded in light green (right). Note that all samples were rinsed with ultrapure water prior to the analysis to remove excess ZnSO_4_ except in the case of (c), where we intended to preserve ionic species.

Scanning electron microscopy (SEM) shows that CuHCF is composed of particles with an average size of ∼50 nm (Figures 2a and S4). The XRD pattern can be indexed with a cubic unit cell (Fm3̅m), in accordance with a previous study by Renman et al. of this material (Figure 2b).^31^ The CB and graphite foil, used as conductive additive and current collector, respectively, also give rise to additional background diffraction peaks, marked with asterisks in Figure 2b. The XRD pattern of the pure CuHCF powder without the additions of CB or PVA binder is shown in Figure S5a, where it is compared to the patterns of the bare graphite foil and the CB-PVA cast without active material. Upon electrochemical cycling in 1 M ZnSO_4_, there are no obvious morphological changes of the CuHCF cathode. However, there is a set of new diffraction peaks appearing at 18.5, 20.2, 22.0, and 30.5° (Figures 2b and S5b). These actually become visible already after 0 cycles, which corresponds to resting the cell for 1 h at open-circuit potential (OCP), although they progressively intensify with cycling. In previous studies, new diffraction peaks were reported in similar CuHCF cathodes, which are currently thought to be related to these new ZnHCF (or mixed Zn_*x*_Cu_1–*x*_HCF) phases caused by the irreversible trapping of Zn^2+^ ions inside the framework.^30,32,34^ These peaks usually appear at later stages of cycling (typically after ∼250–500 cycles), along with clear morphological changes, where both wires and cubes have been reported. Here, we do not observe any changes in the morphology, and the new diffraction peaks that appear at relatively early stages of cycling do not match with the proposed Zn_*x*_Cu_1–*x*_HCF phases. We note that Lim et al.^30^ also reported changes in their patterns after 0 cycles, directly after immersing the CuHCF cathode in the electrolyte. These peaks could also not be conclusively identified. In the cathode of Zn/MnO_2_ cells (in the presence of electrolyte Mn^2+^ ions), Chamoun et al.^41^ reported new diffraction peaks associated with a layered Zn-buserite phase during charge, and a zinc sulfate hydroxide phase during discharge. In Mn^2+^-free electrolytes, they observed some degree of Zn^2+^ trapping in the cathode similar to that in CuHCF, although hydroxide formation from H^+^ insertion was suppressed. It is therefore important to verify phase transformations and possible contributions from oxidic phases also in our CuHCF cathode.

Elemental compositions from energy-dispersive X-ray spectroscopy (EDS) show negligible changes in the relative oxygen content of the CuHCF cathode. This is in contrast to the Zn anode where the O content increases by ∼60 at. % after 200 cycles (Figure S6). EDS also confirms a relative loss of Cu that amounts to ∼30 at. % after 200 cycles, seen as a decrease in the Cu/Fe ratio from ∼1.5 to ∼1.0 (see Table S1). The loss of Cu is verified by ICP-OES, where the complete cell was analyzed part by part (cathode, separator/electrolyte, and anode). This confirms the loss of the original Cu content of CuHCF by ∼30 at. % after 200 cycles, and shows that Cu crosses over to the Zn anode (Figure 2c and Table S2). ICP-OES further verifies that the Cu content increases progressively with cycling on the Zn anode (Figure S7). We do not observe any loss of Fe, which confirms that Cu is the unstable species in CuHCF, in accordance with previous studies.^28−32^

In the Zn anode, SEM images reveal a platelet-like structure that appears in correlation with the emergence of diffraction peaks that match with a zinc sulfate hydroxide hydrate phase [3Zn(OH)_2_·ZnSO_4_·4H_2_O] (Figure S8).^42^ This phase will be denoted shortly as “Zn(OH)_2_”. A ZnO phase was reported on the Zn anode by Trócoli and La Mantia,^18^ which is different from the phase we detect here. We further observe small Cu-rich nanoparticles that decorate the Zn anode, which verifies that Cu crosses over (Figures S9 and S10). These particles are visible already after 0 cycles; however, they grow larger with cycling. The subsequent crossover and nucletion of Cu ions on the Zn anode after dissolution from CuHCF may explain why significant amounts of Cu impurities are rarely detected in the electrolyte.

What puzzles us is the significant loss of Cu from CuHFC already after 0 cycles (i.e., after resting the cell for ∼1 h at the OCP). This motivated us to investigate the spontaneity of this dissolution process closer. Using ICP-OES, we compared cycled cells with cells left resting at OCP for the same amount of time it takes to complete 0, 100, and 200 charge/discharge cycles (ca. 1 h, 27 h, 56 h). The results show that equal amounts of Cu are lost both after resting and after cycling the cells (Figure S11). The loss of Cu after ∼56 h at OCP amounts to 21 ± 4 wt % and after 200 cycles it amounts to 26 ± 11 wt %. Notably, despite similar losses of the active material, the capacity retention is significantly higher after resting the cell at OCP (3.5 ± 0.2%) compared to cycling (18 ± 4%) (see Figure S12). This advocates that there are other detrimental processes resulting in performance loss during cycling, which will have to be investigated in future studies. Our results conclude that the Cu dissolution from CuHCF is indeed spontaneous. Since time appears to be the prime factor determining the aging, faster cycling rates could result in a relatively higher charge retention since it takes a shorter time to complete a cycle. This may result in inconsitencies across studies depending on the cycling protocol.

Lastly, we cross-checked the stability in 0.1, 1, and 2 M ZnSO_4_, since the concentration has been demonstrated to be an important stability parameter in CuHCF.^32^ We find the lowest stability in 2 M; however, there is no difference between 0.1 and 1 M ZnSO_4_ (Figure S13a,b). This is in contrast to earlier studies where lower concentrations were correlated with higher charge retention.^32^ Instead, we observe a significant increase in the cell resistance in 0.1 M ZnSO_4_, which leads to distortions of the redox peaks and unfavorable Zn^2+^ insertion (Figure S13c-f). Most cells cycled with 0.1 M ZnSO_4_ fail even before completing 100 cycles. We therefore conclude that 1 M ZnSO_4_ is the optimal condition for our system.

### 2.3 STEM and EDS Mapping

**Figure 3:**
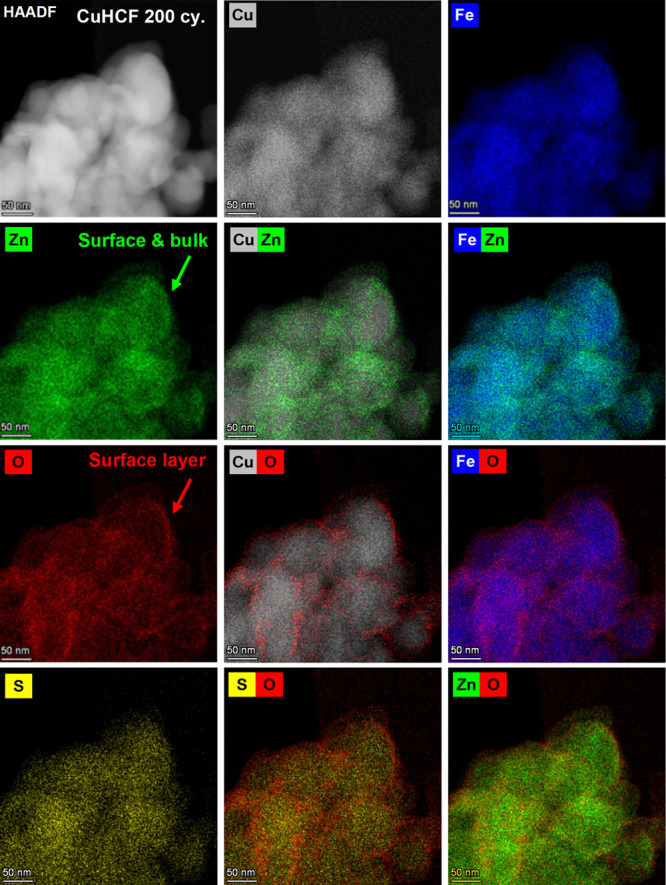
HAADF-STEM and EDS elemental maps of the CuHCF cathode after 200 CV cycles between 1.00 and 2.15 V in 1 M ZnSO_4_. The EDS maps of elements belonging to the CuHCF material are shown in the top row (Cu and Fe) and elements that appear after cycling in the left column (Zn, O, and S). Selected composite maps are labeled with the respective elements. The analyzed particles had been rinsed with ultrapure water prior to the analysis to remove excess ZnSO_4_ electrolyte. The scale bar is 50 nm.

To resolve the CuHCF particles better, we performed HAADF-STEM and EDS elemental mapping (Figure 3). In the pristine CuHCF cathode (containing PVA binder and CB conductive additive), there is a weak signal of K (likely impurities from the synthesis) and signals of N, C, Fe, and Cu belonging to CuHCF (Figure S14). There is also a weak signal of O distributed in the bulk of the pristine CuHCF particles, which could originate from the water in the structure, or possibly from the PVA or CB. Note that the C signal could also originate from PVA or CB, and there is a substantial Cu signal from the TEM grid. There are no signals from either Zn or S in the pristine sample. After 200 cycles in 1 M ZnSO_4_, the signal of K disappears, and new signals of Zn and S appear (Figure S15). Also, a thin layer of O enrichment is clearly notable on the outermost surface of the cycled particles, which was not visible in the pristine CuHCF particles (Figure 3). This O signal is nevertheless relatively weak, requires long acquisition times to be resolved, and is also somewhat segregated from the Zn (and Cu, Fe) signals. This suggests that these elements do not belong to the same phase. Therefore, we suspect that the surface O enrichment is related to other species than oxidic phases, which will be further investigated in Section 2.5.1 below. The elemental compositions from HAADF-STEM analysis are provided in Table S3.

### 2.4 Identification of XRD Peaks at the Early Stages of Cycling

**Figure 4:**
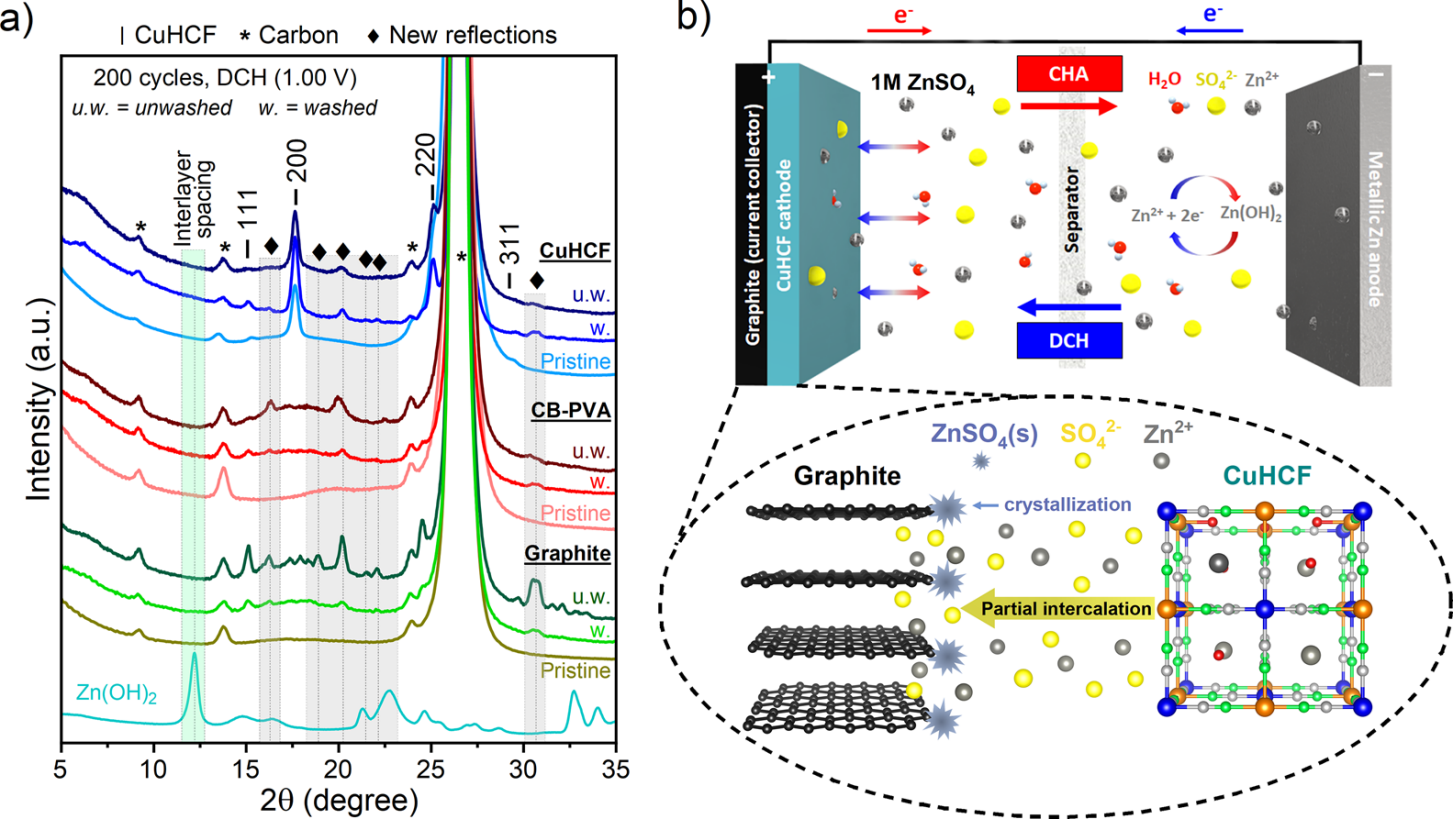
Identification of the XRD patterns of the Zn/CuHCF cell. (a) XRD patterns of cathodes: CuHCF and the two carbon “blank” cells (Zn/CB-PVA and Zn/graphite) after 200 cycles in 1 M ZnSO_4_ and stopped in the discharged state (DCH, 1.00 V). The washed (w.) and unwashed (u.w.) samples are shown for comparison. The charged state (CHA, 2.15 V) is shown in Figure S17. (b) Schematic representation summarizing the findings from the XRD and Raman spectroscopy analyses, demonstrating that Zn^2+^/SO_4_^2–^ ions from the electrolyte partially intercalate into graphitic carbon domains and/or crystallize close to the surface regions. The graphics in (b) is highly schematic and does not consider the realistic picture of the solvation shells around the electrolyte ions.

In search for a more solid explanation for the yet unidentified diffraction peaks that appear during the early stages of cycling in the CuHCF cathode, we investigated two control cells in which the active material was intentionally left out from the electrode coating. The idea was to examine whether these peaks could be related to other constituents of the cathode, such as graphitic carbon, which is often used as a conductive additive and can intercalate ions.^43,44^ The two control cells (denoted as “blank” cells) consisted of either CB-PVA (i.e., Zn/CB-PVA) or bare graphite foil (i.e., Zn/graphite). Interestingly, a set of nearly identical diffraction peaks to those seen in the CuHCF cathode also appear in these carbon blank cells after 200 cycles (Figure 4a). In fact, these peaks also emerge already after 0 cycles in these cells (i.e., after 1 h at OCP), although cycling promotes the peaks especially in the bare graphite cathode (Figure S16). The fact that the peaks are stronger in the graphite cathode compared to the CB-PVA cathode suggests that the new diffraction peaks are related to an intercalation process of ZnSO_4_ (Zn^2+^ and/or SO_4_^2–^) in graphitic carbon. The peaks are also more pronounced in the unwashed (u.w.) samples compared to the washed samples, and decrease significantly after the washing step, which indicates that the alleged character of the intercalating species is ionic. The blank cells stopped in the charged state (2.15 V) exhibit stronger diffraction peaks than the cells stopped in the discharged state (1.00 V) (Figures 4a and S17), which indicates that the intercalation process may be initiated during charge. However, since CuHCF modifies the cell potential, the intercalation process of ZnSO_4_ into carbon may be different in the presence/absence of CuHCF. To judge from the voltammetric profiles of the two carbon blank cells, there is no evidence of any major intercalation processes in the bulk (Figure S18).

Raman spectroscopy confirms a slight increase in the intensity of the characteristic D-band around 1350 cm^–1^ and the appearance/broadening of the D′-feature (∼1620 cm^–1^) in the cycled graphite cathodes (Figure S19). This signifies an increase in the local disorder in graphite, which is consistent with an intercalating species.^45^ There is no difference between the discharged (1.0 V) and charged (2.15 V) states; however, the D′ feature vanishes after washing the graphite electrodes with water, which supports the proposed ionic character. Furthermore, since the degree of disorder of graphite seen in the Raman spectra is relatively small, it supports that the intercalation process is restricted mainly to the surface regions. In the SEM images, there are small particles clearly visible on the surface of the graphite cathode after cycling, which show signals from Zn, S, and O (Figures S20 and S21 and Table S4). These particles can be removed to a large extent by rinsing the graphite electrode with water, although not completely, which also matches the behavior of the new XRD peaks. The combined picture suggests that these diffraction peaks appearing at early stages of cycling originate from crystallized ZnSO_4_ at the graphite electrode/electrolyte interface, where a small fraction intercalates into graphitic carbon domains in the surface regions (see the schematic illustration in Figure 4b).

### 2.5 Discerning between Surface and Bulk Aging Effects

To probe the electroactive metal centers both at the surface and in the bulk of the cathode during different stages of cycling (pristine and 0, 100, and 200 cycles), we employed a combination of XPS and XAS. These were performed in an “ex situ” configuration, meaning that the cells were disassembled after cycling and prior to the measurements. The cells investigated in this section were stopped at OCP and wahsed with ultrapure water prior to analysis. Information of the charged/discharged states is presented in Section 2.6 below.

#### 2.5.1 Surface Monitored by XPS

**Figure 5:**
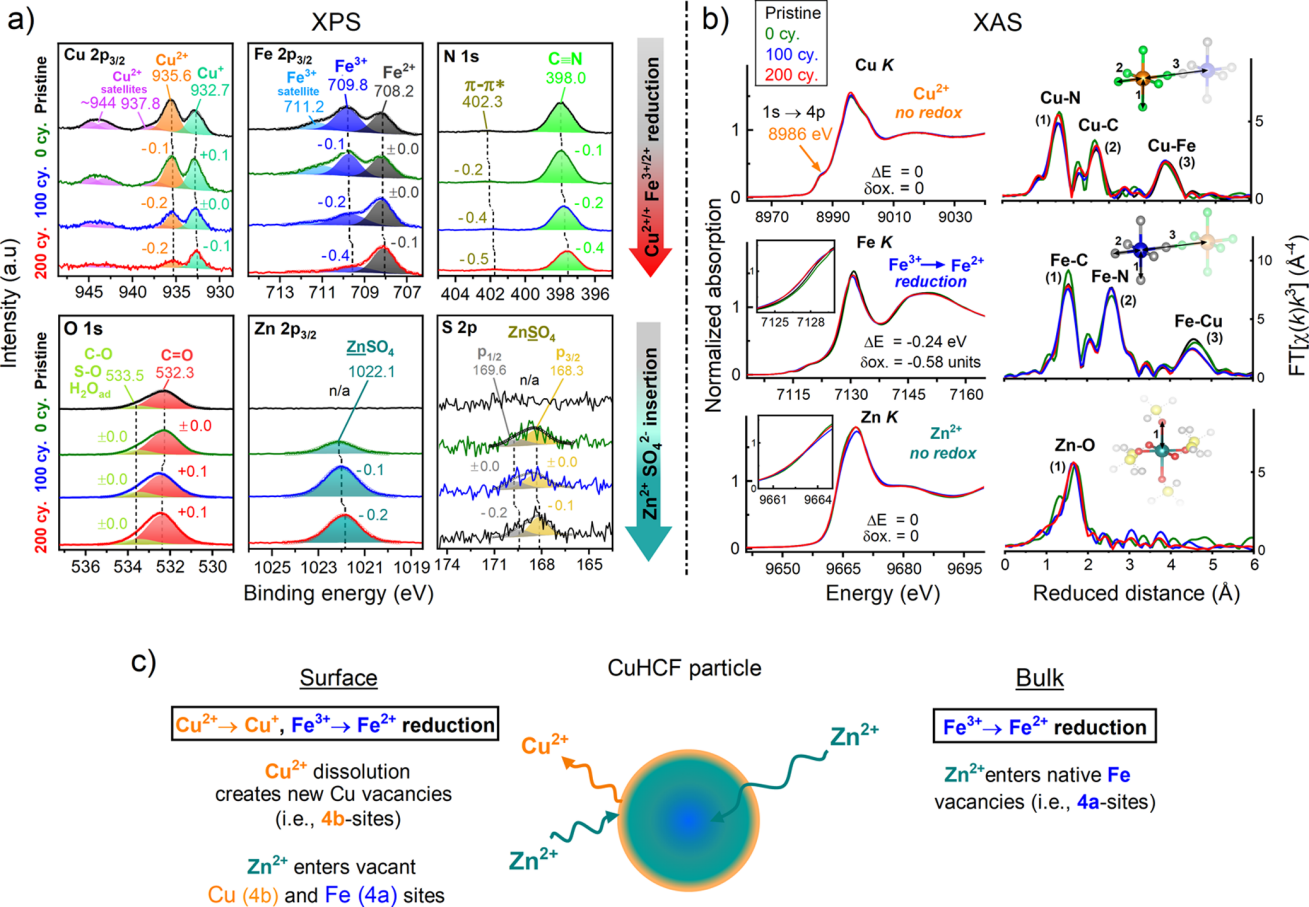
Aging of the CuHCF cathode monitored by XPS and XAS. The Zn/CuHCF cell was subjected to CV cycling between 1.00 and 2.15 V vs Zn^2+^/Zn at 2.5 mV s^–1^ in 1 M ZnSO_4_. (a) XPS after different stages of cycling; pristine, 0 cycles, 100 cycles, and 200 cycles. Top: Cu 2p_3/2_, Fe 2p_3/2_, and N 1s. Bottom: O 1s, Zn 2p_3/2_, and S 2p. The ± signs denote the relative shift in the B.E. with respect to that in the pristine state. (b) XANES region at the Cu, Fe, and Zn K-edges (left panel) and the corresponding FT-EXAFS spectra (right panel). (c) Schematic figures visualizing the findings for the surface and bulk. Note that Zn was not detected in the pristine CuHCF cathode. All measurements were stopped at the OCP, and the electrodes were rinsed with ultrapure water prior to the analysis to remove excess ionic species from the ZnSO_4_ electrolyte.

The XPS spectra were calibrated to the graphitic carbon peak (sp^2^-C) at a binding energy (B.E.) of 284.3 eV (Figure 5).^46^ In the C 1s spectrum, the peak at 285 eV accounts for either adventitious carbon, C–H, or C≡N (further denoted just as “CN”),^47−52^ the peak at ∼286 eV is assigned to C–O, and the peak at ∼289 eV to C=O (Figure S22).^46−48^ These may originate from either CB or the PVA binder. The Cu 2p_3/2_ and Fe 2p_3/2_ spectra are consistent with Cu^2+^ and Fe^3+^ ions in the pristine CuHCF material. With cycling, these are gradually reduced to Cu^+^ and Fe^2+^. After 200 cycles, the Cu^+^ peak at 932.7 eV has gained intensity, and the Cu^2+^ peak at 935.6 eV including the Cu^2+^ satellite peaks at ∼944/938 eV, have disappeared almost completely. Similarly, the Fe^2+^ peak at 708.2 eV has gained intensity, and the Fe^3+^ peak at 709.8 eV including the satellite peak at 711.2 eV associated with the unpaired electron in low-spin Fe^3+^, have disappeared almost completely. This suggests a near complete reduction to Cu^+^ and Fe^2+^ centers in the surface regions after 200 cycles. The XPS composition confirms the relative loss of both Cu and Fe, though a more significant loss of Cu, with an associated change in the Cu/Fe ratio from 1.4 to 0.2 after 200 cycles (Table S5). This is a more drastic loss of Cu compared to what we observed for the bulk, which suggests a more significant loss of Cu at the surface of the CuHCF particles.

In the O 1s spectrum, the peak at a B.E. of ∼532 eV originates from C=O, hydrated species such as absorbed H_2_O (zeolitic or coordinating) in CuHCF, or organic OH species from the PVA binder.^48,53,54^ We allocate the peak at ∼533 eV to C–O or SO species from ZnSO_4_;^48,49,55,56^ however, we could not distinguish between these species within the two envelopes. After 200 cycles, we do not observe any major changes in the O 1s spectrum, except for an increase in the overall peak area, which is confirmed as an increase in the relative O content by ∼5% (Table S5).

Most importantly, there is no shift in the B.E. of the O 1s peaks, or any peaks appearing in the oxide region, which suggests that the nature of these surface O species (also visible in the HAADF-STEM and EDS mapping) are not oxidic. Since there is a similar increase in the C–O component in the C 1s spectrum (Figure S22), these surface O species may be related to this.

The N 1s peak at a B.E. of 298 eV is assigned to the CN ligand,^57^ which shifts progressively by 0.4 eV to a lower B.E. after 200 cycles. This suggests a change in the electron density around the CN ligand,^58^ where a shift to a lower B.E. indicates the weakening of the CN bond. This can be explained by a higher degree of π-backdonation from the increased electron density around the reduced Cu^+^/Fe^2+^ sites into the π* orbital of the CN bond.^58,59^ In line with this discussion, there is a similar shift of both the Cu and Fe 2p peaks to a lower B.E. by ∼0.3 eV after 200 cycles, which could reflect a change in the electron density, as well. The Zn 2p_3/2_ peak at 1021 eV is assigned to Zn^2+^ ions in ZnSO_4_,^60^ and increases progressively with cycling, in accord with the proposed mechanism of irreversible trapping of Zn^2+^ ions in CuHCF.^31^ This correlates well with the changes in both the Cu and Fe spectra and suggests that Zn^2+^ ions enter vacant Cu and Fe sites, inducing a reduction of these sites. The S 2p peak at 168–170 eV is assigned to S species in ZnSO_4_,^60,61^ and a signal appears after 0 cycles (i.e., after 1 h at OCP) and then increases only moderately with cycling. The initial traces of K^+^ (∼0.1%) from the synthesis also disappear in the cycled samples (Figure S22).

For the sake of completeness, we further investigated the spontaneity of the aging process by XPS, whereby we left the Zn/CuHCF cells resting at OCP for the same amount of time that it takes to cycle 0, 100, and 200 cycles. Indeed, nearly identical changes occur in these CuHCF samples not exposed to cycling (Figure S23 and Table S6), which confirms that the aging process at the surface indeed is also spontaneous. XPS spectra of the relevant reference compounds in this context are provided in Figure S24.

#### 2.5.2 Bulk Monitored by XAS

The redox activity in the bulk of CuHCF was probed by XAS at the Cu, Fe, and Zn K-edges. The X-ray absorption near-edge structure (XANES) of the pristine CuHCF cathode is shown in Figure 5b (left panel) and exhibits a typical signature of metal hexacyanoferrates composed of linear Fe–CN–Cu units with the metal centers in an octahedral coordination.^27,36^ The Fe atom is connected to the C-end of the cyanide ligand and resides in a low-spin state, whereas the Cu atom is connected to the N-end and resides in a high-spin state.

The Cu XANES of the pristine cathode has a shoulder at ∼8986 eV (i.e., the dipole-allowed 1s → 4p transition) and a weak prefeature at ∼8977 eV (i.e., the quadruple-allowed 1s → 3d transition), which is a signature of Cu^2+^ in the d^9^ state with one unpaired electron (Figure 5b, top left, and Table S7).^36,62^ The Fe oxidation state is more straightforward and can be obtained from the edge position at half-height. We primarily find Fe^3+^ in the pristine sample (Figure 5b, middle left). The Zn K-edge is largely insensitive to the oxidation state;^63^ however, based on the spectral shape around the XANES region, we confirm dominating Zn^2+^ ions in an octahedral coordination environment (Figure 5b, bottom left, and Table S7).^53,64^ After cycling (up to 200 cycles), there are no visible changes in the Cu spectra; however, in the Fe spectra, the edge position progressively shifts by 0.4 eV to lower energies. Low-spin Fe shifts less than high-spin Fe, and the expected edge shift is ∼0.65 eV per oxidation state (see reference compounds in Figure S25 and Table S8). Our data is consistent with ∼60% of the Fe centers being reduced from Fe^3+^ → Fe^2+^ after 200 cycles. The Cu K-edge shows no change in the XANES region, with predominant Cu^2+^ species both in the pristine state and after 200 cycles. This suggests that the majority of the Cu species in the bulk are not redox-active. This is surprising since the XPS data clearly shows an almost complete reduction of both Fe and Cu sites at the surface. Our combined XAS and XPS data therefore suggest that the Cu^2+^ → Cu^+^ reduction happens mainly at the surface of the CuHCF particles, and is therefore likely related to the vacancies created upon Cu dissolution. There are no changes around the Zn K-edge in the cycled electrodes, whereby we conclude that Zn^2+^ ions are the predominant species without any changes upon cycling.

The local atomic structure parameters were obtained from simulations of the *k*^3^-weighed extended X-ray absorption fine structure (EXAFS) using scattering functions generated in FEFF. The Fourier transform EXAFS (FT-EXAFS) spectra are shown in Figure 5b (right panel) and Figure S26. The approach was adopted from previous studies^27,65,66^ and is described in detail in the Experimental Section in the Supporting Information. The FT-EXAFS spectra reveal three dominating peaks in CuHCF, where the Cu K-edge exhibits a Cu–N shell at ∼1.95 Å, a Cu–C shell at ∼3.11 Å, and a Cu–Fe shell at ∼4.9 Å. The Zn K-edge, which appears first after 0 cycles after resting the cell for 1 h at OCP, exhibits only a Zn–O scattering shell at ∼2.08 Å, which can be simulated with ∼6 oxygen ligands (see fit parameters in Tables S9 and S10). There are no major changes around this shell with cycling. Since this type of PBAs practically behaves as zero-strain materials, very little volume or structural changes are expected upon cation deinsertion/insertion and subsequent metal oxidation/reduction. Indeed, the variations in the EXAFS domains are too small to be detected herein. Our combined XPS and XAS data confirm no changes around the Zn K-edge XANES region, which also excludes the formation of new Zn_*x*_Cu_1–*x*_HCF phases up to 200 cycles, which have been reported between 250 and 1000 cycles in previous studies.^30,32,34^ Such phases may hence form at later stages of cycling, which will be addressed in future work. Our data further conclude that the Fe^3+^/Fe^2+^ redox couple is active in the entire bulk, while the Cu^2+^/Cu^+^ redox couple mainly is restricted to the surface of the CuHCF particles. This is consistent with Zn^2+^ trapping both in vacant Fe sites (Wyckoff notation as 4a) throughout the bulk, and in vacant Cu sites (Wyckoff notation as 4b) at the surface, the latter of which is triggered by Cu dissolution (see the schematic illustration in Figure 5c).

### 2.6 Probing Electroactive Sites Using In Situ XAS

**Figure 6:**
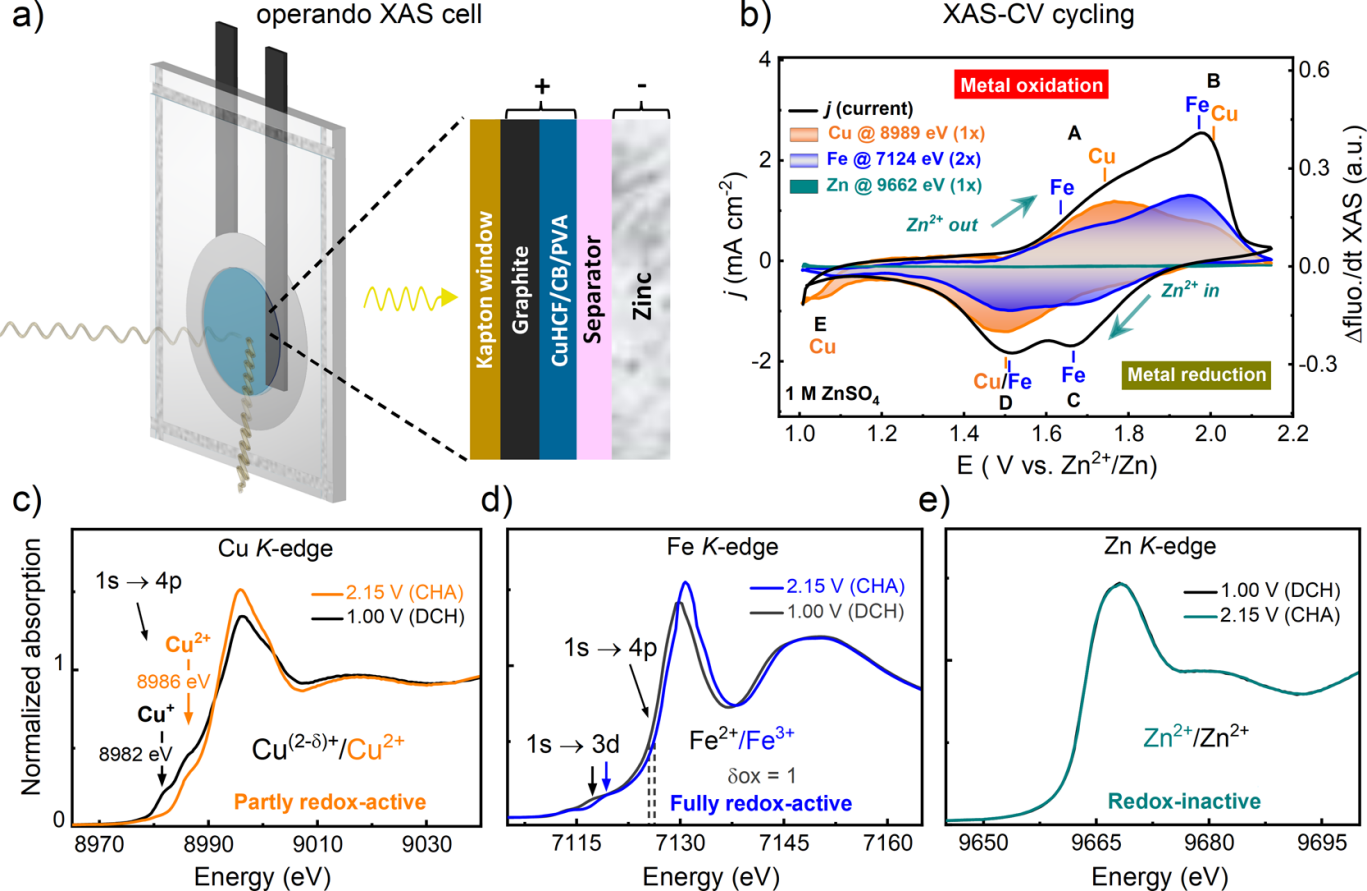
In situ XAS of the CuHCF cathode. (a) Schematic view of the XAS cell setup, consisting of a pouch cell with an X-ray transparent Kapton window. The fluorescence was monitored from the backside through the graphite foil. (b) Potentiodynamic XAS–CV cycling experiments carried out in the “operando” mode, where the fluorescence is monitored at the Cu, Fe, and Zn K-edges during cyclic voltammetry between 1.00 and 2.15 V in 1 M ZnSO_4_. The current density (*j*) is shown as a black curve, and the shaded areas correspond to the first derivative of the fluorescence, derived from the Cu K-edge (orange), Fe K-edge (blue), and Zn K-edge (dark cyan). The positive shaded areas represent an oxidation process, and the negative areas represent a reduction process. The Fe fluorescence has been scaled up 2 times (2×) for comparison reasons; however, the axis does not scale with the absolute oxidation states. The XANES region in the discharged (1.00 V) and charged (2.15 V) states are shown for the different metal sites: (c) Cu K-edge, (d) Fe K-edge, and (e) Zn K-edge.

XAS in an “in situ” configuration was carried out using a pouch cell with an X-ray transparent Kapton window, and the fluorescence was monitored from the back side of the graphite current collector, which can be considered as X-ray transparent (see Figure 6a).

#### 2.6.1 Potentiodynamic XAS during CV Cycling

The XAS–CV cycling studies we refer specifically to as “operando”, and these were carried out in a potentiodynamic fashion where the fluorescence was monitored at a fixed energy during a complete cyclic voltammetric sweep between 1.00 and 2.15 V in 1 M ZnSO_4_. This allowed a fine correlation between the electroactive species and the redox peaks in CuHCF.

The fluorescence was monitored at 8989 eV for the Cu K-edge, at 7126 eV for the Fe K-edge, and at 9660 eV for the Zn K-edge. The derivative of the fluorescence was aligned with the CV curves in a postprocessing step, although the signal from the Keithley instrument was recorded in the analog input of the potentiostat to ensure accurate translation. The data curves shown in Figure 6b are the averages of three consecutive CV cycles to reduce the noise level (no major differences were observed between the consecutive cycles). On the anodic sweep (i.e., during the charging process), the first redox peak at ∼1.75 V (peak A in Figure 6b) can be identified as Cu oxidation. However, there is a weak pre-feature occurring at ∼1.65 V that originates from Fe oxidation. The second main anodic redox peak at ∼1.9 V (peak B) increases with cycling and can be identified as Fe oxidation. However, a weak post-feature at the high-potential side of this peak is also visible at ∼2.0 V, which originates from Cu oxidation. On the cathodic sweep (i.e., during the discharge), the first redox peak at ∼1.7 V (peak C) can be assigned to Fe reduction, while the second peak at ∼1.5 V (peak D) exhibits contributions from mixed Cu and Fe reduction. Another detail we notice is a reductive current starting around 1.1 V (peak E, Figure 6b), which can be assigned to the Cu reduction. There are no potential-dependent changes in the Zn K fluorescence, which suggests that the Zn site is redox-inactive. The mixed contributions from both Cu and Fe electroactive centers to some of the redox peaks explain the bulky shape of the CV profile often seen for CuHCF. The fact that the second peak (peak B) on the anodic sweep increases with cycling can be further confirmed as the activation of the Fe^3+^/Fe^2+^ redox couple, most likely due to the Cu dissolution. A similar activation of Fe sites was reported by Yang et al.^67^ for an analogous Zn–FeHCF hybrid battery. A shift of the galvanostatic charge plateau to higher average potentials have also been reported upon cycling of the Zn/CuHCF cell, which is thought to be linked to formation of nonstoichiometric Zn_*x*_Cu_1–*x*_HCF phases and proposed to have a higher cation insertion potential.^30,32,34^ Here, we postulate a new explanation for the shift of the charge/discharge plateaus to higher potentials, which we emphasize can be explained by the increasing contribution from the Fe^3+^/Fe^2+^ redox couple and the decreasing contribution from the Cu^2+^/Cu^+^ redox couple due to Cu dissolution.

#### 2.6.2 XAS in the Charged/Discharged States

To determine the oxidation states in the charged and discharged states, we collected the K-edges while holding the potential at 1.00 and at 2.15 V during in situ conditions (Figure 6c–e).

In the Cu K-edge XANES in the charged state (2.15 V), there is only one peak at 8986 eV, which signifies Cu^2+^, while in the discharged state (1.00 V), a new peak appears at 8982 eV, associated with Cu^+^. Although, some signal from Cu^2+^ still remains. This suggests the partial reduction of Cu^2+^ → Cu^+^ at 1.00 V. The areas under the respective Cu^+^ and Cu^2+^ peaks are close to 1:1 (Figure S27a); however, we do not make a quantitative estimation of the fractional oxidation states due to the lack of suitable reference compounds.^36^ The Fe site is straightforward since the oxidation state can be obtained from the edge position. In the charged state, we conclude an Fe^3+^ overall oxidation state. The shift of the Fe K edge in the discharged state (at 1.00 V) is consistent with ∼97% of the sites being reduced from Fe^3+^ → Fe^2+^ (Figures 6d and S27b). There are no changes on the Zn site, in agreement with the operando data. Our results are in line with the XAS studies of CuHCF during Li^+^-ion insertion by Mullaliu et al.,^36,65^ where both Cu and Fe sites were confirmed to be electroactive. To the best of our knowledge, there are no in situ XAS studies of CuHCF during Zn^2+^ insertion in an aqueous electrolyte.

The EXAFS simulations are consistent with minor changes in the local atomic structure parameters upon charge/discharge (Figure S28). During discharge (2.15 V → 1.00 V), the Cu and Fe bond lengths are shortened by ∼0.2 Å, which corresponds to a contraction of the lattice parameters by ∼5–10% (Tables S9 and S10). This agrees well with previous data of similar low-strain PBA materials.^31^ There is no change in the local atomic structure parameters around the Zn K-edge, except for a decrease in the coordination number around the Zn–O shell from six to three ligands between the “ex situ” and “in situ” measurements, which we find intriguing (Table S9). We propose that in the “ex situ” measurements, there is a large extent of the dried/crystallized ZnSO_4_ electrolyte in the surface regions of graphite with Zn in an octahedral coordination. In the “in situ” configuration, we instead observe a larger extent of hydrated Zn^2+^ ions occupying the tunnel site in CuHCF, which is analogous to the A site in the ABO_3_ perovskite structure, and known to have a tetrahedral coordination.^29^

### 2.7 Tracking Species in the Charge Compensation Process Using XPS

XPS spectra were also ultimately collected in the charged and discharged states. These electrodes were measured in an “ex situ” configuration; however, the cells were stopped either at 1.00 or 2.15 V before they were disassembled and analyzed.

In the charged state (2.15 V), both Cu^2+^ and Fe^3+^ species are dominating in CuHCF, while in the discharged state (1.00 V), Cu^+^ and Fe^2+^ are the dominating species. When comparing with the in situ XAS results in Figure 6a, it indeed looks as if more Cu sites are electroactive in the surface regions of the CuHCF particles.

**Figure 7:**
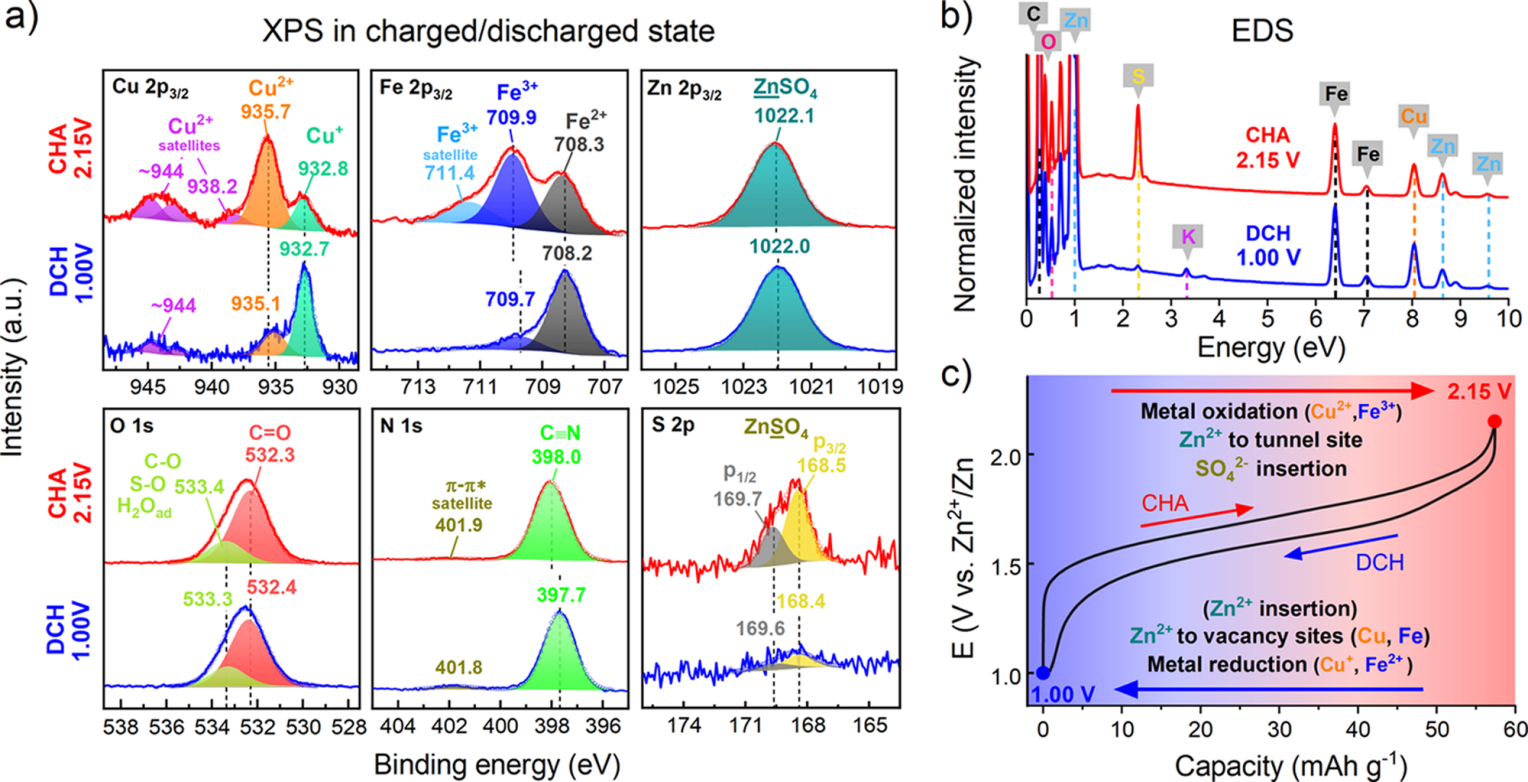
XPS and EDS analyses of CuHCF in the charged and discharged states. (a) XPS spectra of the CuHCF cathode after 200 cycles between 1.00 and 2.15 V in 1 M ZnSO_4_ and stopped either in the charged (2.15 V) or discharged (1.00 V) state. The Cu 2p_3/2_, Fe 2p_3/2_, and Zn 2p_3/2_ spectra are shown on the first row, and the O 1s, N 1s, and S 2p spectra are shown on the second row. (b) EDS spectra of the cycled CuHCF cathode. The cathodes were rinsed with ultrapure water prior to the analysis to remove the excess ZnSO_4_ electrolyte. (c) Summary of the findings regarding the charge compensation mechanism of the CuHCF cathode. The black curve represents the charge and discharge voltage profiles during galvanostatic cycling at 8C rate.

There are no changes in the Zn 2p spectrum, again confirming its redox-inactive nature. Notably, there is also no change in the amount of intercalated Zn^2+^ ions in the CuHCF structure between the charged and discharges states (Figure 7a, top right). This supports the findings by Renman et al.^31^ and the proposed mechanism that charge compensation proceeds via Zn^2+^ swapping positions between cavity/tunnel sites (i.e., 8c sites) and Fe(CN)_6_ vacancy sites (i.e., 4a sites). Herein, we further present evidence that Zn^2+^ ions can also enter Cu vacancy sites (i.e., 4b sites) at the surface of the CuHCF particles due to the extensive Cu dissolution in those regions.

The N 1s peak assigned to the CN ligand shifts by ∼0.3 eV to a lower B.E. in the charged state (2.15 V) (Figure 7a). This was explained earlier as a change in the electron density and the degree of backbonding around the CN ligand. There are no changes in the O 1s spectrum, which excludes the suggestion that oxidic phases are formed at the surface of CuHCF during charge/discharge.

Importantly, the intensity of the S 2p peak (i.e., SO_4_^2–^ ions) is higher during charge (2.15 V), and the peak disappears almost completely during discharge (1.00 V, see Figure 7a, bottom right). Since the CuHCF cathode was rinsed with ultrapure water prior to the analysis, it can be suggested that the SO_4_^2–^ anions can be washed away only in the discharged state. Without this washing step, we did not observe any differences in the S 2p spectra during charge/discharge. This indicates that SO_4_^2–^ ions are inserted into CuHCF during charge where they become specifically adsorbed/coordinated to metal centers. Our results suggest that these anions play a role in the charge compensation process. This may further explain the anion dependence observed for these materials.^32^ EDS analysis confirms these findings and also shows that a small amount of K^+^ ions co-insert into CuHCF during discharge (Figure 7b). Nevertheless, CuHCF is known to insert a variety of cations^68,69^ and may insert any species or impurities present in the electrolyte.

In order to exclude the possibility that SO_4_^2–^ insertion is not related to an intercalation process in graphitic carbon, in analogy with our observations for the XRD peaks, we aslo collected the same set of XPS data for the two carbon “blank” cells (Zn/CB-PVA and Zn/graphite). This concludes that there are no differences in the S content between the charged and discharged states in the carbon blank cells after washing off excess ZnSO_4_ electrolyte (Figures S29 and S30 and Tables S11 and S12), which conclusively assigns the SO_4_^2–^ anion to the charge compensation process in CuHCF. On the other hand, there is some irreversible accumulation of Zn and S, especially in the graphite cathode, which will have to be investigated in more detail in future studies. We tentatively propose that the SO_4_^2–^ ions balance the higher oxidation states of the oxidized Cu^2+^ and Fe^3+^ sites during charge. Our findings are summarized in Figure 7c.

## 3 Conclusions

We provide a highly detailed electrochemical and structural characterization study of the CuHCF cathode employed in aqueous ZIBs. We unravel previously debated aging processes and access new mechanistic findings regarding the charge compensation process. First, we reveal that a set of previously unidentified XRD peaks that appear at early stages of cycling originate from the intercalation of ZnSO_4_ (Zn^2+^/SO_4_^2–^) and/or crystallization of these ionic species in the surface regions of graphitic carbon and are therefore unrelated to CuHCF. We further confirm that Cu is the unstable species and is detrimentally released from CuHCF during cycling. Combined XPS and XAS analysis confirms that Cu dissolution happens mainly in the surface regions of CuHCF, and is spontaneous at the bias imposed at OCP of the Zn/CuHCF cell (∼1.7 V vs Zn^2+^/Zn). Nevertheless, we find that Cu dissolution is not solely responsible for the capacity fade. Therefore, future studies need to target the stability of CuHCF and PBAs in more detail to better understand correlations between cycling and effective performance losses in this type of aqueous ZIBs. Using in situ XAS, we confirm that both the Cu^2+^/Cu^+^ and Fe^3+^/Fe^2+^ redox couples participate in the charge compensation process, although the Cu redox couple is more active at the surface—hence correlated to the Cu vacancies. We conclude that Zn^2+^ ions can enter these vacant Cu sites in addition to the native Fe(CN)_6_ vacancies already present in the structure. Potentiodynamic XAS coupled with cyclic voltammetry establishes a direct link between redox-active metal centers and the voltammetric redox peaks. We thereby conclude that the Fe^3+^/Fe^2+^ couple is activated during repeated cycling due to the Cu dissolution, and subsequently, the loss of the Cu^2+^/Cu^+^ couple. The Fe redox couple being located at higher average potentials than the Cu redox couple further explains the progressive increase in the voltage of the anodic redox peak and, analogously, the increase in the potential of the charge/discharge plateaus upon repeated cycling. Finally, we discover that SO_4_^2–^ anions participate in the charge compensation process in addition to Zn^2+^ions, which reversibly insert into CuHCF during charge. Our study establishes a profound understanding of the aging and the charge compensation processes that impact both directly and indirectly the performance of the aqueous Zn/CuHCF cell, which are deemed crucial from the perspective of the strategic design of future PBA-type cathodes for ZIBs and possible improvements of this attractive, yet challenging, Zn-ion rechargeable technology.

## Supporting Information Available

Experimental procedure (synthesis, electrode cast and assembly of Zn-ion cells, electrochemical characterization, physical characterization, XPS, elemental analysis, XAS analysis, and EXAFS simulations); calculations of the gravimetric capacity, Coulombic efficiency, round-trip efficiency, and electron transfer number; TGA curve and SEM image of the pristine CuHCF powder; electrochemical characterization, EDS spectra, and elemental compositions from the EDS of the Zn/CuHCF cell; recovery test combined with cell cycling; XRD patterns of the cathode side of the Zn/CuHCF cell; metal loadings of the components of the Zn/CuHCF cell; ICP-OES of the Zn anode; SEM-EDS elemental mapping of the Zn/CuHCF cell after 0 and 200 cycles; ICP-OES study and aging characteristics of the CuHCF cathode; impact of the electrolyte concentration on the CuHCF cathode performance and stability; HAADF-STEM and EDS elemental maps of the pristine CuHCF cathode and the CuHCF cathode after 200 cycles; XRD patterns and EDS analysis of the cathodes in carbon “blank” cells; CV of the carbon “blank” cells: Raman spectra of the Zn/graphite cell; SEM images of the washed and unwashed cathodes; XPS spectra and elemental compositions of the CuHCF cathode under different conditions; edge positions and oxidation states from K-edge XAS; EXAFS simulations and fit parameters; FT-EXAFS of the CuHCF cathode; and EDS spectra and elemental compositions of two carbon “blank” cathodes (PDF)
