# Supplementary material for: Aging and Charge Compensation Effects of the Rechargeable Aqueous Zinc/Copper Hexacyanoferrate Battery Elucidated Using In Situ X-ray Techniques
Source: ACS Appl Mater Interfaces. 2021 Dec 8;13(50):59962–74. doi: 10.1021/acsami.1c19167 (PMC8704201; doi:10.1021/acsami.1c19167)
Supplement: Supplementary file 1 — am1c19167_si_001.pdf [file am1c19167_si_001.pdf]

ACS APPLIED MATERIALS & INTERFACES

Supporting Information

Aging and Charge Compensation Effects of the Rechargeable Aqueous Zinc/Copper Hexacyanoferrate Battery Elucidated Using In Situ X-ray Techniques

Mikaela Görlin,*,†,‖ Dickson O. Ojwang,† Ming-Tao Lee,† Viktor Renman,‡ Cheuk-Wai Tai,§ and Mario Valvo*,†

† Department of Chemistry- Ångström Laboratory, Uppsala University, Box 538, SE-75121 Uppsala, Sweden

‡ Department of Materials Science and Engineering, Norwegian University of Science and Technology, NO-7491 Trondheim, Norway

§ Department of Materials and Environmental Chemistry, Stockholm University, SE-106 91 Stockholm, Sweden

Corresponding authors:

***E-mail**: mikaela.gorlin@kemi.uu.se
***E-mail**: mario.valvo@kemi.uu.se

Present address:

‖ Department of Mechanical Engineering, Research Laboratory of Electronics, Massachusetts Institute of Technology, Cambridge, Massachusetts 02139, United States

Experimental section

Synthesis, Chemicals and materials

Anode: Zn foil (99.98% metals basis, 0.25 mm thick, AlfaAesar); Cathode: slurry comprised of depotassiated copper hexacyanoferrate (CuHCF) powder following an earlier procedure.[1] Briefly, the synthesis of depotassiated CuHCF was carried out from 50 mL of solutions (degassed with N_2) of 0.08 M $CuSO_4$ and 0.04 M $K_3Fe(CN)_6$, which were simultaneously and rapidly mixed into 25 mL of distilled water at room temperature under constant stirring. The reaction was left for ca. 30 min under a protective N_2 atmosphere before the precipitate was collected and washed 5 times with distilled water (also degassed with N_2) using centrifugation. The precipitate (i.e., CuHCF nanoparticles) was dried in a vacuum oven at 60 °C for ca. 12 h. All chemicals were of analytical purity grade unless otherwise stated, purchased from Sigma-Aldrich. Polyvinyl alcohol (PVA, – 18-88, Fluka) was used as binder solution, carbon black (CB) as conductive additive (C·ENERGY-SuperC65, IMERYS), and graphite foil (Sigratherm®F, 1.3 g cm^{-3}, thickness 120 µm, SGL carbon) as current collector and contact tabs. The 1M $ZnSO_4$ electrolyte was prepared from $ZnSO_4 \cdot 7H_2O$ (99.995 % trace metals basis, Sigma-Aldrich) and diluted using ultrapure water (Water Blank, ASTM Type I 18 MΩ, SPEX CertiPrep™).

Electrode cast and assembly of Zn-ion cells

CuHCF slurry was prepared by mixing CuHCF (0.3 g), carbon black (0.04 g), aqueous PVA binder solution (1.2 mL, 5 wt %), and isopropanol (0.25 mL); thus, yielding a composition of 75:10:15 wt.% of CuHCF:CB:PVA. The slurry was homogenized for 1 h at 100 rpm using a planetary ball mill (RETSCH PM 4). The slurry was then bar-coated onto graphite foil with a wire-wound meter bar (100 µm wet film deposit, K Control Coater). The slurry-coated graphite foil was left to settle for ca. 5 min, and then dried in a convection oven at 60 °C for ca. 1 h. Circular electrodes, each containing ca. 1.5 mg of active material, were cut using a hole puncher (ø 13 mm). Two pieces of filter paper (Munktell) were employed as separator, and these were punched into slightly larger disks (ø 20 mm). Before assembling the cell, the Zn foil was polished using sandpaper in order to remove surface oxide layer. After sanding, the Zn disks were sonicated in ethanol for ca. 2 min and then dried with Ar gas. Prior to assembling the pouch cells, the 1 M $ZnSO_4$ electrolyte was degassed with Ar for ca. 15 min to minimize the amount of dissolved O_2. Note that we cannot guarantee a completely air free environment in the electrolyte since the pouch cells were assembled at ambient conditions. Pouch-cells made of a polymer-aluminum laminate was assembled as follows: the CuHCF/CB/PVA cathode on one side, two filter paper separators in between, and the Zn foil on the anode side, and 80 µL of 1 M $ZnSO_4$ electrolytic solution. Two strips of graphite sheet were placed on each side of the cathode/anode as electrical contact, and the pouch cell was subsequently sealed using a vacuum sealer to remove the air from the inside. To ensure proper contact of the various parts and to apply a uniform pressure, the Zn/CuHCF cell was clamped between two sturdy plates.

Electrochemical characterization

A multi-channel potentiostat (MPG-2, Bio-Logic) was used to perform the electrochemical characterization. Either galvanostatic cycling with potential limitation (GCPL) or cyclic voltammetry (CV) were employed. CV cycling was carried out up to 200 cycles between vertex potentials of 1.00 V to 2.15 V *vs.* Zn^{2+}/Zn in 1 M $ZnSO_4$, pH 4.8. For "aging" studies, the cells were stopped at open circuit potential (OCP), and for charge compensation studies, the cells were stopped either in the discharged state (1.00 V) or in the charged state (2.15 V). The uncompensated solution resistance (R_u), which we refer to as the cell resistance, was determined using electrochemical impedance spectroscopy (EIS) between 20 kHz – 10 mHz (sinus amplitude of 10 mV) before and after cycling. A typical value for R_u in

the cell is 10 Ω. If not stated otherwise, the cells were opened under ambient conditions before analyses, and the anode and cathode were washed thoroughly by dipping them into fresh ultrapure water ca. 5 times for a few seconds, and then immediately dried with Ar gas. A few cells were also analyzed without this washing step, which is then explicitly stated in the text.

Physical characterization

The water content and the molecular weight of the CuHCF material were determined using a TGA-Q500 thermogravimetric analyzer (TA Instruments). An amount of ca. 5-6 mg of the pristine CuHCF powder (without binder or carbon) was placed in aluminum pans. The samples were equilibrated at 30 °C prior to ramping the temperature to 500 °C at a heating rate of 10 °C min^{-1} under N_2 flow.

Grazing incidence X-ray diffraction (XRD) of the CuHCF cathodes was performed using a Siemens D5000 diffractometer in parallel beam mode with Soller slits (0.40°), and 1 mm in and outgoing slit. The diffractometer was operated at 45 kV and 40 mA. The diffractograms were recorded in an angular range between 5-70 ° (2θ), with a step size of 0.035°, and 28 s per step. The samples were placed onto dedicated monocrystalline Si plates with zero-background.

Scanning electron microscopy (SEM) was carried out using a LEO 1550 microscope (ZEISS), equipped with an energy dispersive X-ray spectrometer (EDS) from Oxford Instruments. Images were recorded with a typical acceleration voltage of 3 - 5 kV, while the EDS spectra were conducted with a voltage of 15 kV. Post processing of SEM-EDS elemental maps was performed using the AZtec software (Oxford Instruments).

Transmission electron microscopy (TEM) was carried out using a Thermo Fisher Scientific Themis Z microscope at the Electron Microscopy Centre (EMC) at the department of Materials and Environmental Chemistry (MMK), Arrhenius Laboratory, Stockholm University. The samples were scratched off from the substrate with a diamond scriber onto a TEM grid (SPI Supplies 200 mesh Cu grid with holey carbon supporting films). The microscope has both probe and image spherical aberration (C_s) correctors and Super-X Si drift detectors for EDS analysis that cover all angles of the sample. High-angle annular dark-field (HAADF) and bright-field (BF) scanning TEM (STEM) images were acquired simultaneously. The STEM and EDS analyses were performed at 60 kV. The probe current was 150 pA. The convergent angle of electron probe was 18.3 mrad. The collection angle of the BF and HAADF detector was up to 22 mrad and from 50 to 200 mrad, respectively. Post processing of the images and EDS elemental maps was performed in Velox (Thermo Fisher Scientific B. V.).

X-ray photoelectron spectroscopy

X-ray photoelectron spectroscopy (XPS) spectra were acquired with a PHI 5500 spectrometer (Physical Electronics) using monochromatic Al Kα radiation (1486.7 eV) and an electron emission angle of 45 °. XPS spectra were collected *post mortem* at the Cu 2p, Fe 2p, Zn 2p, C 1s, O 1s, N 1s, and S 1s edges, for pristine electrodes (not exposed to electrolyte), for electrodes cycled for a different number of cycles and stopped at OCP (for "aging" studies), and for electrodes cycled for 200 cycles and then stopped in either the charged (CHA) or the discharged (DCH) states (for studies of the charge compensation process). XPS spectra were also recorded for carbon "blank" cells, in which the active material (CuHCF) was left out. The spectra containing graphitic carbon were energy calibrated to the sp^2-C peak of the carbon at 284.3 eV, while other spectra were calibrated to the adventitious carbon peak at 284.8 eV.[2,3] The XPS chamber pressure was kept at 1×10^{-7} Torr or lower pressures during the sample analysis. The acquired spectra were analyzed using the CasaXPS software and considering

typical a Gaussian/Lorentzian lineshape (30 % Lorentzian contribution) and employing a (Shirley-type) background. The sp^2 component in graphite was fitted using an asymmetric finite Lorentzian (LF) lineshape numerically convoluted with a Gaussian, LF(α, β, w, m), where α and β are parameters that modify the curvature of the Lorentzian, w is the width parameter of the smooth function, and m is the width parameter of the Gaussian. The parameters were minimized in order to achieve the best fit with the data, and were following; $\alpha = 0.95$, $\beta = 1.6$, w = 100, m = 80. The LF lineshape is equivalent to the Doniach-Sunjic lineshape, except that it has a finite background which is practical when using standard backgrounds such as Shirley type.

Elemental analysis

The elemental compositions and metal loadings were determined using inductively coupled plasma – optical emission spectroscopy (ICP-OES), using an Avio 200 spectrometer (Perkin-Elmer). The sample preparation was carried out as follows; the CuHCF samples (ca. 10 mg powder or the circular disc electrode containing ca. 1.5 mg of active material) was first heated to 500 °C in a furnace (the temperature was ramped at 5 °C min^{-1}) for 6 h to convert the copper hexacyanoferrate phase into an oxide phase to facilitate digestion with acid. Afterwards, the samples were digested in a mixture of 0.5 mL HNO_3 (65%, EMSURE, Merck), 1 mL H_2SO_4 (ISO, 95-97%, Merck) and 1.5 mL HCl (37 %, ACS reagent Merck) into their metallic components. The samples were then diluted with ultrapure water up to a final volume of 200 mL. The emission wavelengths were selected as follows; Cu 327.393 nm, Fe 238.204 nm, Zn 206.200 nm, and K 766.490 nm. A multi-element standard solution containing 10 mg L^{-1} of the elements dissolved in 5% HNO_3 (Perkin-Elmer) was used for calibration curve.

X-ray absorption spectroscopy (XAS)

X-ray absorption spectroscopy (XAS) was measured at the Cu, Fe, and Zn *K*-edges of the CuHCF cathode at the KMC-3 beamline at the BESSY II synchrotron facility, Helmholtz Zentrum Berlin (HZB), Germany. A scintillation detector coupled to a photomultiplier was employed to collect the fluorescence. The detector was covered with a foil one element below the investigated *K*-edge (Z-1) to reduce scattered light and to improve the signal. The Zn/CuHCF cell was investigated "ex situ" and "operando". The cathodes investigated ex situ comprised the electrode in its pristine state, and electrodes cycled for 0, 100, and 200 cycles in 1 M $ZnSO_4$ in pouch cells, and then stopped at OCP, which corresponds to ca. ~1.7 V vs. Zn^{2+}/Zn. The electrode parts were taken out immediately after stopping the cells, rinsed with ultrapure water, and immediately dried with Ar-gas. The operando measurements were performed in real-time conditions while cycling the Zn/CuHCF in a pouch cell. The pouch cell had been modified with an X-ray transparent Kapton window, and the fluorescence was monitored from the backside of the CuHCF cathode through the graphite current collector. Spectra were collected while holding the potential in either the discharged state (DCH, 1.00 V) or in the charged state (CHA, 2.15 V). Potentiodynamic measurements were also performed, where we followed the fluorescence during cyclic voltammetry. For these dynamic measurements, the fluorescence was recorded by connecting the output of the Keithley instrument to the analog input of the Bio-Logic SP-200 potentiostat, to ensure accurate translation of the fluorescence signal and the voltage in time. The fluorescence was monitored around the *K*-edge position, where largest changes were observed; 8989 eV for the Cu *K*-edge, 7126 eV for the Fe *K*-edge, and 9660 eV for the Zn *K*-edge. The local atomic structure was determined by simulations of the extended X-ray absorption fine structure (EXAFS) using scattering function generated in FEFF 9.1.[4] The simulation approach was adopted from earlier studies, and will be described briefly hetein.[5–7] Given that the Cu/Fe ratio is ~1.5 in the pristine CuHCF powder samples, there should be 1/3rd of $Fe(CN)_6$ vacancies in the native CuHCF structure. These vacancies were

confirmed to be occupied by zeolitic and/or coordinating water´s in neutron diffraction experiments by Wardecki et al.[5] In our EXAFS simulations on the Cu *K*-edge, we therefore represented these $Fe(CN)_6$ vacancies with a Cu-O shell, where the O represent the water molecules. Multiple scattering was considered up to 4-leg path due to the strong focusing effect from the linear Fe-CN-Cu chains.

Calculations of gravimetric capacity, Coulombic efficiency, round-trip efficiency, and electrons transferred in the electrochemical process

The practical charge/discharge capacity ($Q_{cha/dch}$) of the active material was determined from the galvanostatic charge/discharge voltage profiles according to Equation (S1):

$$Q_{cha/dch} = \frac{i \cdot t}{m} \tag{S1}$$

where $Q_{cha/dch}$ is the capacity during charge or discharge expressed in mAh g^{-1}, i is the applied current in mA, t is the time in hours, and m is the mass of the active material in grams determined by ICP-OES.

The theoretical capacity ($Q_{theoretical}$) is calculated according to Equation (S2):

$$Q_{theoretical} = \frac{n \cdot \mathrm{F}}{3.6 \cdot \mathrm{M_w}} \tag{S2}$$

where n is the "electron transfer number" (i.e., moles of e^- transferred in the redox-process per moles of redox-active metal sites (in this case (Cu+Fe)),[8] F is the Faraday constant (96485.33 C mol^{-1}), and M_w is the molecular weight of CuHCF determined from TGA analysis (267.07 g mol^{-1}) assuming a molar formula of K:Cu:Fe:C:N of 0.05:1.00:0.66:4:4 and 3.3 water molecules per unit formula.[9]

Since the capacity strongly depends on the value of n (i.e., the electrons transfer number), which varies for different cycling rates, we instead use the term "expected" capacity at different cycling rates, which is calculated according to Equations (S3)-(S4):

$$Q_{\mathrm{expected\ @\ 8C}} = \frac{n \cdot \mathrm{F}}{3.6 \cdot \mathrm{M_w}} = \frac{0.54 \cdot 96485.33}{3.6 \cdot 264.55} = 55\ (\pm 10)\ \mathrm{mAh\ g^{-1}} \tag{S3}$$

$$Q_{\mathrm{expected\ @\ 1C}} = \frac{n \cdot \mathrm{F}}{3.6 \cdot \mathrm{M_w}} = \frac{0.78 \cdot 96485.33}{3.6 \cdot 264.55} = 79\ (\pm 6)\ \mathrm{mAh\ g^{-1}} \tag{S4}$$

where n is the electron transfer number (see Equation S5 below), M_w is the molecular weight of CuHCF determined by TGA analysis (267.07 g mol^{-1}), and F is the Faraday constant. The difference in expected capacities between 8C and 1C rates is thus ~25 mAh g^{-1}, which is in good agreement with our measured practical charge/discharge capacities at these rates. The C-rate (i.e., "N·C-rate") refers to the current (i) applied to withdraw the entire electrode capacity in (1/N) hours, and is evaluated based on the initial reported theoretical capacity of CuHCF (i.e., 60 mAh g^{-1}) and its active mass found in the coated electrodes.[10]

The electron transfer number (n) used in Equation (S3-(S4), i.e., the number of electrons transferred per of (Cu+Fe) sites, was determined from the cyclic voltammograms according to Equation (S5):

$$n = \frac{\int i \cdot dt}{F \cdot n_{\mathrm{Cu+Fe}}} \tag{S5}$$

where $i \cdot dt$ is the integrated area under the redox peaks (i.e., the charge in units C), i is the current in A, F is the Faraday constant, and $n_{\mathrm{Cu+Fe}}$ is the moles of total metal (i.e., moles of Cu and Fe) on the

electrode determined from ICP-OES. At 1C and 8C, we estimate that 0.78 ± 0.09 and 0.54 ± 0.10 e^- are transferred across the interface (see Figure S2c-d).

The Coulombic efficiency ($Q_{eff.}$) was calculated for each cycle according to the expression in Equation (S6):

$$Q_{eff.} = \frac{Q_{dch}}{Q_{cha}} \text{ x } 100\ (\%) \tag{S6}$$

Where Q_{dch} is the discharge capacity and Q_{cha} the charge capacity. The round-trip efficiency ($U_{eff.}$) was also calculated for each cycle via an expression analogous to Equation (S6), however, the ratio is instead U_{dch}/U_{cha}, which represents the respective discharge and charge energies at the end of each half-cycle expressed in units of mWh.

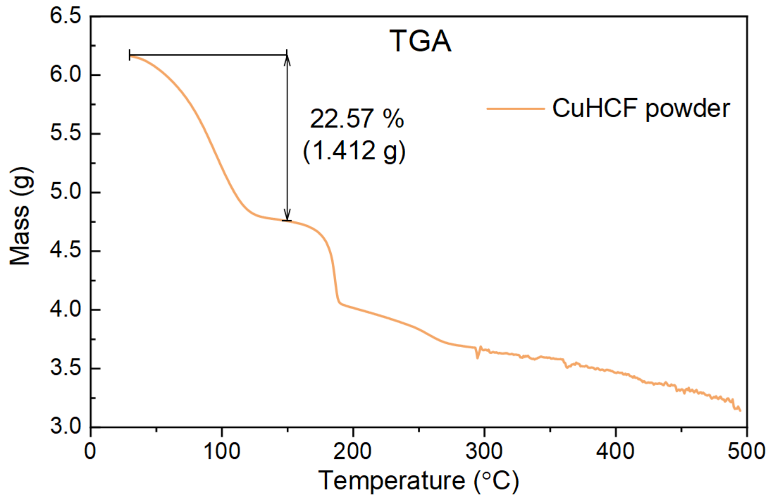

Figure S1. Thermogravimetric analysis (TGA) of the pristine CuHCF powder sample without additions of PVA binder or carbon black (CB). Note that the initial mass loss upon heating from room temperature to about 120 °C is due to release of water from the CuHCF structure. The water loss from TGA analysis was estimated to 22.57 wt. %, which corresponds to 3.3 water molecules per unit formula and, a molecular weight of 267.07 g mol^{-1}, assuming a molar formula of K:Cu:Fe:C:N of 0.05:1.00:0.66:4.00:4.00.[9]

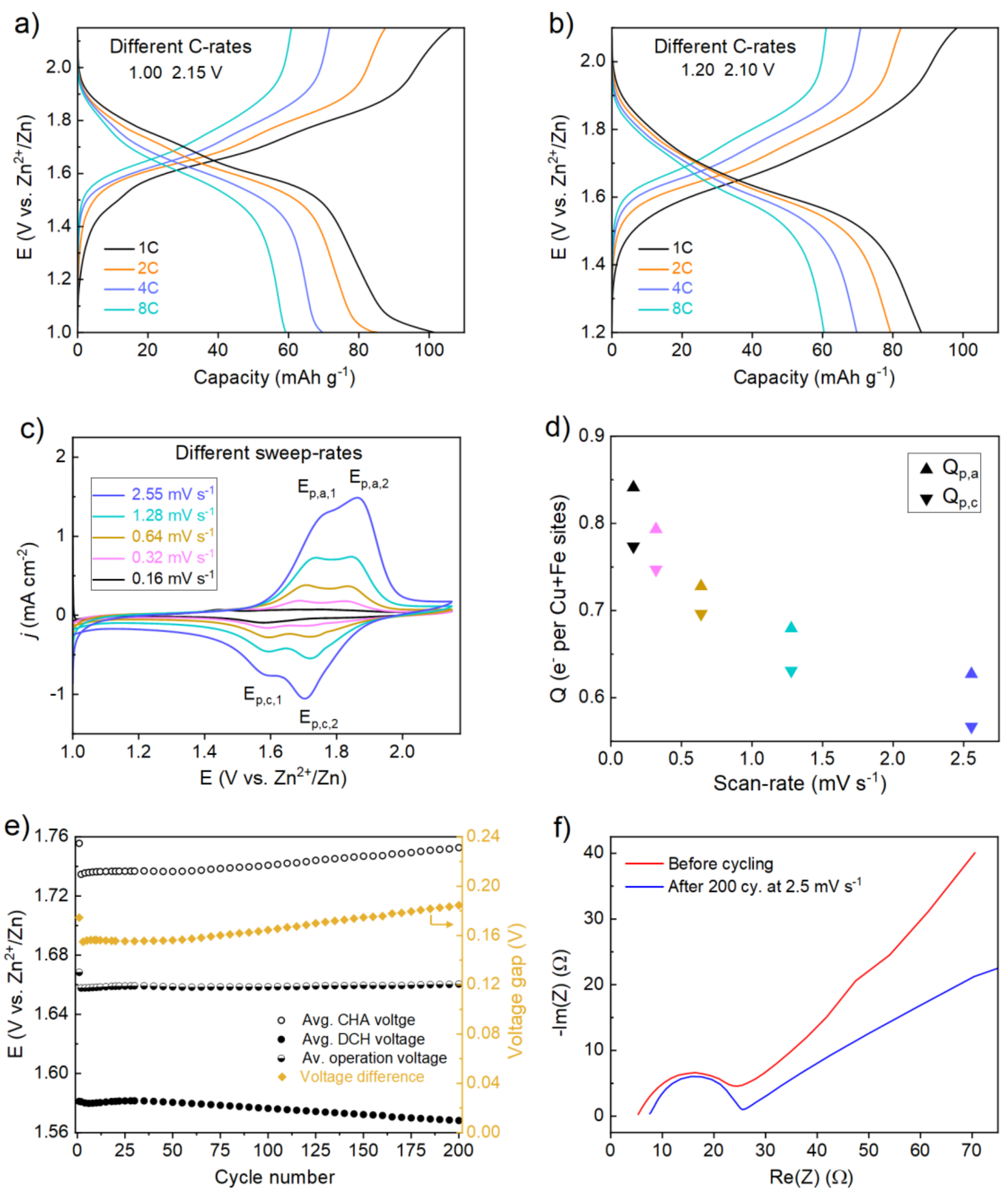

Figure S2. Electrochemical characterization of the Zn/CuHCF cell in 1 M $ZnSO_4$ **(a)** Galvanostatic charge/discharge cycles at different C-rates (1C, 2C, 4C, and 8C) between 1.00 – 2.15 V **(b)** The corresponding galvanostatic cycles with a narrower potential window 1.20 – 2.10 V **(c)** CVs at different scan-rates (0.16, 0.32, 0.64, 1.28, and 2.55 mV s^{-1}) **(d)** The electron transfer number, i.e., the e^- transferred per Cu+Fe site, obtained from the redox-peak charge (Q_p) by integration of the CVs, both for the anodic ($Q_{p,a}$) and the cathodic ($Q_{p,c}$) peaks. The total area under both redox-features ($E_{p,1}$ and $E_{p,2}$) was included in these calculations. **(e)** Evolution of average charge/discharge voltages weighted to their respective capacities, together with the voltage difference and corresponding average cell operating voltage per cycle, obtained from the galvanostatic charge/discharge measurements. **(f)** Electrochemical impedance spectroscopy (EIS) before and after 200 cycles. Note that at the upper/lower voltages of the charge/discharge curves in (a), the curves start sloping. We regard this to the oxygen evolution reaction (OER) and the oxygen reduction reaction (ORR), respectively, based on the fact that the thermodynamic feasible onset potentials for these reactions lie within the potential window (1.00 V – 2.15 V vs Zn^{2+}/Zn = 0.52 V – 1.67 V vs. RHE in 1M $ZnSO_4$ pH 4.8).[11,12] It is therefore desirable to cycle this system with a slightly narrower potential window, especially at slow cycling rates.

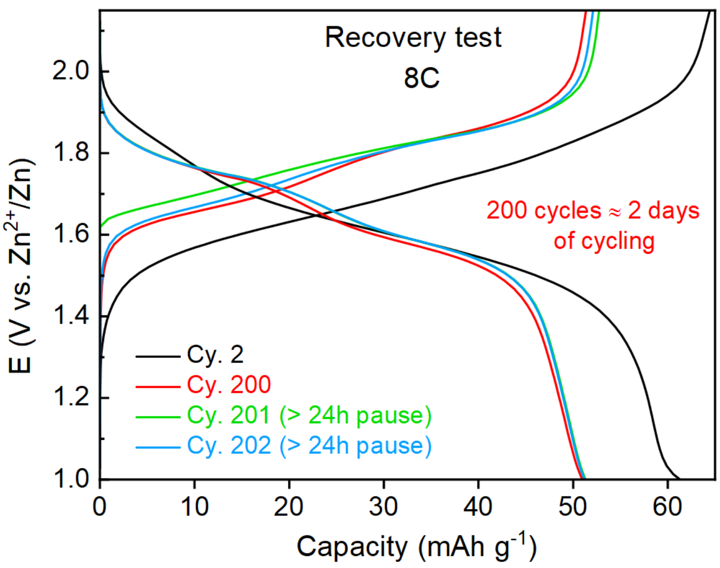

Figure S3. Recovery test combined with cell cycling by introducing a 24 h pause at the end of 200 galvanostatic cycles at 8C between 1.00 – 2.15 V vs Zn^{2+}/Zn. Cycle 2 and 200 are shown before the pause, and then the 1st and 2nd cycle after the 24 h long intentional pause (i.e., cycle 201 and 202). We did not observe any recovery after the pause, whereby we conclude that the capacity loss is permanent.

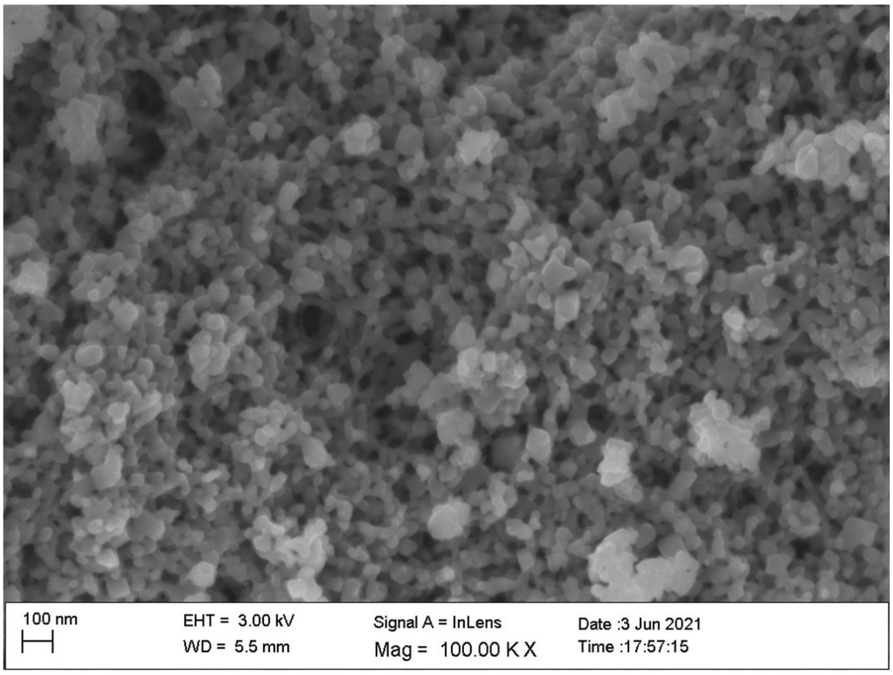

Figure S4. SEM image of the pristine CuHCF particles before electrode coating (i.e., without any addition of CB or PVA binder).

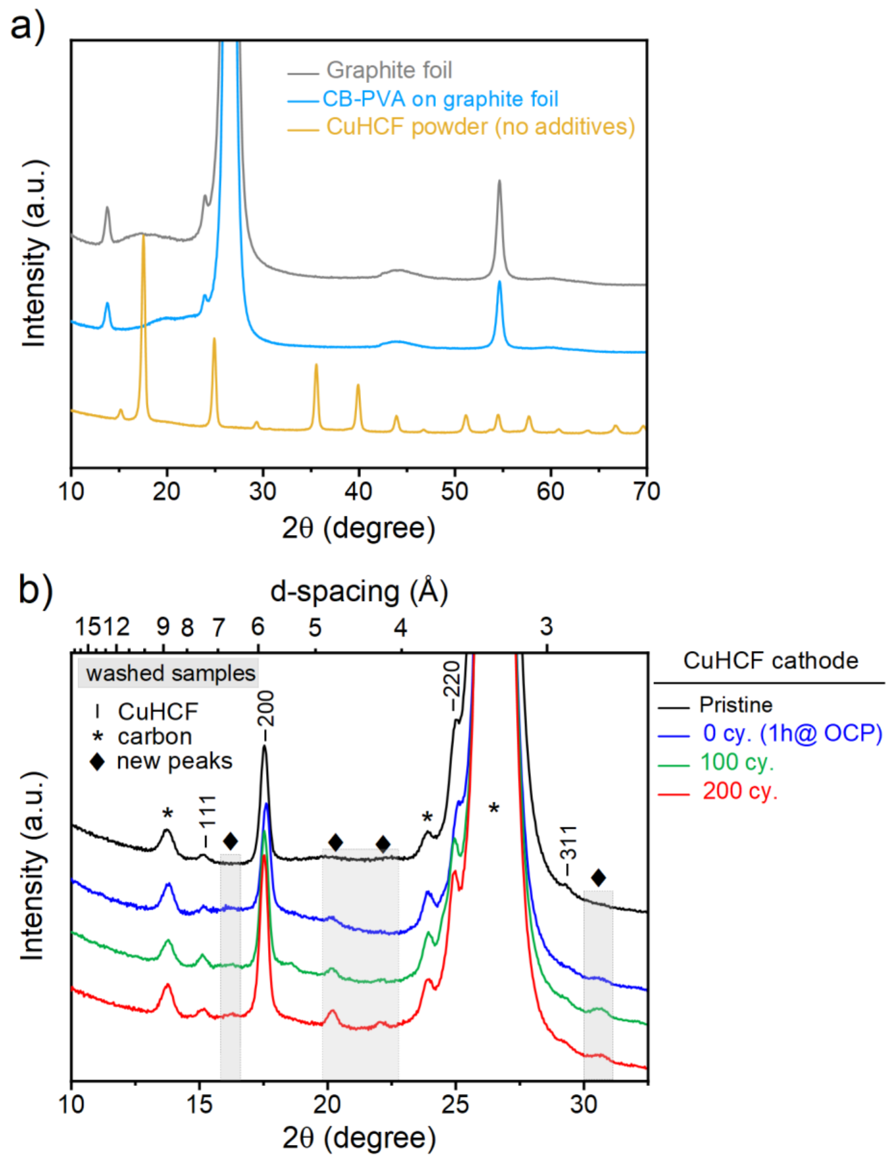

Figure S5. X-ray diffraction patterns of the cathode side of the Zn/CuHCF cell. **(a)** The pure CuHCF powder (without additions of CB or PVA), the CB-PVA cast on graphite foil, and the bare graphite foil. **(b)** The CuHCF cathode after different stages of CV cycling between 1.00 - 2.15 V vs. Zn^{2+}/Zn at 2.5 mV s^{-1} in 1M $ZnSO_4$. The diffraction patterns of following: the pristine cathode cast on graphite foil (also containing CB and PVA), after 0 cycles (i.e., after resting the cell for 1 h at OCP), after 100 cycles, and after 200 cycles. All cycled electrodes were stopped at OCP and rinsed with ultrapure water before analyzed to remove excess of $ZnSO_4$ electrolyte. New diffractions peaks become visible at 18.5°, 20.2°, 22°, and 30.5° (marked with diamonds). The diffraction peaks associated with graphitic carbon are marked with asterisks.

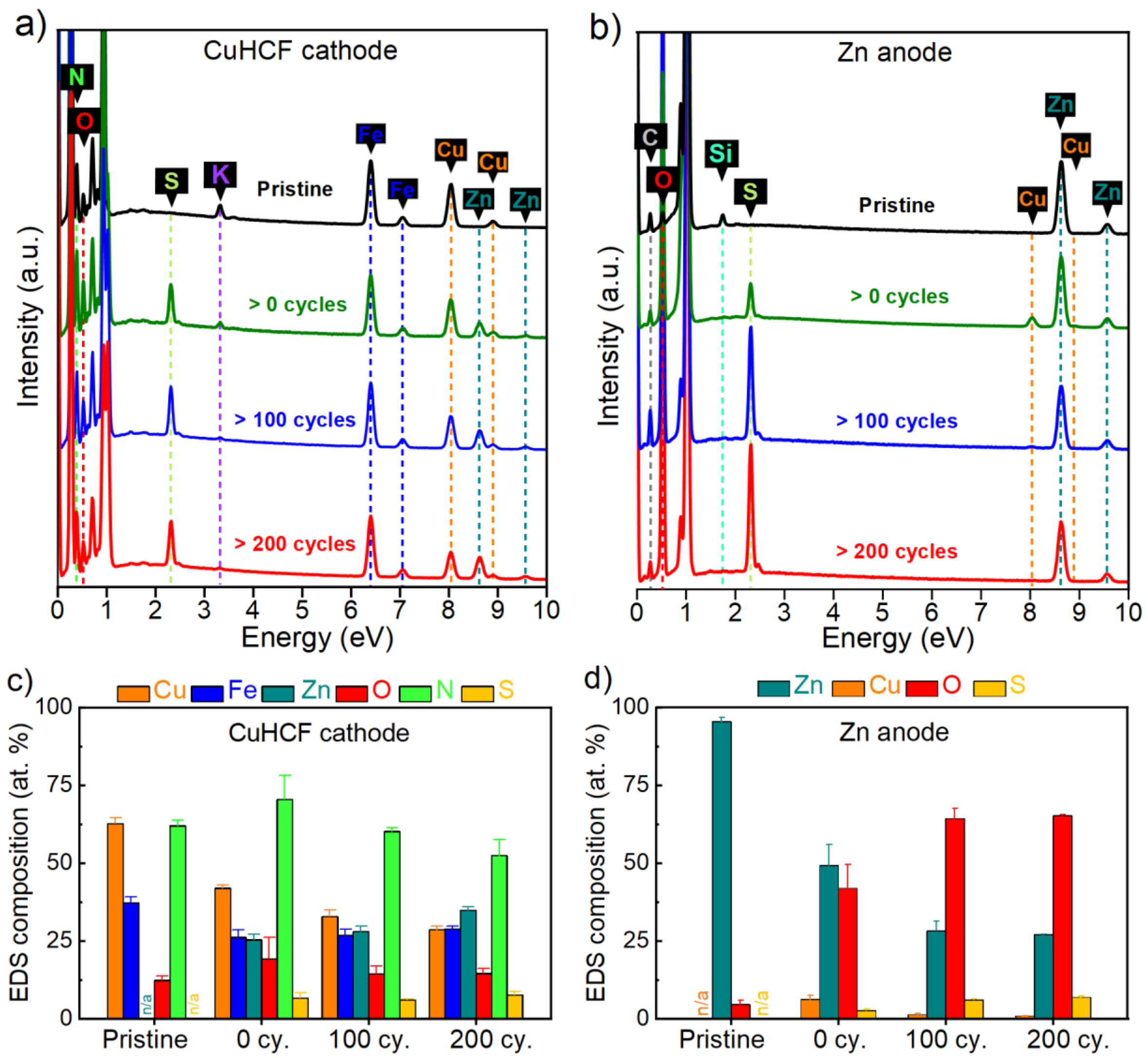

Figure S6. Energy dispersive X-ray spectroscopy (EDS) of the Zn/CuHCF cell at various stages of CV cycling (pristine, 0 cy., 100 cy., 200 cy.) between 1.00 V - 2.15 V *vs.* Zn^{2+}/Zn at a scan-rate of 2.5 mV s^{-1} in 1M $ZnSO_4$. **(a)** EDS spectra of the CuHCF cathode **(b)** EDS spectra of the corresponding Zn anode. **(c)** The atomic compositions of the CuHCF cathode at the various stages of cycling. **(d)** The corresponding composition for the Zn anode. All cells were stopped at OCP (~1.7 V *vs.* Zn^{2+}/Zn), disassembled, and rinsed with ultrapure water to remove excess of electrolyte before being analyzed. The shown states are the pristine electrode (not exposed to electrolyte), after 0 cycles (resting the cell for 1h at OCP), after 100 cycles, and after 200 cycles in 1 M $ZnSO_4$.

Table S1. Elemental compositions from EDS of the Zn/CuHCF cell after various stages of CV cycling between 1.00 - 2.15 V at 2.5 mV s^{-1} in 1M $ZnSO_4$ electrolyte. All cells were stopped at OCP, opened, and rinsed with ultrapure water, and dried with Ar-gas prior to analysis to remove excess of electrolyte. The standard errors given in brackets were estimated from the overall number of measurements of repetitions. The carbon content (from the support and current collector) has been left out to allow comparison of the relative changes associated with the active material (CuHCF).

**Washed samples**	**Elemental compositions from EDS (at. %) Zn/CuHCF cell**							
**CuHCF cathode**	**K**	**Cu**	**Fe**	**Zn**	**S**	**O**	**N**	**Si**
Pristine	2.5 (0.1)	62.7 (1.9)	37.6 (1.9)	n/a	n/a	12.3 (1.4)	62.0 (1.8)	n/a
0 cycles	0.8 (0.1)	41.9 (1.0)	26.1 (2.4)	25.3 (1.8)	6.6 (1.7)	19.2 (6.9)	70.5 (7.8)	n/a
100 cycles	0.3 (0.1)	32.9 (3.0)	26.8 (2.7)	27.9 (1.5)	6.1 (0.3)	14.4 (2.4)	60.2 (1.1)	n/a
200 cycles	0.2 (0.1)	28.6 (1.0)	28.9 (0.8)	34.3 (1.2)	7.7 (1.0)	14.5 (1.6)	52.4 (5.1)	n/a
**Zn anode**	**K**	**Cu**	**Fe**	**Zn**	**S**	**O**	**N**	**Si**
Pristine	n/a	n/a	n/a	95.4 (1.3)	n/a	4.6 (1.3)	n/a	1.9 (1.2)
0 cycles	n/a	6.2 (1.4)	n/a	49.2 (6.6)	2.7 (0.4)	41.9 (7.6)	n/a	n/a
100 cycles	n/a	1.4 (0.3)	n/a	28.2 (3.1)	6.1 (0.3)	64.4 (3.1)	n/a	n/a
200 cycles	n/a	0.9 (0.1)	n/a	27.0 (0.1)	6.9 (0.4)	65.2 (0.2)	n/a	n/a

Table S2. Metal loadings of the components of the Zn/CuHCF cell obtained from ICP-OES (cathode, separator, anode). The investigated states are following: pristine (not exposed to electrolyte, not cycled), and after 200 CV cycles between 1.00 - 2.15 V at 2.5 mV s^{-1} in 1M $ZnSO_4$, and stopped at OCP. Note that the cell parts were analyzed without rinsing with water (i.e., “as is”), to facilitate identifying the location of species, also the ionic ones. Thus, the Zn-content here is very high since it also originates from dried $ZnSO_4$ electrolyte. The standard errors given in brackets were obtained from the overall number of measurements.

**Unwashed samples**	**Metal content from ICP-OES (µmol)**			
	**K**	**Cu**	**Fe**	**Zn**
CuHCF cathode (pristine)	0.12 (0.03)	5.19 (0.40)	3.36 (0.36)	n/a
Separator (pristine)	n/a	n/a	n/a	n/a
Zn anode (pristine)	n/a	0.05 (0.01)	0.03 (0.01)	627.0 (1.0) [a)]
CuHCF cathode (200 cycles)	0.09 (0.01)	3.28 (0.01)	3.43 (0.03)	25.3 (0.5) [a)]
Separator (200 cycles)	0.02 (0.01)	0.06 (0.01)	0.09 (0.01)	81.2 (0.2) [a)]
Zn anode (200 cycles)	0.01 (0.01)	1.91 (0.01)	0.04 (0.01)	618.9 (0.7) [a)]
a) The cell parts were analyzed “as is”, i.e., they were not rinsed with water prior to analysis, and therefore the high Zn content originates from the dried $ZnSO_4$ electrolyte.				

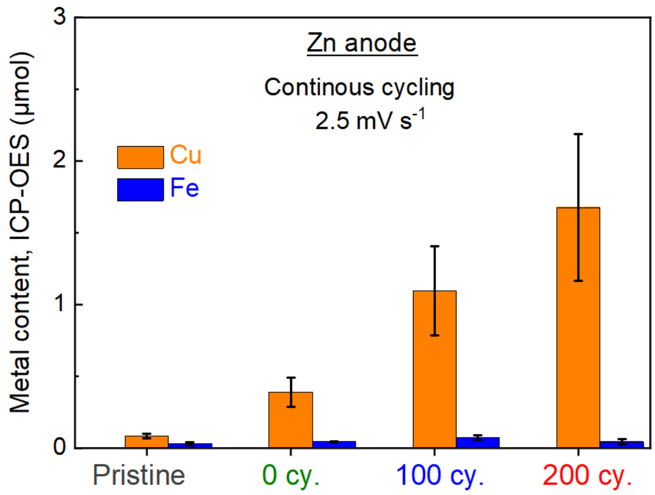

Figure S7. ICP-OES of the Zn anode after different stages of cycling; pristine, 0 cycles (1 h resting at OCP)**,** 100, and after 200 cycles between 1.00 – 2.15 V in 1 M $ZnSO_4$, and stopped at OCP. The error bars represent the standard error from repetitions of several electrodes.

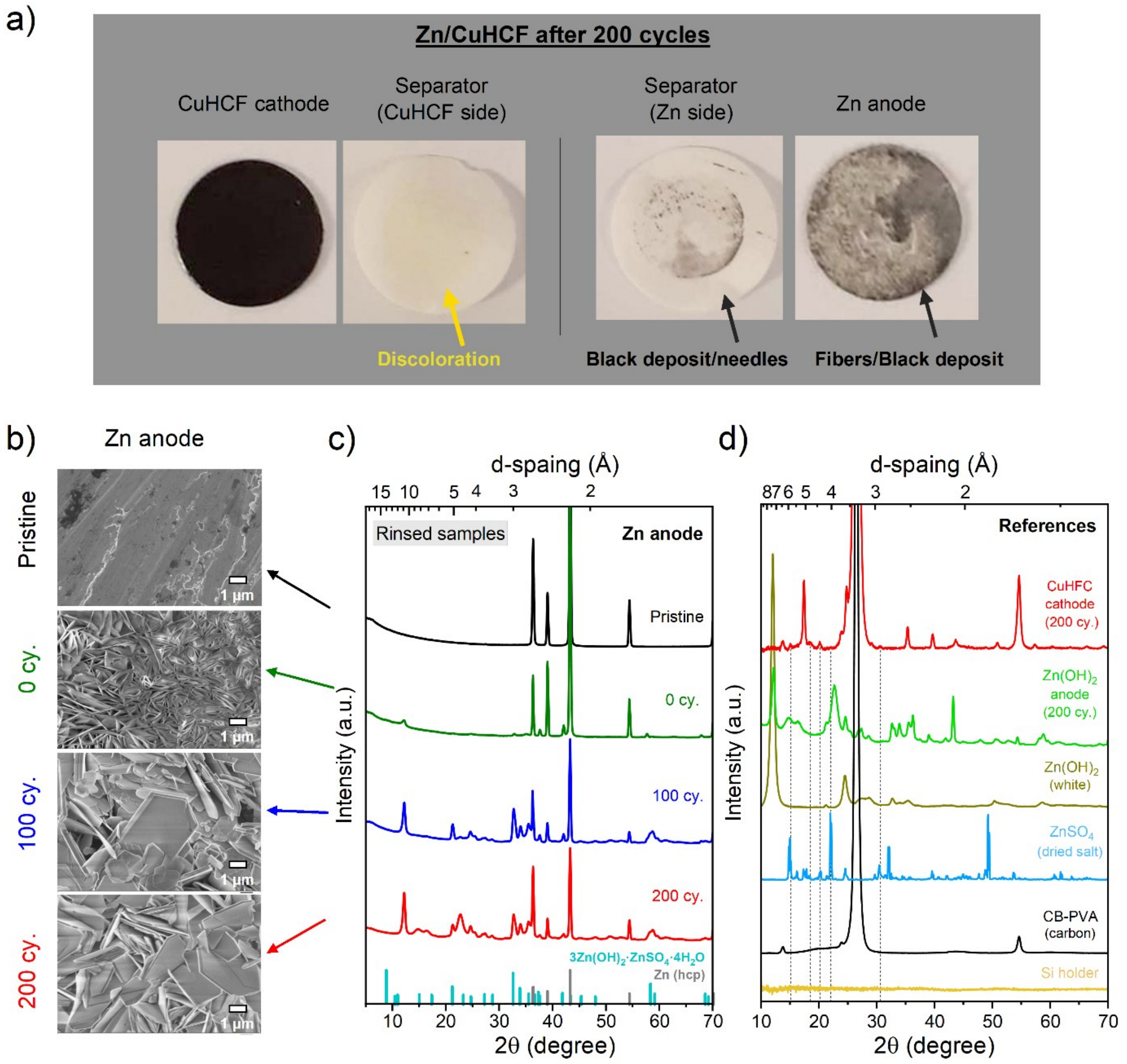

Figure S8. Analysis of the Zn/CuHCF cell with focus on the Zn anode. **(a)** Photographs of the Zn/CuHCF cell after 200 CV cycles between 1.00 V - 2.15 V in 1M $ZnSO_4$ at 2.5 mV s^{-1}. CuHCF cathode (left) and the Zn anode (right), and the two cellulose papers used as separators (shown next to its closest electrode). The darker deposits on the Zn anode are dendrites that have perforated the separator. On the CuHCF cathode, there are no visible changes except for cellulose fibers. However, the closest separator has a yellow discoloration. **(b)** SEM images of the Zn anode, from top to bottom: Pristine, 0 cycles (1 h at OCP), 100 cycles, and after 200 cycles. **(c)** The XRD patterns of the Zn anode at the respective stages of cycling. The new phase that appears with cycling matches a zinc hydroxide sulfate phase ($3Zn(OH)_2{\cdot}ZnSO_4{\cdot}4H_2O$). There are also additional reflections from the underlying metallic Zn phase (hcp). **(d)** XRD patterns of a few reference compounds. As can be clearly seen, the new emerging diffraction peaks in the CuHCF cathode (marked with dotted lines) do not match the above hydroxide-phase on the Zn anode. All cells were stopped at OCP and rinsed with ultrapure water prior to analysis.

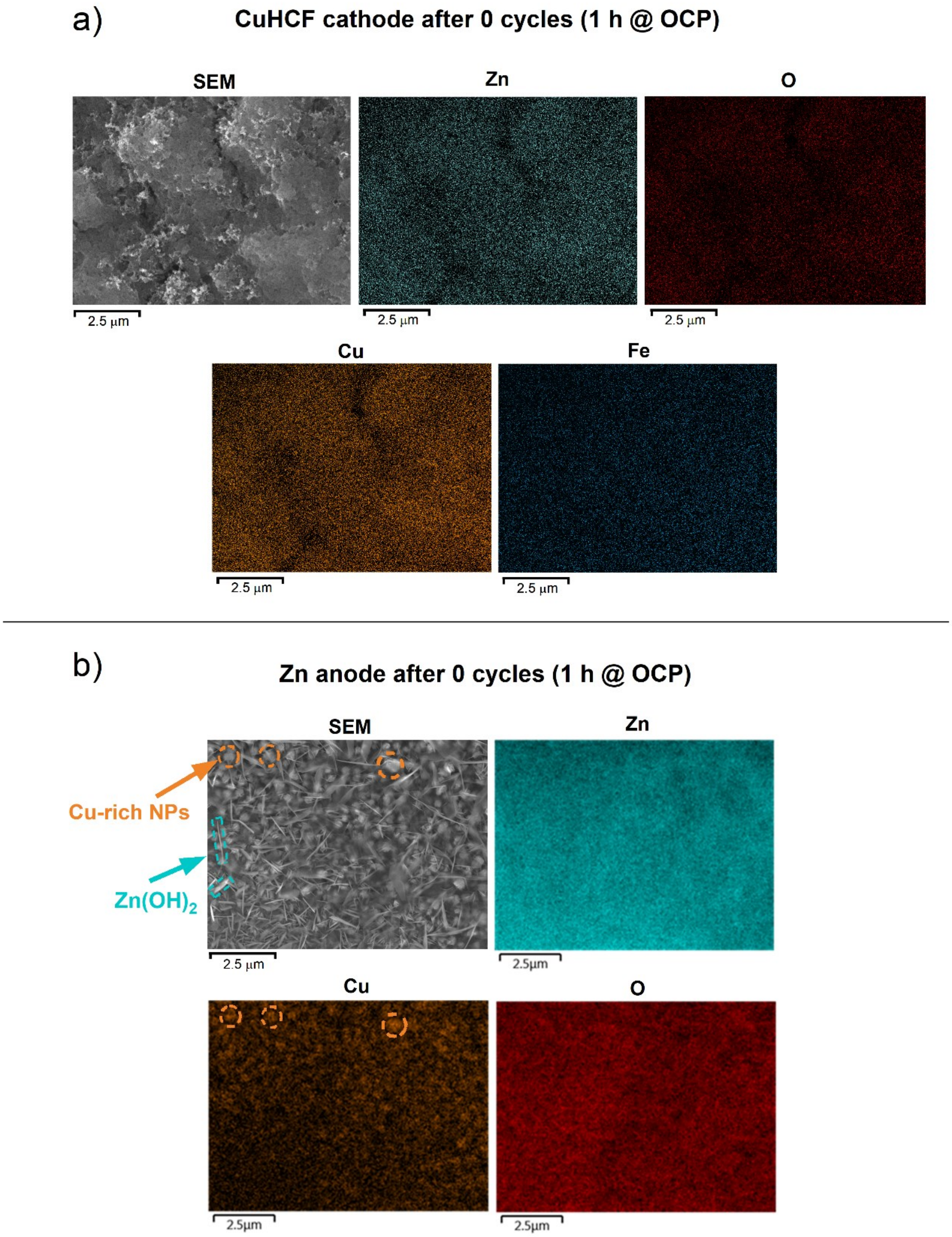

Figure S9. SEM-EDS elemental mapping of the Zn/CuHCF cell after 0 cycles (i.e., 1 h at OCP in 1 M $ZnSO_4$). **(a)** EDS maps of the CuHCF cathode. **(b)** EDS maps of the Zn anode. Both electrodes were rinsed with ultrapure prior to analysis to remove excess $ZnSO_4$ electrolyte. In the Zn anode, there were both hexagonal platelets (assigned to the $3Zn(OH)_2{\cdot}ZnSO_4{\cdot}4H_2O$ phase) and small Cu-rich nanoparticles scattered over the entire electrode. There were no visible changes for the CuHCF cathode. Some of the hexagonal platelets on the Zn anode have been marked with dotted green rectangles, and some Cu nanoparticles with orange circles to guide the eye.

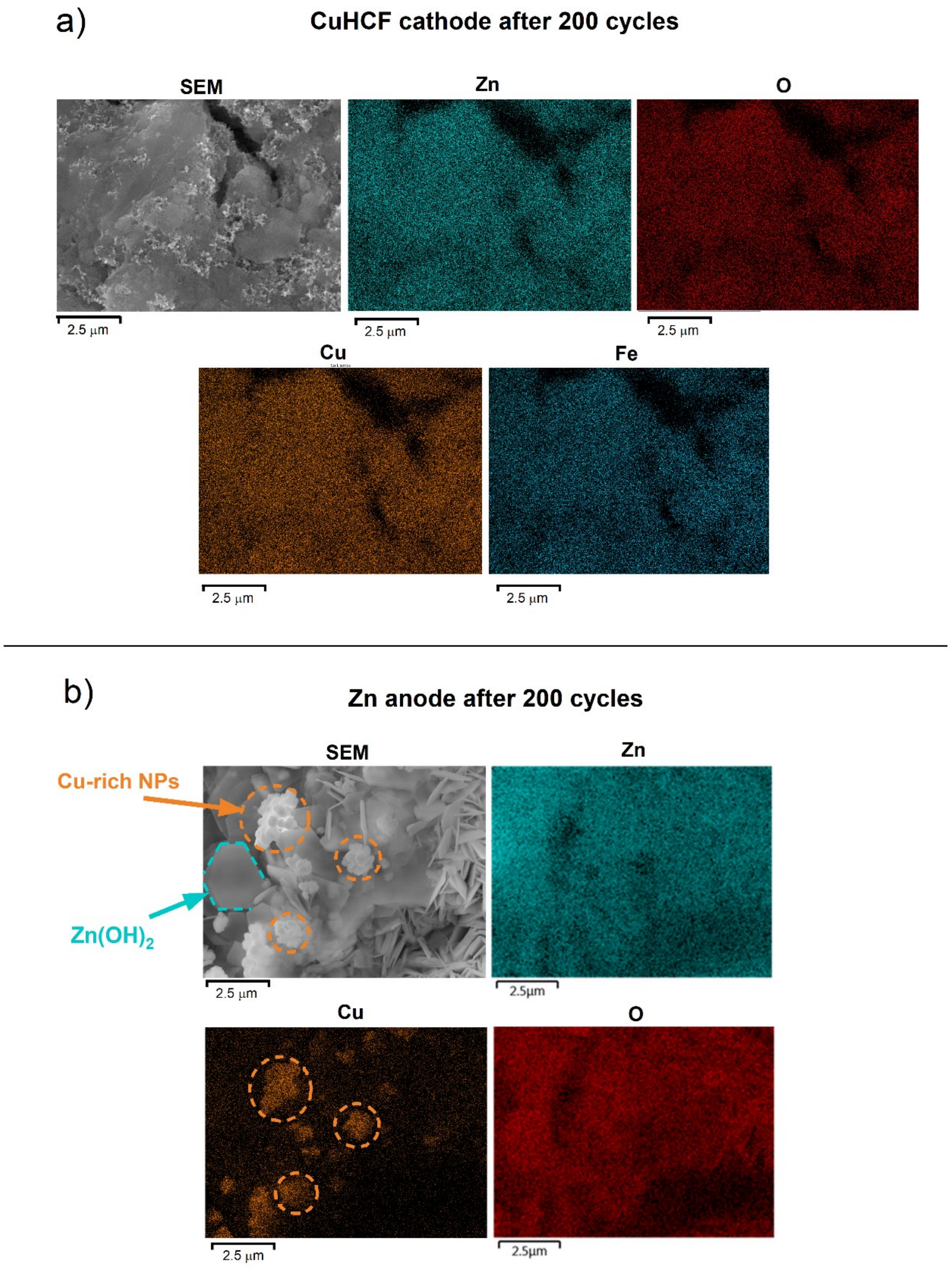

Figure S10. SEM-EDS elemental mapping of the Zn/CuHCF cell after 200 cycles between 1.00 - 2.15 V in 1M $ZnSO_4$ at 2.5 mV s^{-1}. **(a)** EDS maps of the CuHCF cathode. **(b)** EDS maps of the Zn anode. The samples were rinsed with ultrapure water prior to analysis to remove excess of $ZnSO_4$ electrolyte. No phase segregation is visible for the CuHCF cathode, however, both the hexagonal platelets and Cu-rich nanoparticles are visible on the Zn anode, similarly as seen after 0 cycles except that both the platelets and the particles appear larger in these samples. Some of the hexagonal platelets have been marked with dotted green hexagons, and the Cu nanoparticles with orange circles to guide the eye.

CuHCF cathode

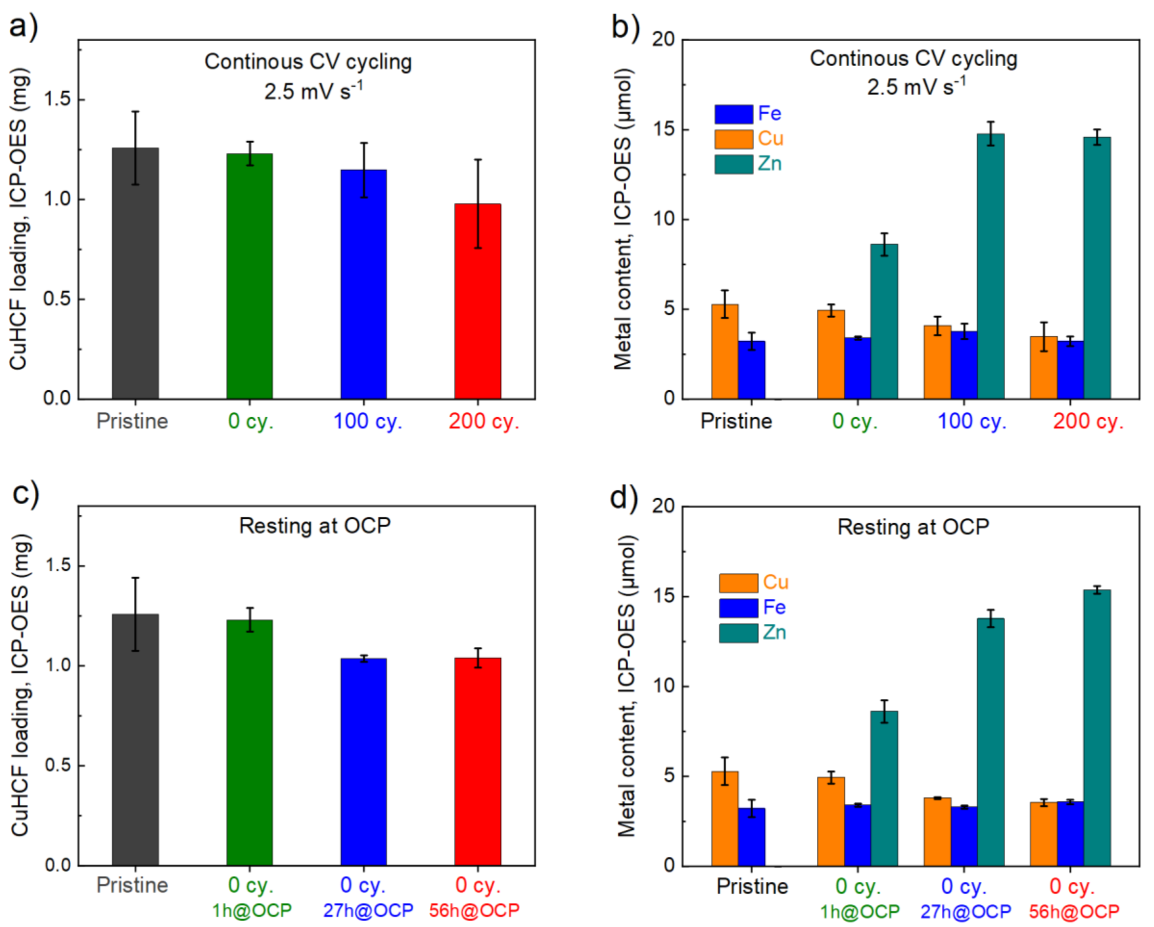

Figure S11. ICP-OES study of CuHCF cathode after either CV cycling (0 , 100, 200 cycles) or after resting at the cell OCP for different amount of time (0 h, 27 h, and 56 h) with 1M $ZnSO_4$ to investigate spontaneous aging processes. **(a)** Cathode loading of CuHCF after different stages of cycling: Pristine, 0 cycles, 100 cycles, and 200 cycles. **(b)** The corresponding metal content of Cu, Fe, and Zn. **(c)** Cathode loading of CuHCF after resting the cell at OCP: Pristine, 1 h, 27 h, and 56 h. These time intervals correspond to roughly the time it takes to cycle at the given conditions. **(d)** The corresponding metal content of Cu, Fe, and Zn after resting the cell at OCP. The loading of CuHCF in (a) and (c) was estimated assuming that Cu and Fe together constitute 41 wt. % of CuHCF, which was determined experimentally using ICP-OES of the pristine CuHCF powder without additions of PVA binder or carbon black. All electrochemical cycling was carried out between 1.00 – 2.15 V at 2.5 mV s^{-1} in 1 M $ZnSO_4$. The samples were washed with ultrapure water prior to analysis to remove excess of $ZnSO_4$ electrolyte. The error bars represent the standard error from repetitions of several electrodes.

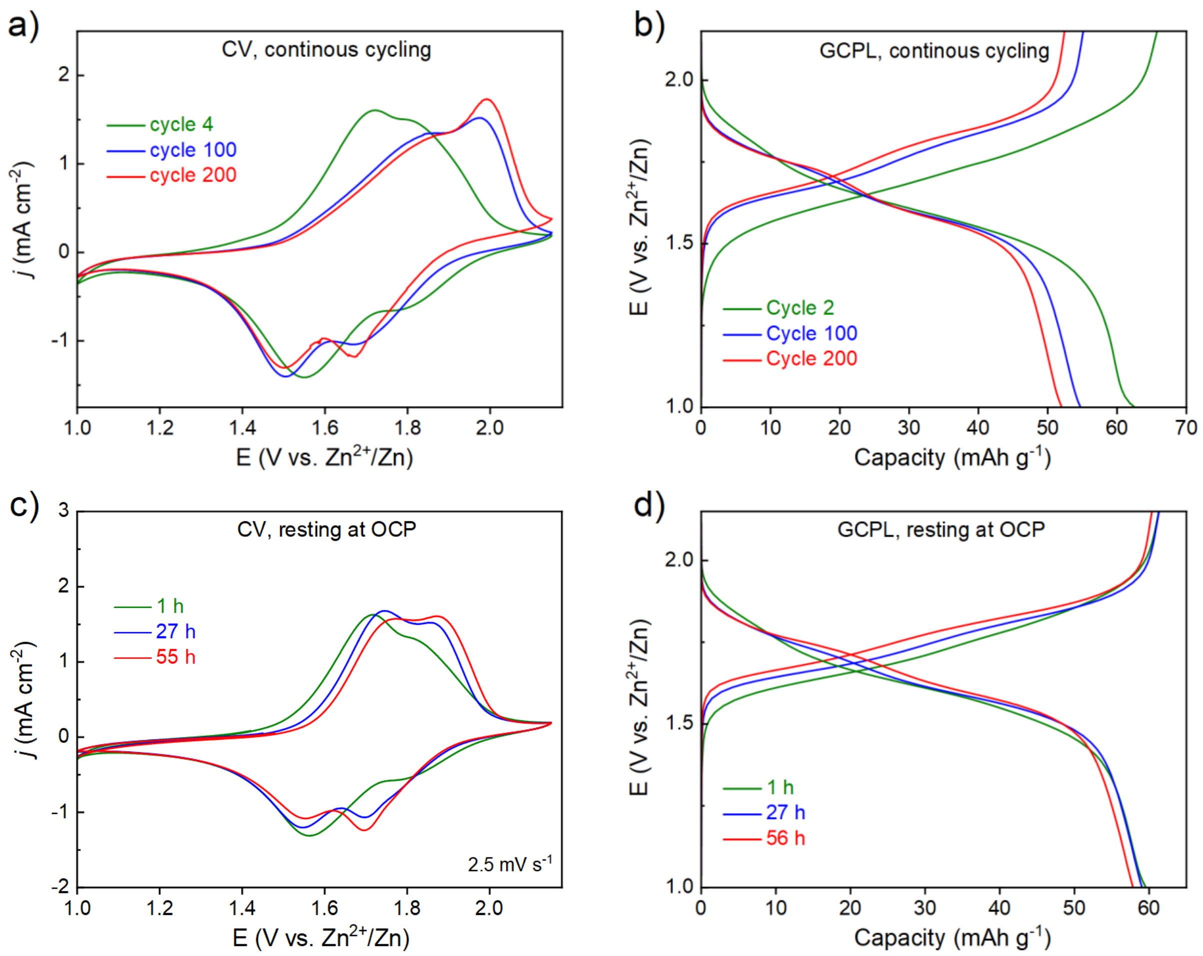

Figure S12. Aging characteristics of the CuHCF cathode after continuous cycling or resting at OCP. **(a)** The 2nd , 100th , and 200th cycles upon continuous CV cycling between 1.00 – 2.15 V at 2.5 mV s^{-1}. **(b)** The corresponding galvanostatic charge/discharge cycles upon continuous cycling at 8C **(c)** CV cycles recorded after resting the cell at OCP for 1 h, 27 h, and 56 h without cycling in between these time intervals. **(d)** The corresponding galvanostatic charge/discharge cycles recorded at 8C after resting at OCP. All experiments were carried out in 1 M $ZnSO_4$. All cycling were carried out at either 2.5 mV s^{-1} for CVs or at 8C for galvanostatic cycling (both cycling and resting).

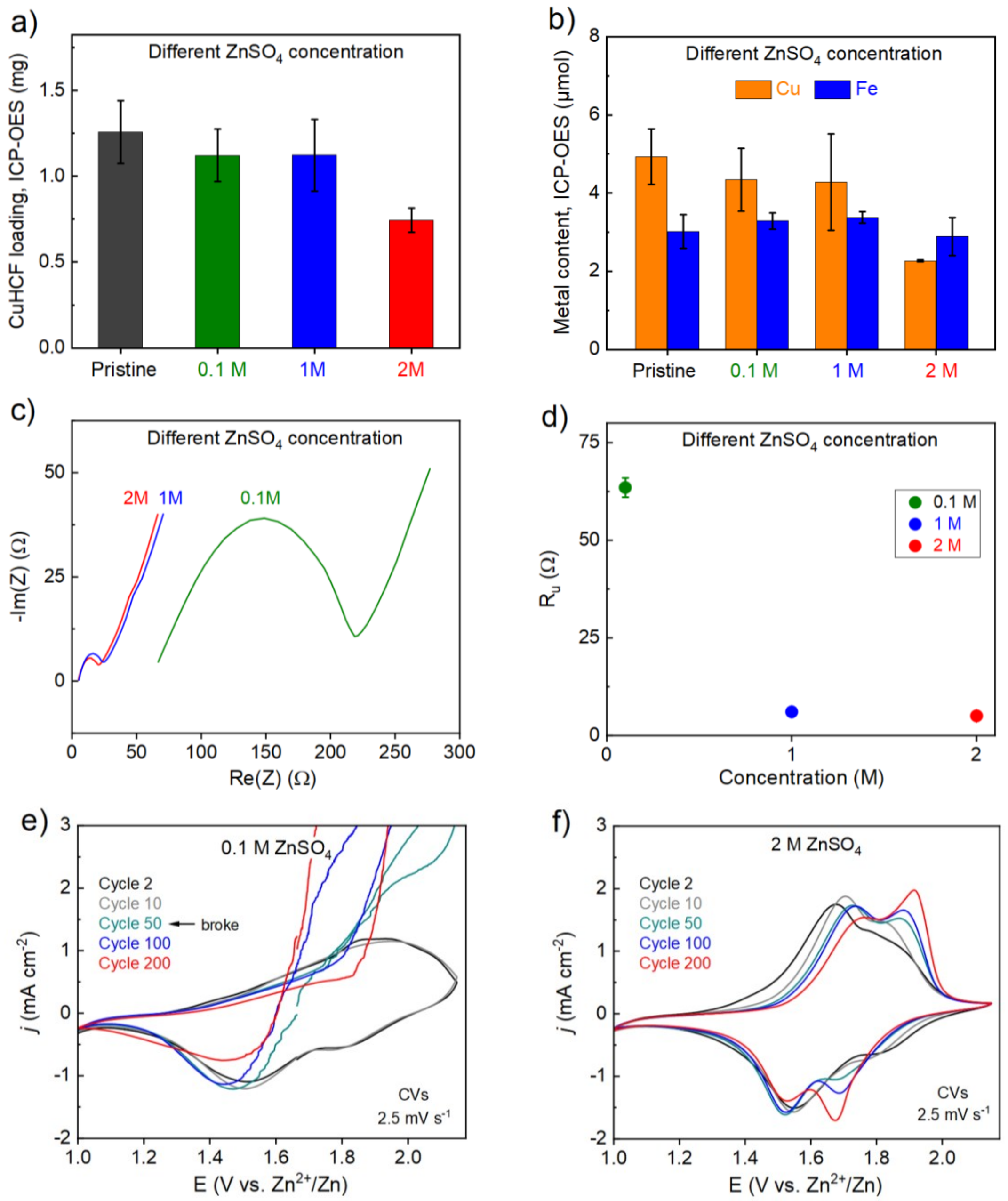

Figure S13. Impact of the $ZnSO_4$ electrolyte concentration (0.1 M, 1M, and 2 M) on the performance and stability of CuHCF cathode after 200 cycles between 1.00 – 2.15 V at 2.5 mV s^{-1}. **(a)** CuHCF loading from ICP-OES analysis after cycling in different electrolyte concentrations. **(b)** The corresponding metal content of Cu and Fe. **(c)** Electrochemical impedance spectroscopy (EIS) between 20 kHz-100 mHz **(d)** The extracted uncompensated solution resistance (R_u or iR-drop). **(e)** CVs in 0.1 M $ZnSO_4$. **(f)** CVs in 2 M $ZnSO_4$. All electrodes were rinsed with ultrapure water prior to ICP-OES analysis.

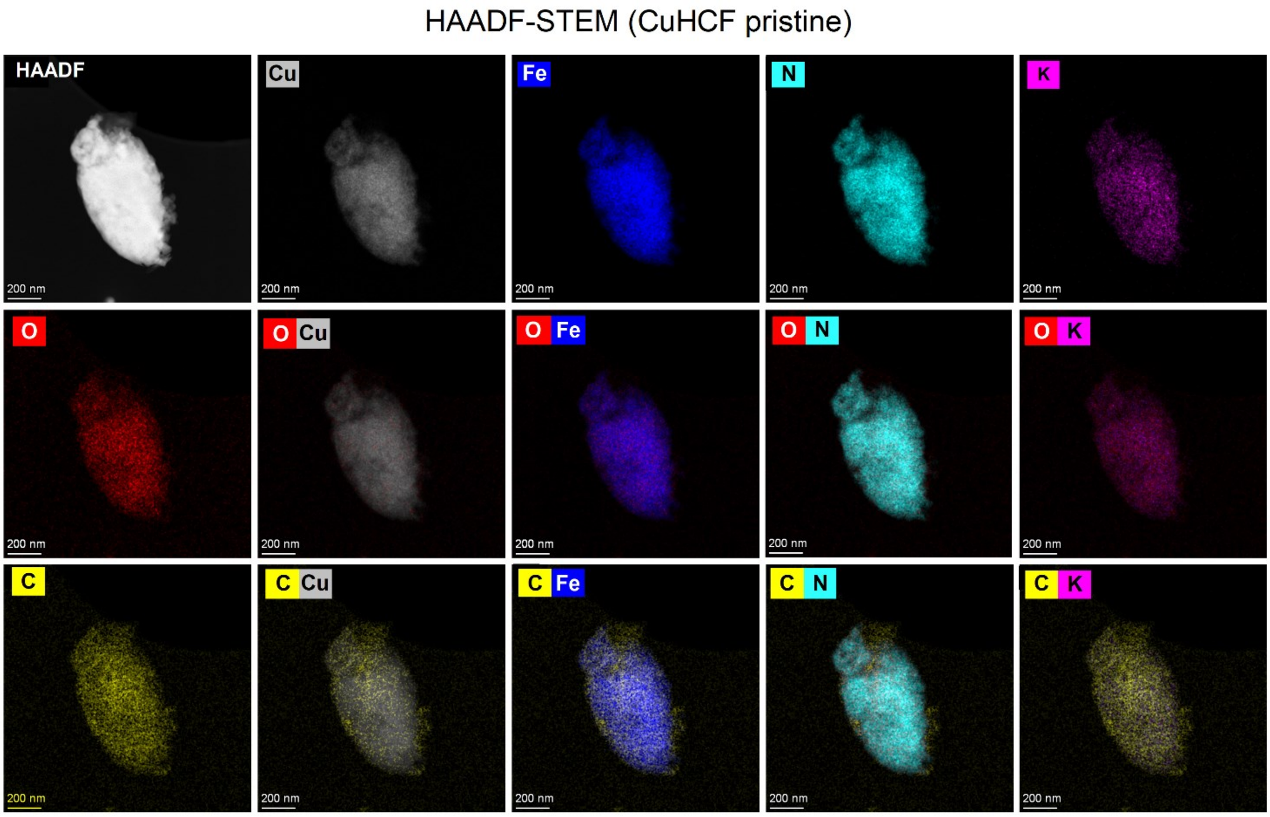

Figure S14. HAADF-STEM and EDS elemental maps of the pristine CuHCF cathode (consisting of 75 wt. % CuHCF, 15 wt. % PVA binder, and 10 wt. % carbon black). The HAADF image is shown in the upper left corner, the Cu, Fe, N, and K maps on the top row, and the O and C maps in the left column. The overlays are shown in the middle as composites of two elements. The scale bar is 200 nm. No signals of Zn and S were found in this sample. Note that we do not observe any phase segregation or surface enrichment of the O component in the pristine CuHCF cathode. There is although a segregation in the carbon signal, which is normal since the carbon black (CB) may not be homogenously distributed in the entire cathode cast.

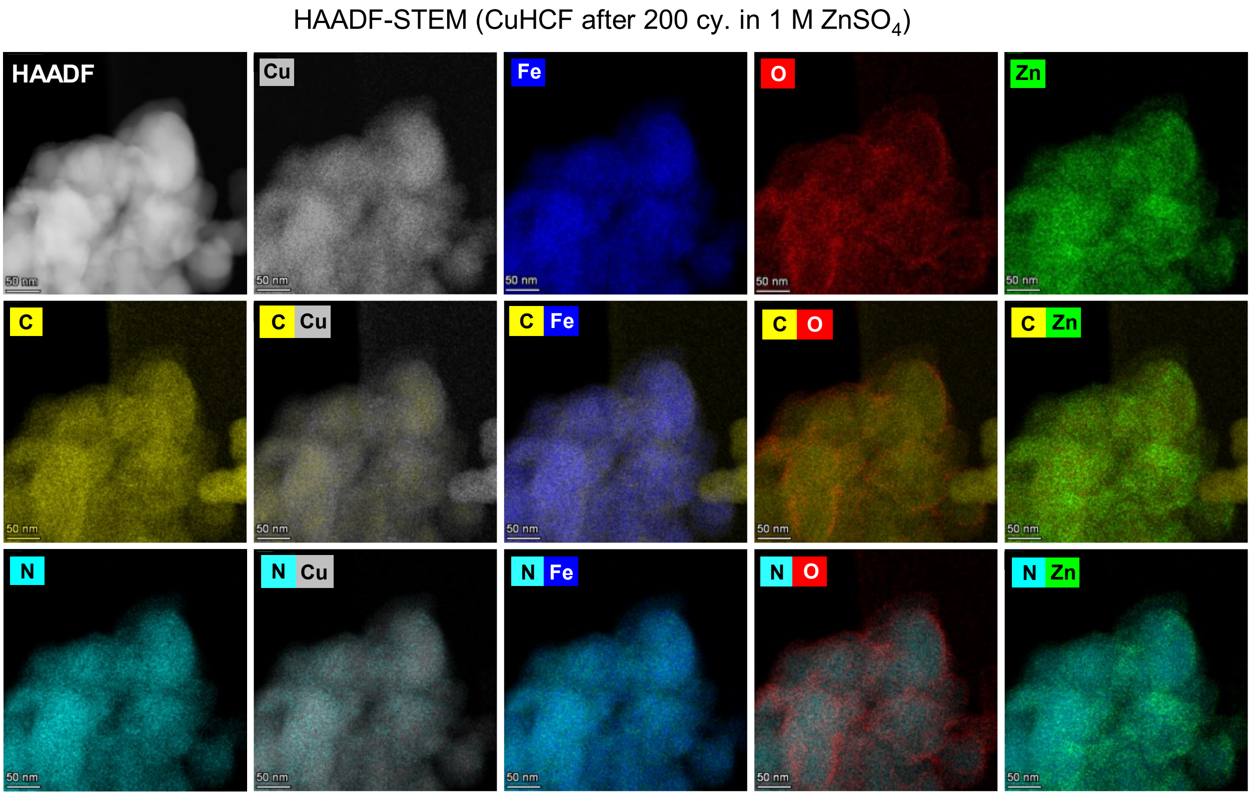

Figure S15. HAADF-STEM and EDS elemental maps of the CuHCF cathode (consisting of 75 wt. % CuHCF, 15 wt. % PVA binder, and 10 wt. % carbon black) after 200 cycles between 1.00 V - 2.15 V at 2.5 mV s^{-1} in 1M $ZnSO_4$, and stopped at OCP (~1.7 V *vs.* Zn^{2+}/Zn). The HAADF image is shown in the upper left corner, and the elemental maps of Cu, Fe, O, and Zn in the top row, and the C and N maps in the left column. The overlays are shown in the middle as composites of two elements. No signal of K was found in this sample. The samples were rinsed with ultrapure water before analysis to remove excess of $ZnSO_4$ electrolyte.

Table S3. Elemental compositions (in atomic %) from HAADF-STEM/EDS elemental mapping of the CuHCF cathode, both of the pristine CuHCF cathode cast and after 200 cycles between 1.00 V - 2.15 V at 2.5 mV s^{-1}, and stopped at OCP. The compositions were obtained from the *K*-lines. These samples were rinsed with ultrapure water before analysis. Note that the higher-than-expected Cu-signal originates from the TEM grid. The carbon content was omitted to allow comparison of the elements of interest, since the carbon content varies locally at this magnification. The standard error was estimated from repetitions of several particles.

Element	CuHCF pristine (at. %)	CuHCF after 200 cy. (at. %)
Cu	45.2 (1.6) (1)	26.2 (3.4) (1)
Fe	10.4 (2.5)	9.8 (0.1)
Zn	0.5 (0.3)	7.3 (0.4)
O	2.7 (4.8)	9.1 (0.6)
N	40.0 (8.9)	46.0 (2.5)
S	0.2 (0.4)	1.5 (0.1)
K	1.3 (0.1)	0.1 (0.1)
(1) The Cu-signal originates both from the CuHCF sample and the TEM grid.		

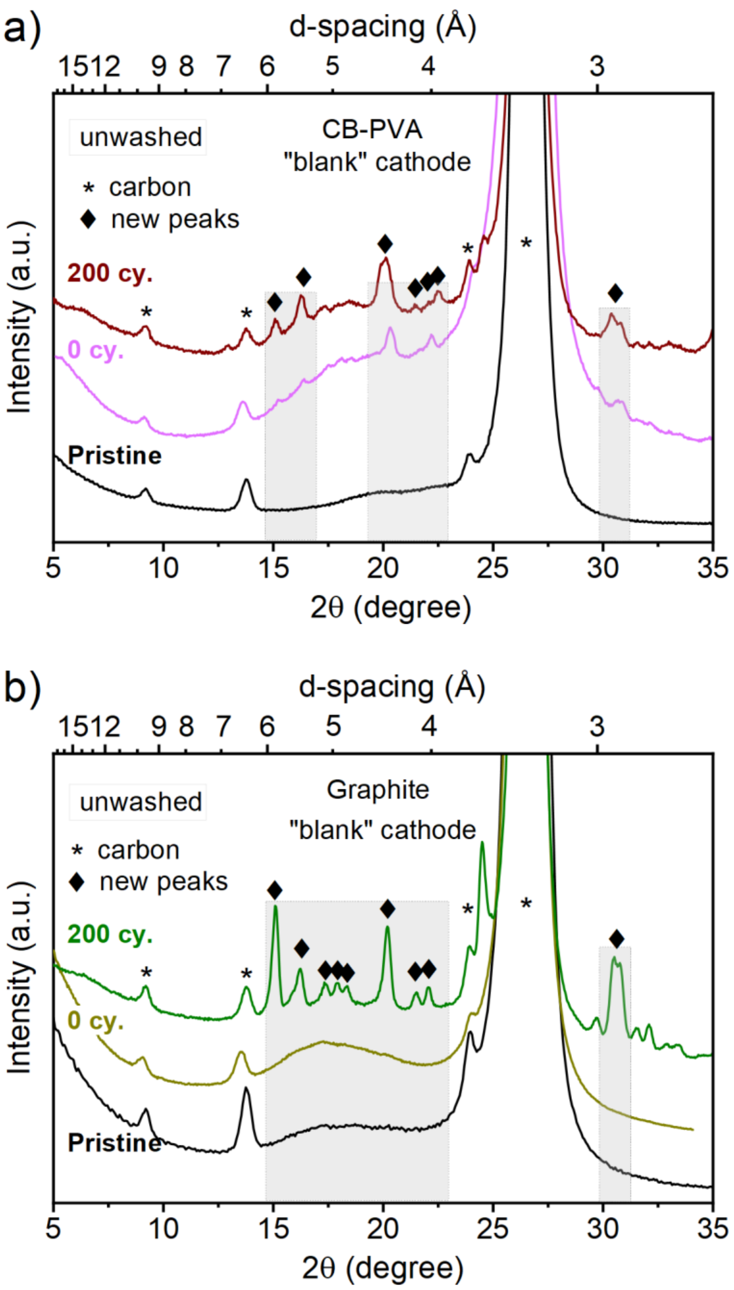

Figure S16. XRD of the cathodes in carbon "blank" cells (Zn/CB-PVA and Zn/graphite) after different stages of cycling; pristine, 0 cycles (i.e., 1 h at OCP), and after 200 cycles between 1.00 – 2.15 V at 2.5 mV s^{-1} in 1M $ZnSO_4$. **(a)** The CB-PVA cathode. **(b)** The graphite cathode. All electrodes were left unwashed since the diffraction peaks were much weaker than in their washed counterparts. The pristine electrodes were never exposed to electrolyte.

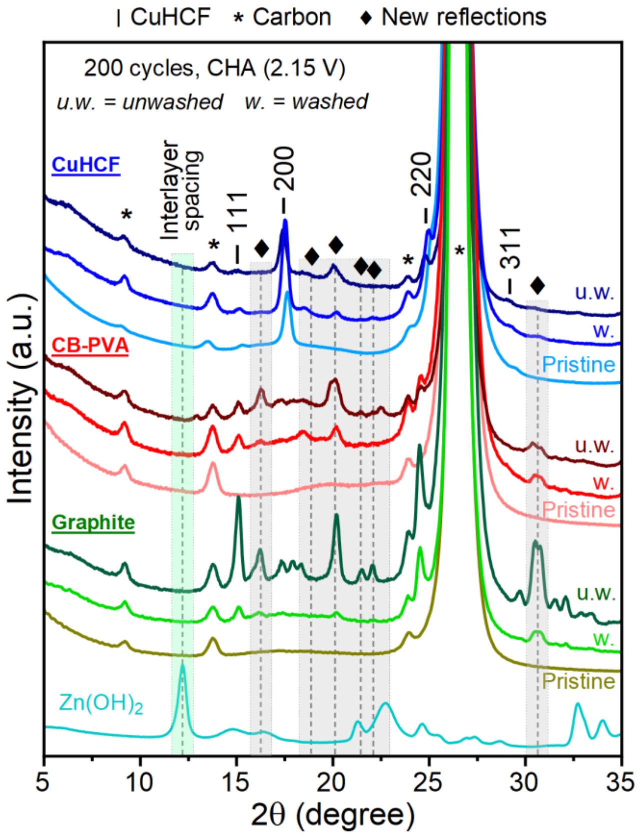

Figure S17. XRD of the cathodes of the carbon “blank” cells (Zn/CB-PVA or Zn/graphite), compared to the CuHCF cathode after 200 CV cycles between 1.00 – 2.15 V at 2.5 mV s^{-1} in 1M $ZnSO_4$. Shown samples are the pristine state and after 200 cycles and stopped in the charged state (CHA, 2.15 V), the latter where both washed (w.) and unwashed (u.w.) samples are compared in their charged state. Note that CB-PVA is used as conductive support in the cathode cast, and graphite foil as current collector. All these components are thus simultaneously present in the regular CuHCF cathode.

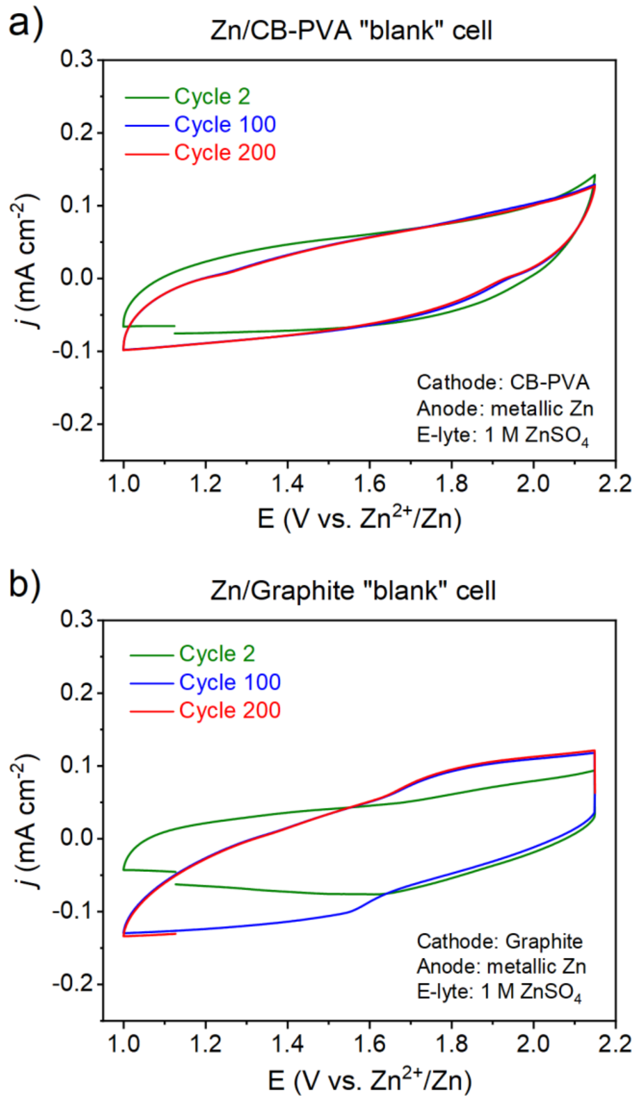

Figure S18. Cyclic voltammetry of the carbon blank cells at 2.5 mV s^{-1} in of 1M $ZnSO_4$. **(a)** The Zn/CB-PVA cell **(b)** The Zn/graphite cell. An inflection is visible in (b) on the anodic scan around ~1.7 V and on the cathodic scan around ~1.6 V, which may indicate an intercalation process.

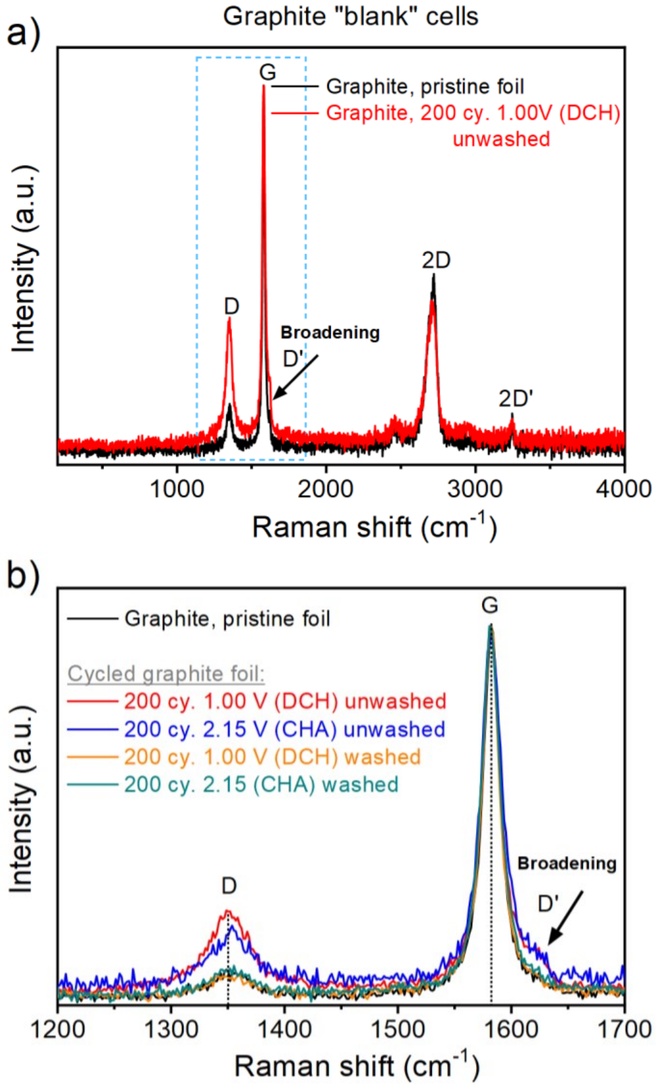

Figure S19. Raman spectra of the Zn/graphite cell. **(a)** Comparison of the spectra of the pristine graphite foil, and the unwashed graphite electrode after 200 CV cycles and stopped in the discharged state (1.00 V), shown in a wide region of 200-4000 cm^{-1}. **(b)** Enlargement of the spectral region between 1200-1700 cm^{-1} to visualize the characteristic D band (A_{1g} mode) and the G band (E_{2g} mode). The spectra show the pristine graphite foil, and the graphite electrodes after 200 cycles and stopped in either the discharged (1.00 V) or the charged (2.15 V) states. Both unwashed and washed samples are shown. Electrochemical cycling was carried out between 1.00 – 2.15 V vs. Zn^{2+}/Zn at a scan-rate of 2.5 mV s^{-1} in 1M $ZnSO_4$. Note that major changes are visible for the cycled graphite electrodes in the D band region (~1350 cm^{-1}) and in the D’ feature (1620 cm^{-1}), where a slight shift and broadening of the overtone (2D band) is visible with respect to the spectrum of pristine graphite foil. The rise of the D band for the cycled graphite electrodes is associated with an increase in the local disorder. Ideally, in perfect graphite, the A_{1g} mode (D band) is forbidden and is only activated in presence of disorder.[13] This circumstance is confirmed by the appearance/broadening of the D’ feature next to the main G band. The D’ feature is characteristic of defected graphite, and the slight broadening of the 2D band at higher wavenumbers shown in (a) points to higher disorder and possible local distortions of the graphitic structure. In all the cycled graphite electrodes, the spectral features associated with the disorder disappear almost completely after rinsing the electrodes with water. This suggests that the contact with 1M $ZnSO_4$ electrolyte and cycling induces a mild intercalation of Zn^{2+} and/or SO_4^{2-} into the graphite structure. As a result, part of the electrolyte enters the surface structure of the graphite, nevertheless, after washing with ultrapure water the salt is removed from the surface whereby the original spectral features of pristine graphite are resumed.

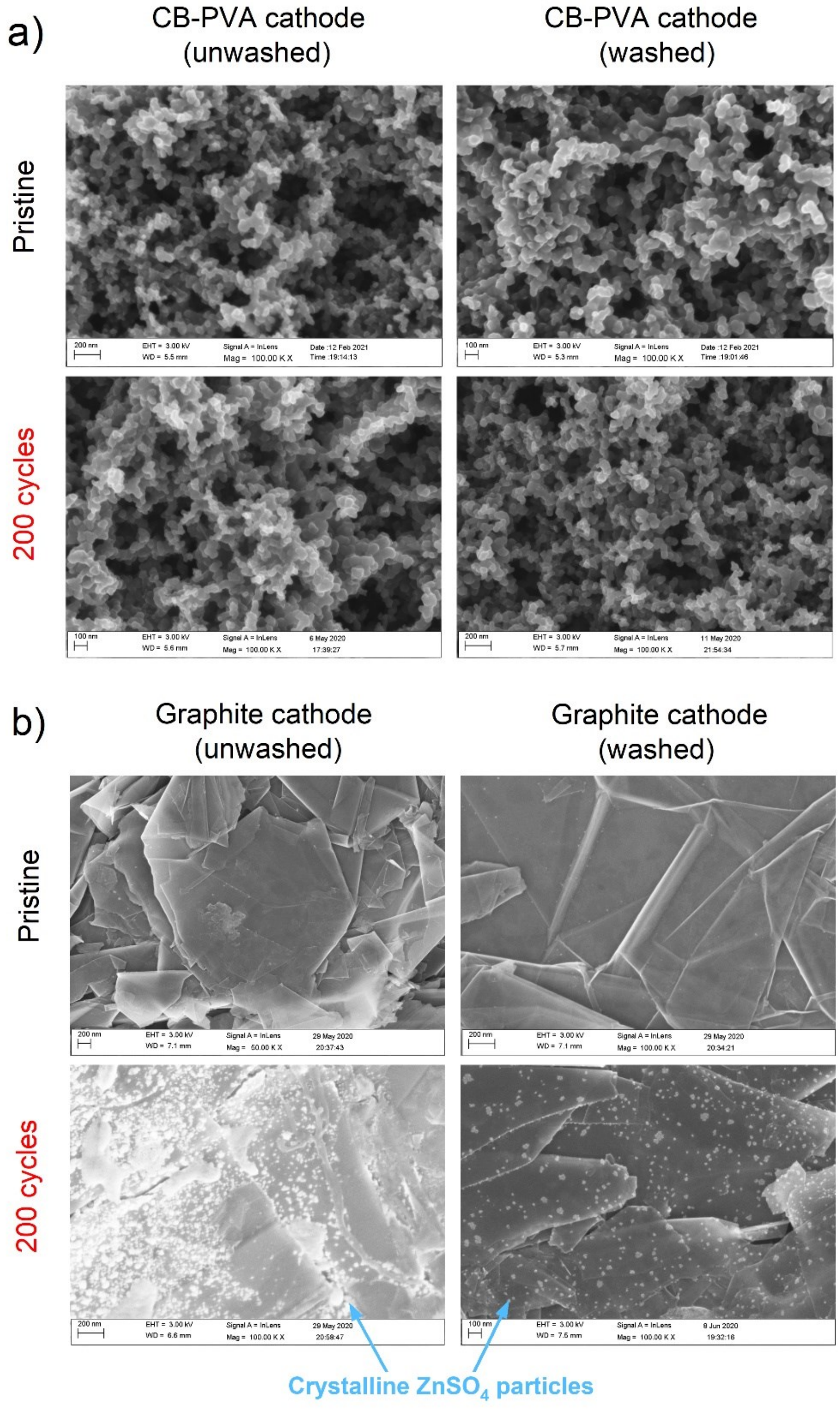

Figure S20. SEM images of the washed and unwashed cathodes of the carbon blank cells (Zn/CB-PVA and Zn/graphite) before and after CV cycling between 1.00 - 2.15 V at 2.5 mV s^{-1} in 1M $ZnSO_4$, and stopped at OCP. **(a)** The unwashed CB-PVA electrode (left) and the CB-PVA electrode after washing with ultrapure water (right). **(b)** The unwashed graphite electrode (left) and the washed graphite electrode (right). Small "white" particles (indicating electric charging) appear on the graphite electrode after 200 cycles, which are more pronounced in the unwashed graphite. Many of these particles can be removed by washing with water, although, there are still some particles left. No particles are visible in the CB-PVA electrode, although, they may be more challenging to detect due to the high surface area of the carbon black particles compared to graphite foil.

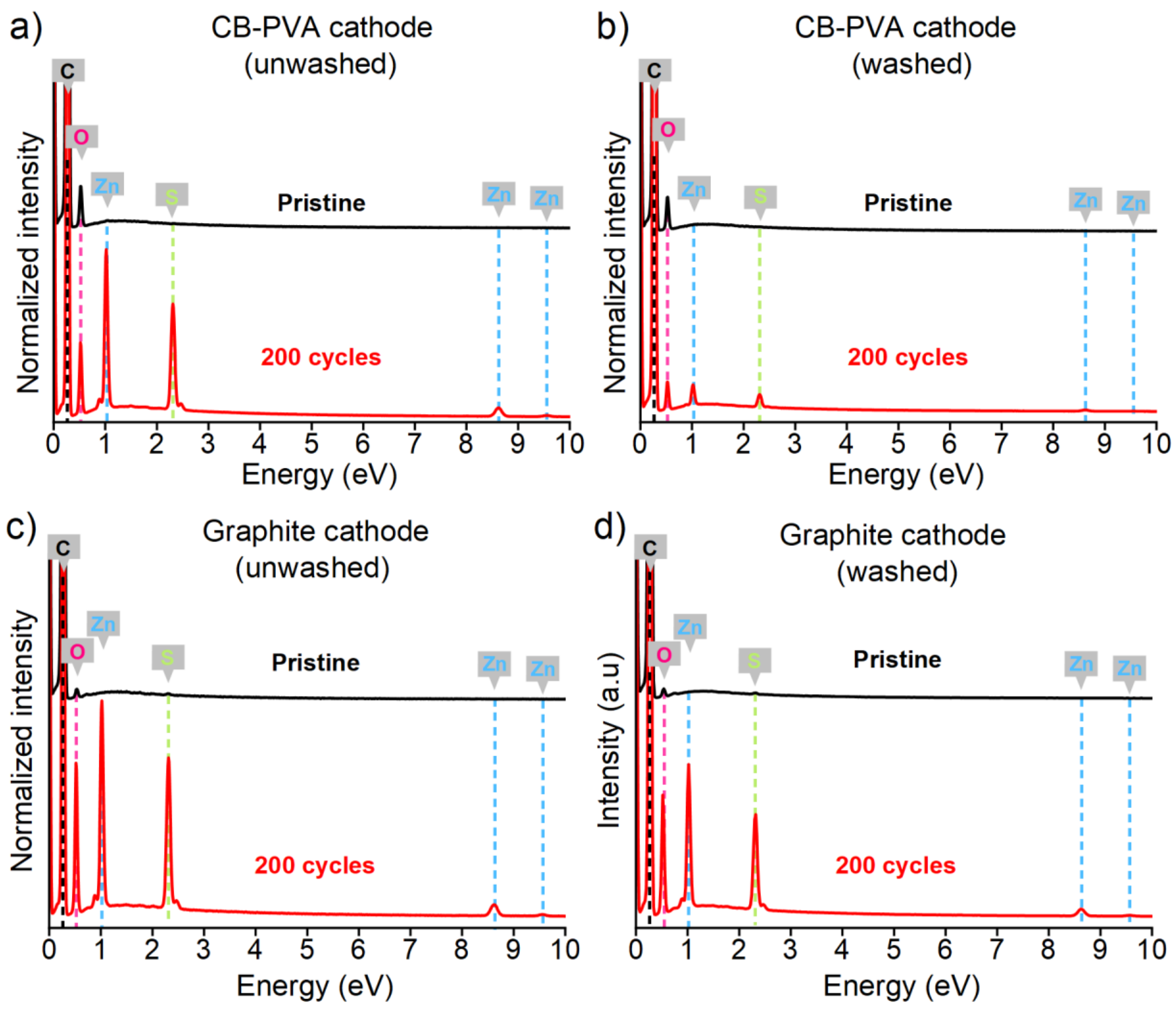

Figure S21. EDS analysis of the cathodes in the carbon "blank" cells (Zn/CB-PVA and Zn/graphite) in the pristine state and after 200 cycles between 1.00 and 2.15 V *vs.* Zn^{2+}/Zn at 2.5 mV s^{-1} in 1M $ZnSO_4$, and stopped at OCP. **(a)** The unwashed CB-PVA cathode. **(b)** The CB-PVA cathode washed with ultrapure water to remove excess of $ZnSO_4$ salt. **(c)** The unwashed graphite cathode. **(d)** The washed graphite cathode. All EDS spectra were normalized to the total unit area, and have the same y-axis scale to allow for direct comparison between the samples. It is visible that the Zn, S and O signals decrease after washing the cycled electrodes with water, although, a large extent remains in graphite.

Table S4. Elemental compositions determined by SEM-EDS of the cathodes of two carbon "blank" cells (Zn/CB-PVA and Zn/graphite). The pristine state and the electrodes after 200 CV cycles between 1.00 - 2.15 V at 2.5 mV s^{-1} in 1M $ZnSO_4$ are shown. The standard errors given in brackets were estimated from repetitions of several electrodes. Note that there are minor impurities of sulfur (S) in the pristine graphite, which may originate from the manufacturing process. The compositions are shown in atomic %.

**Elemental compositions from EDS (at. %)**					
**CB-PVA cathode**	**Washing procedure**	**C**	**O**	**Zn**	**S**
Pristine	unwashed	94.9 (1.7)	5.1 (1.7)	n/a	n/a
Pristine	washed	94.2 (1.5)	4.4 (1.5)	n/a	n/a
200 cycles	unwashed	89.4 (0.2)	6.8 (0.6)	1.9 (0.2)	1.8 (0.3)
200 cycles	washed	97.3 (0.8)	2.5 (0.6)	0.09 (0.02)	0.03 (0.01)
**Graphite cathode**	**Washing procedure**	**C**	**O**	**Zn**	**S**
Pristine	unwashed	99.5 (0.1)	0.50 (0.05)	n/a	0.03 (0.01)
Pristine	washed	99.6 (0.1)	0.28 (0.03)	n/a	0.03 (0.01)
200 cycles	unwashed	82.0 (3.6)	13.2 (3.1)	2.4 (0.3)	2.4 (0.2)
200 cycles	washed	86.0 (1.1)	10.7 (0.8)	1.7 (0.2)	1.6 (0.2)
[n/a] The element was not detected in the sample					

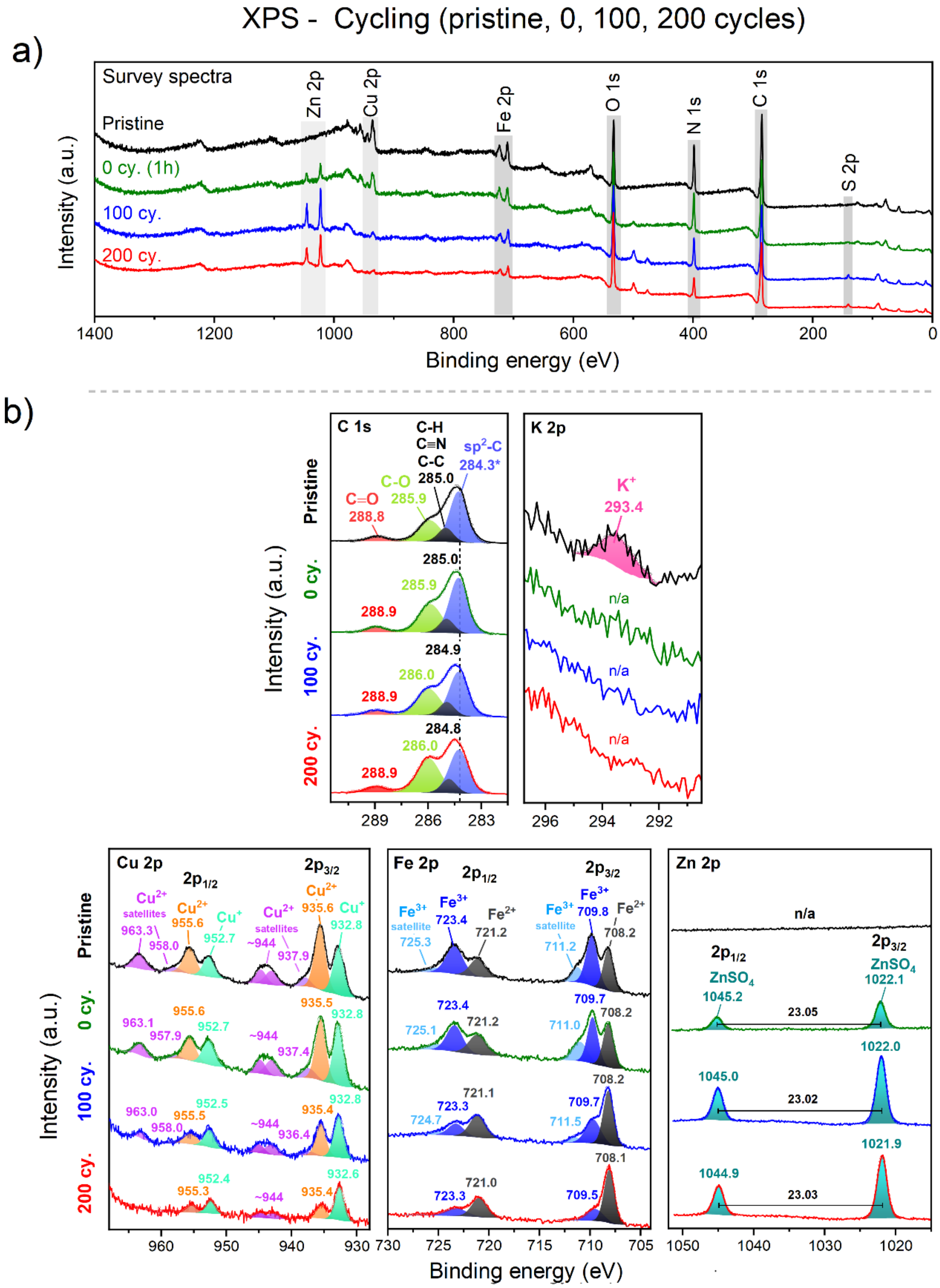

Figure S22. XPS spectra of the CuHCF cathode after different stages of cycling between 1.00 - 2.15 V at 2.5 mV s^{-1} in 1M $ZnSO_4$. **(a)** Survey spectra. **(b)** High-resolution spectra (C 1s, K 2p, Cu 2p, Fe 2p, and Zn 2p). The cycled states are displayed from top to bottom: Pristine, 0 cycles (1h at OCP), 100 cycles, and 200 cycles. All spectra were energy calibrated to the graphitic sp^2-C peak (284.3 eV) from the carbon black. The Cu 2p, Fe 2p, and Zn 2p spectra are also shown in the main manuscript, however, here, the entire spectra are displayed including the $2p_{1/2}$ and $2p_{3/2}$ regions.

Table S5. Compositions from XPS of the CuHCF cathode after different stages of cycling between 1.00 V – 2.15 V in 1M $ZnSO_4$. The compositions are reported as at. %.

Element/XPS peak	Pristine (at. %)	0 cycles 1 h@ OCP (at. %)	100 cycles (at. %)	200 cycles (at. %)
Cu $2p_{3/2}$	1.4	1.2	0.4	0.2
Fe $2p_{3/2}$	0.9	0.8	0.7	0.5
Zn $2p_{3/2}$	n/a	0.4	1.3	1.0
O 1s	15.6	16.6	18.8	21.0
N 1s	15.3	14.7	11.2	9.0
S 2p	n/a	0.25	0.30	0.2
C 1s	67.6	66.8	67.5	68.0
K 2p	0.10	0.06	n/a	n/a

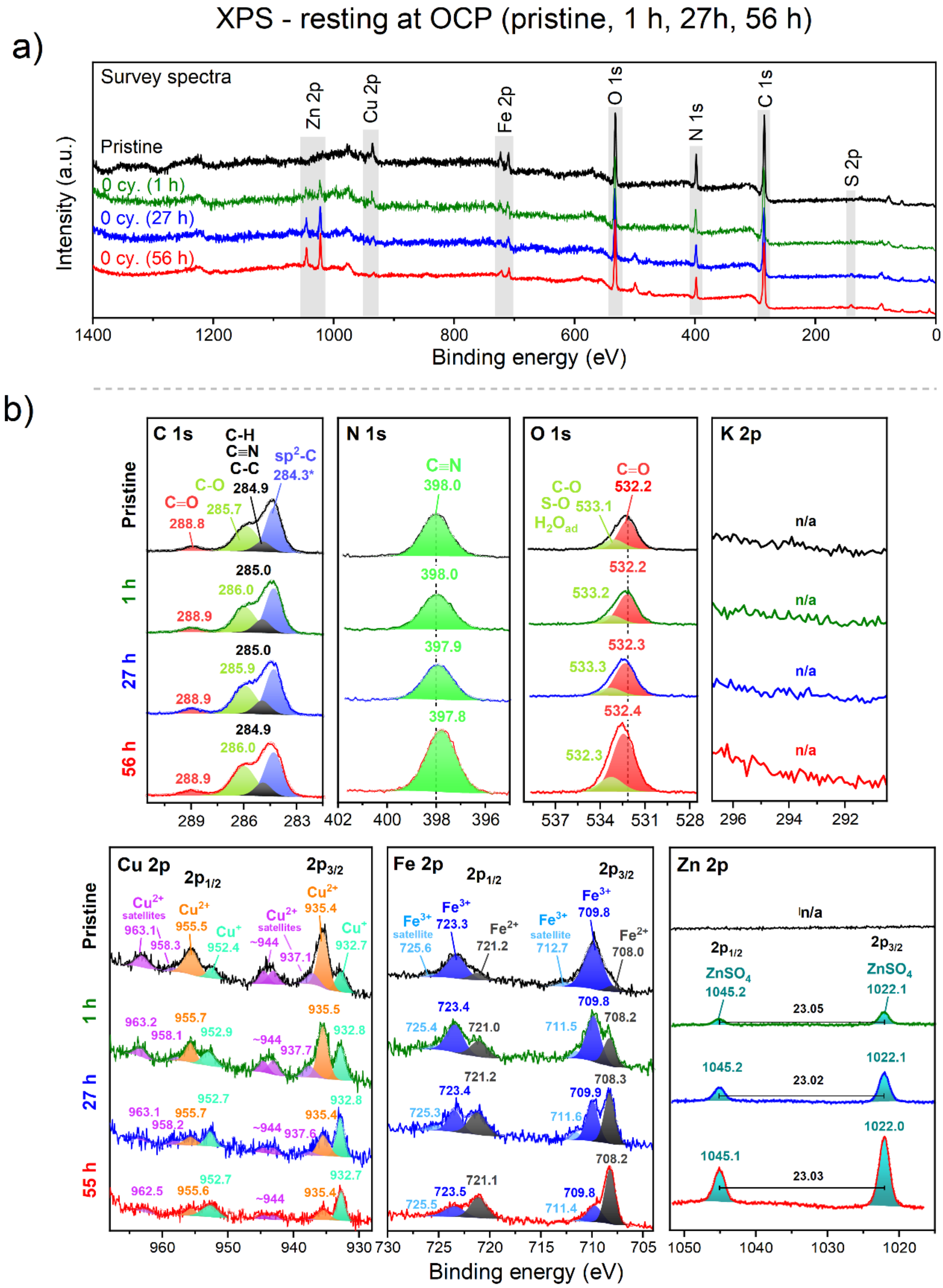

Figure S23. XPS spectra of the CuHCF cathode after resting at OCP for different times to investigate spontaneous aging. Instead of cycling, the Zn/CuHCF cell was left to rest at OCP for 1 h, 27 h, and 56 h (i.e., for approximately the same amount of time it takes to cycle 0, 100, and 200 cycles). Note that OCP imposes a cell voltage of ~1.7 V vs. Zn^{2+}/Zn. **(a)** Survey spectra. **(b)** High-resolution spectra (C 1s, K 2p, Cu 2p, Fe 2p, and Zn 2p). The aged states are displayed from top to bottom: Pristine, 1 h, 27 h, and 56 h at OCP. All spectra were energy calibrated to the graphitic sp^2-C peak of the carbon black additive (284.3 eV), and washed with ultrapure water to remove excess of $ZnSO_4$ electrolyte.

Table S6. Compositions from XPS of the CuHCF cathode after resting the Zn/CuHCF cell at OCP (i.e., spontaneous aging) in 1M $ZnSO_4$. The Zn/CuHCF cell was left for 1 h, 27 h, and 56 h. The compositions are reported as atomic %, and all samples were washed with ultrapure water prior to analysis to remove excess of $ZnSO_4$.

**XPS peak**	**Pristine (at. %)**	**0 cycles 1 h @ OCP (at. %)**	**0 cycles 27 h @ OCP (at. %)**	**0 cycles 56 h @ OCP (at. %)**
Cu $2p_{3/2}$	0.9	0.6	0.3	0.2
Fe $2p_{3/2}$	0.6	0.5	0.6	0.5
Zn $2p_{3/2}$	n/a	0.5	0.9	1.1
O 1s	15.1	18.1	19.5	18.1
N 1s	8.9	9.3	8.8	7.5
C 1s	74.4	70.8	69.9	72.7
K 2p	0.18	n/a	n/a	n/a

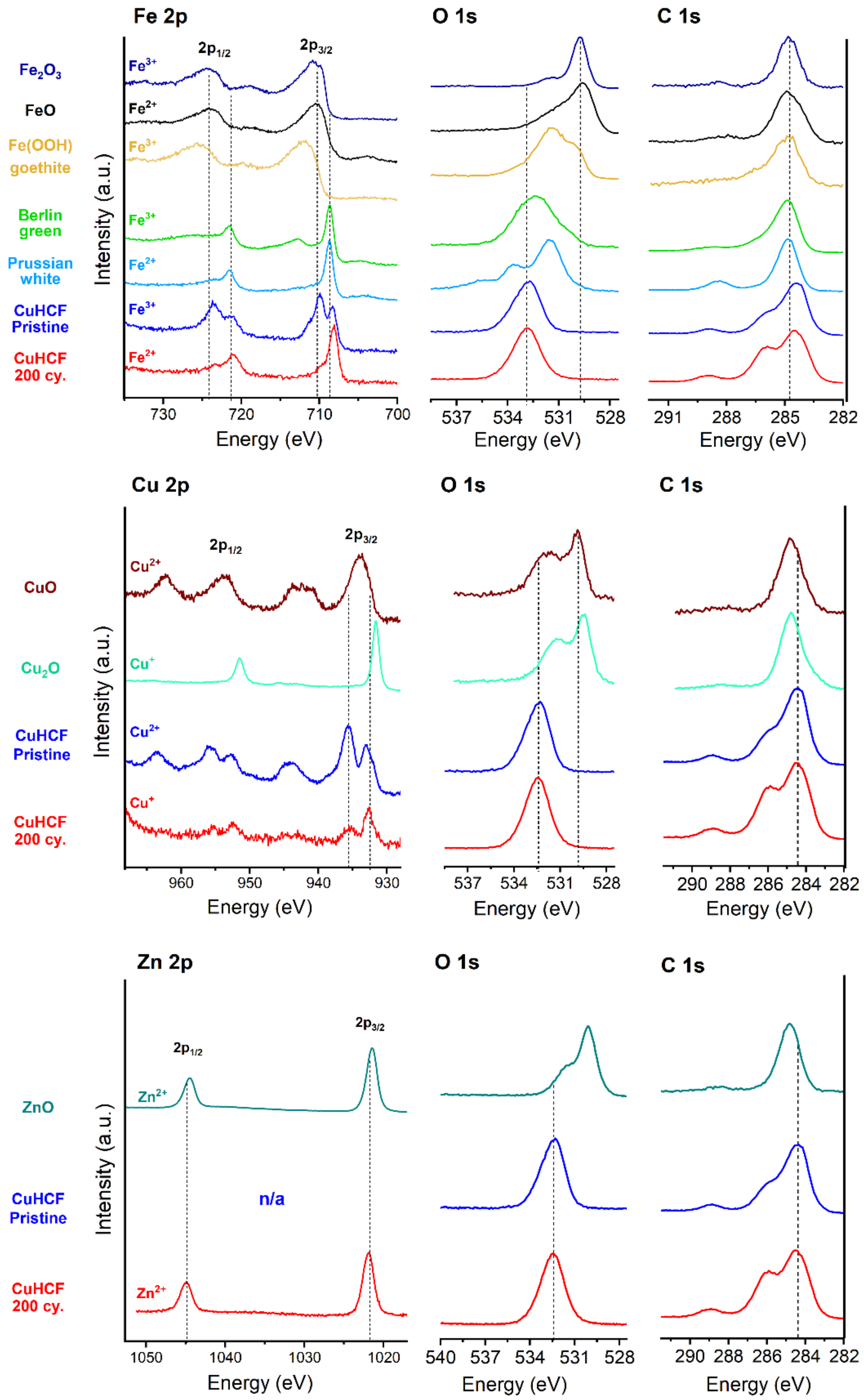

Figure S24. XPS of reference compounds. All samples were calibrated by setting the sp^3-C peak (adventitious carbon) to 284.8 eV, except for the two CuHCF cathode samples shown at the bottom, which were calibrated to the sp^2-C peak at 284.3 eV, since these contain graphitic carbon.

Table S7. Edge positions and oxidation states from *K*-edge XAS. The edge positions were obtained at half-height of the normalized absorption jump, i.e., at 0.5 absorption units.

**Cu *K*-edge**	**Experimental Setup**	**Edge position (eV)**	**Oxidation state**
Pristine	ex situ	8989.3	2 [(a)]
0 cycles	ex situ	8989.2	2 [(a)]
100 cycles	ex situ	8989.1	2 [(a)]
200 cycles	ex situ	8989.1	2 [(a)]
2.15 V (CHA)	in situ	8989.0	2 [(b)]
1.00 V (DCH)	in situ	8987.4	(2-σ) [(b)]
**Fe *K*-edge**		**Edge position (eV)**	**Oxidation state**
Pristine	ex situ	7126.3	3.0 [(c)]
0 cycles	ex situ	7126.4	3.2 [(c)]
100 cycles	ex situ	7125.9	2.4 [(c)]
200 cycles	ex situ	7125.9	2.4 [(c)]
2.15 V (CHA)	in situ	7126.2	3.0 [(c)]
1.00 V (DCH)	in situ	7125.6	2.0 [(c)]
**Zn *K*-edge**		**Edge position (eV)**	**Oxidation state**
Pristine	ex situ	-	-
0 cycles	ex situ	9662.2	2
100 cycles	ex situ	9662.1	2
200 cycles	ex situ	9662.0	2
2.15 V (CHA)	in situ	9659.7	2 [(d)]
1.00 V (DCH)	in situ	9659.6	2 [(d)]

(a) The oxidation state of Cu estimated from the positions of the peaks assigned to Cu^{2+} and Cu^{+}.
(b) The oxidation state of the in situ samples contained a fraction of Cu^{+} species, however, the absolute amount was not estimated.
(c) The oxidation state of Fe was determined from the edge position for low-spin Fe compounds.
(d) The Zn *K*-edge position is different between the ex situ and in situ configurations, which can be explained by the sensitivity of the edge position to the ligand and coordination geometry. We judge that Zn is always in oxidation state +2 in this work, and that the change in the XANES region reflects a change in the coordination environment.

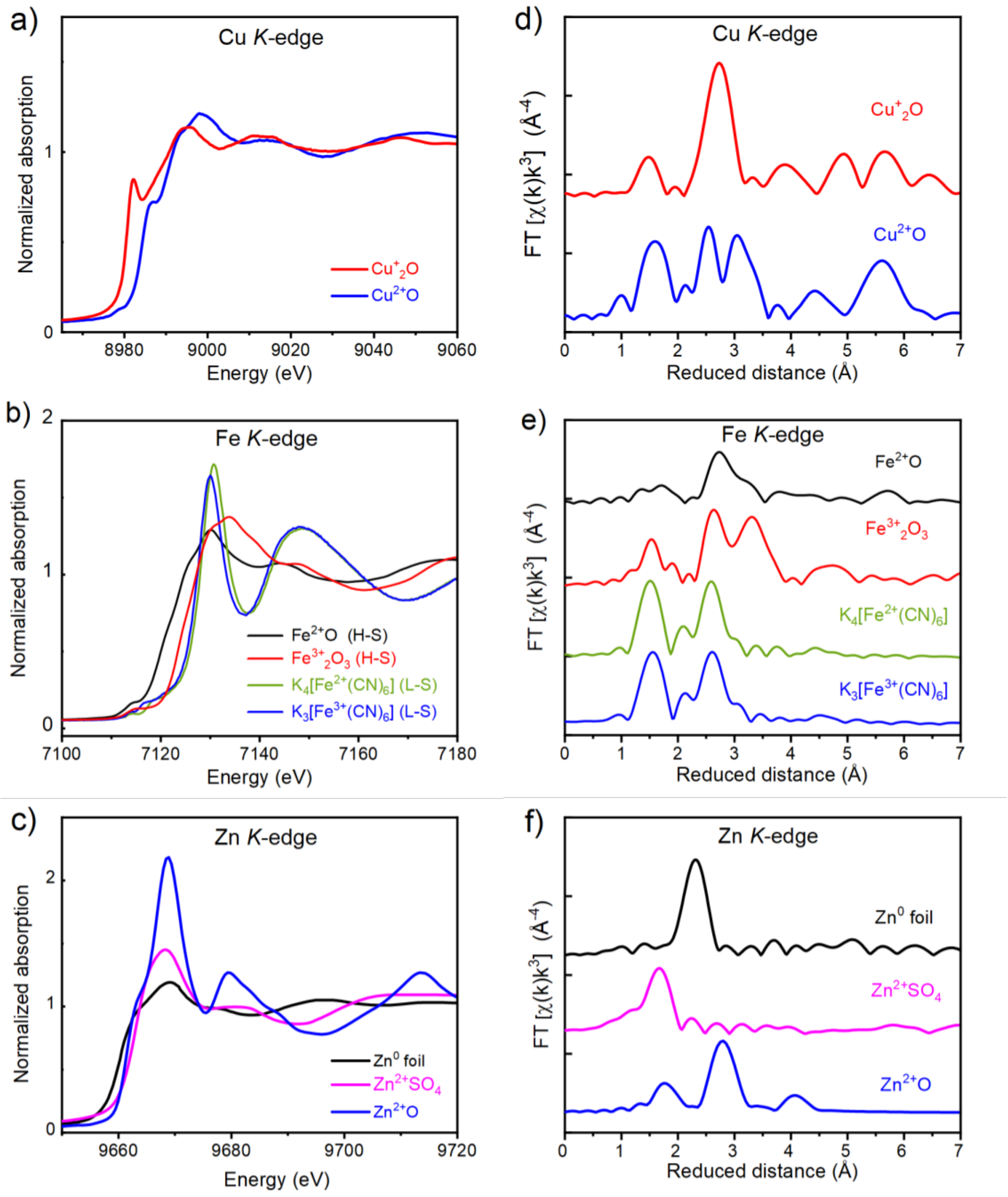

Figure S25. XAS reference compounds. **(a)** Cu *K*-edge; Cu_2O, Cu_2O **(b)** Fe *K*-edge; FeO, Fe_2O_3, $K_4[Fe(CN)_6]$, $K_3[Fe(CN)_6]$ **(c)** Zn *K*-edge; $ZnSO_4 \cdot 7H_2O$, ZnO, and metallic Zn foil. **(d)** FT-EXAFS of the Cu references in (a). **(e)** FT-EXAFS of the Fe references in (b). **(f)** FT-EXAFS of the Zn references in (c). Note that the XANES spectrum of the ZnO reference in (c) is fetched from Collins et al.[14] , the FT-EXAFS in (f) is simulated using FEFF [4] using the cubic ZnO phase fetched from the materials project database (mp-2229) as input structure.[15] The other compounds were measured at the KMC-3 beamline at BESSY II (HZB, Berlin).

Table S8. Edge positions of the *K*-edges of reference compounds. The edge positions were extracted at half-height of the normalized edge jump, i.e., at 0.5 absorption units.

Edge	Compound	Spin-state	Oxidation state	Edge position 1s→4p (eV)	Peak position 1s→4p (eV)	Reference
**Fe *K***	$K_4Fe(CN)_6$	Low-spin	Fe^{2+}	7125.6		* This work
**Fe *K***	$K_3[Fe(CN)_6]$	Low-spin	Fe^{3+}	7126.2		* This work
**Fe *K***	FeO	High-spin	Fe^{2+}	7119.1		* This work
**Fe *K***	Fe_2O_3	High-spin	Fe^{3+}	7123.8		* This work
**Cu *K***	Cu_2O		Cu^{+}	8980.6	8982.3	* This work
**Cu *K***	CuO		Cu^{2+}	8985.0	8986.9	* This work
**Zn *K***	$ZnSO_4$		Zn^{2+}	9661.8		* This work
**Zn *K***	ZnO		Zn^{2+}	9661.6		[14]
**Zn *K***	Zn foil		Zn^{0}	9660.7		* This work

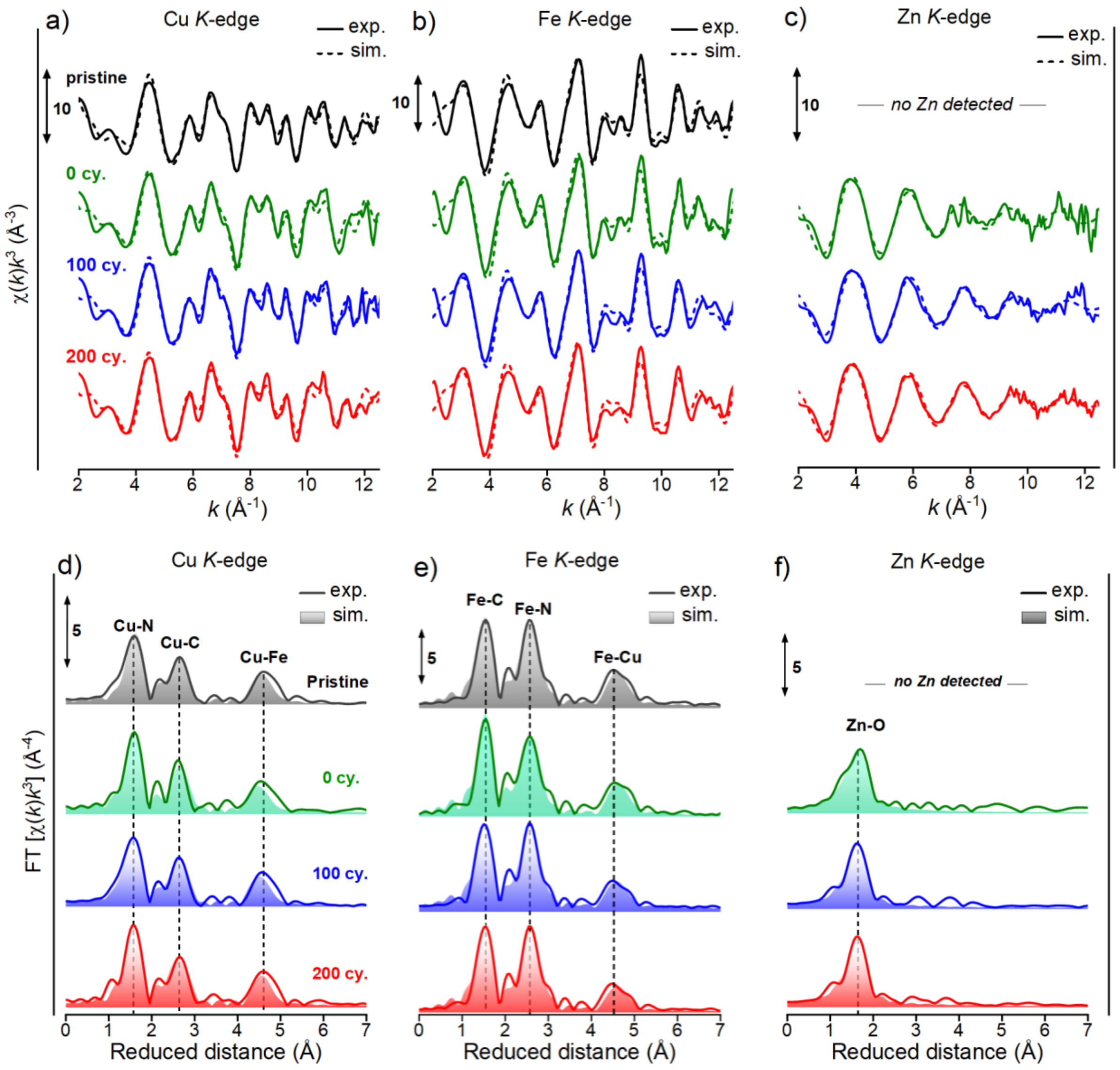

Figure S26. The k^3-weighted $\chi(k)$ EXAFS of the CuHCF cathode after cycling up to 200 cycles between 1.00 V - 2.15 V in 1M $ZnSO_4$. **(a)** at the Cu *K*-edge **(b)** Fe *K*-edge, and **(c)** Zn *K*-edge. The experimental spectra are shown as solid lines and the simulated spectra as dashed lines. The corresponding FT-EXAFS is shown in **(d)** Cu *K*-edge **(e)** Fe *K*-edge **(f)** Zn *K*-edge. The experimental spectra are shown as solid lines, and the simulated spectra as shaded areas under the curves. All samples were stopped at OCP and rinsed with ultrapure water prior to analysis to remove excess of $ZnSO_4$ electrolyte.

Table S9. Fit parameters from the EXAFS simulations of the Cu, Fe and Zn *K*-edges of the CuHCF cathode using scattering functions generated in FEFF.[1] The simulations were performed between a *k*-range of ~2-13 $Å^{-1}$. For the Cu and Fe *K*-edges, the coordination numbers were fixed, while they were minimized for the Zn *K*-edge due to diverse structures. An amplitude reduction factor (S_0^2) of 0.85 was used for all *K*-edges. The samples stopped at OCP were analyzed in "ex situ" configuration (i.e., the cells had been disassembled and the samples rinsed with water before analysis), and the samples analyzed in the charged (2.15 V) or discharged (1.00 V) states were analyzed in "in situ" configuration (i.e., in real-time while mounted in the cells and applying a potential).

**CuHCF cathode (EXAFS simulations)**					
**Cu *K*-edge**	**R_{Cu-N} (Å)** (N = 4)	**R_{Cu-C} (Å)** (N = 4) [(a)]	**R_{Cu-Fe} (Å)** (N = 4)	**R_{Cu-O} (Å)** (N = 2) [(a)]	**N_{MS} (N-C)** (3+4 leg)
Pristine	1.96 ± 0.01	3.13 ± 0.02	4.85 ± 0.07	1.99 ± 0.04	5.1 ± 0.7
0 cycles (OCP)	1.95 ± 0.02	3.09 ± 0.01	4.87 ± 0.06	1.99 ± 0.01	3.8 ± 0.7
100 cycles (OCP)	1.94 ± 0.02	3.10 ± 0.01	4.86 ± 0.07	1.99 ± 0.04	4.5 ± 0.8
200 cycles (OCP)	1.95 ± 0.02	3.10 ± 0.01	4.83 ± 0.10	1.99 ± 0.04	4.4 ± 0.7
2.15 V (CHA)	1.97 ± 0.03	3.12 ± 0.02	4.90 ± 0.06	1.99 ± 0.05	4.2 ± 0.8
1.00 V (DCH)	1.95 ± 0.05	3.08 ± 0.03	4.83 ± 0.10	1.97 ± 0.08	5.0 ± 1.0
**Fe *K*-edge**	**R_{Fe-C} (Å)** (N = 6)	**R_{Fe-N} (Å)** (N = 6)	**R_{Fe-Cu} (Å)** (N = 6)		**N_{MS} (Fe-CN-Cu)** (3+4 leg)
Pristine	1.92 ± 0.01	3.08 ± 0.01	4.82 ± 0.01		10.1 ± 1.4
0 cycles (OCP)	1.92 ± 0.01	3.09 ± 0.01	4.83 ± 0.01		10.4 ± 1.6
100 cycles (OCP)	1.92 ± 0.01	3.08 ± 0.01	4.82 ± 0.01		9.7 ± 1.7
200 cycles (OCP)	1.92 ± 0.01	3.08 ± 0.01	4.83 ± 0.01		9.9 ± 1.8
2.15 V (CHA)	1.93 ± 0.01	3.09 ± 0.01	4.86 ± 0.01		9.2 ± 1.2
1.00 V (DCH)	1.90 ± 0.01	3.08 ± 0.01	4.81 ± 0.01		8.8 ± 1.2
**Zn *K*-edge**	**R_{Zn-O} (Å)**	**N_{Zn-O}**			
pristine	n/a	n/a			
0 cycles (OCP)	2.09 ± 0.01	6.0 ± 0.6			
100 cycles (OCP)	2.06 ± 0.01	5.7 ± 0.5			
200 cycles (OCP)	2.06 ± 0.01	6.1 ± 0.6			
2.15 V (CHA)	2.08 ± 0.02	3.4 ± 0.5			
1.00 V (DCH)	2.09 ± 0.02	3.2 ± 0.5			

R = coordination distance, N = coordination number, N_{MS} is the number of scatters contributing to multiple scattering considering the sum of the 3 and 4-leg paths.

[(a)] The coordination number for the Cu-C and the Cu-O shells were both fixed to a total number of 6, where the Cu-O shell replaces the $Fe(CN)_6$ vacant sites.

Table S10. Debye-Waller parameters, ionization energy correction, and R-factor filtered for the fit parameters presented in Table S9 above. Fit errors were estimated up to a distance of 5 Å useful R-space.

CuHCF cathode (Debye-Waller parameters)						
**Cu *K*-edge**	**σ_{Cu-N} (Å)** (N = 4)	**σ_{Cu-C} (Å)** (N = 4)	**σ_{Cu-Fe} (Å)** (N = 4)	**σ_{Cu-O} (Å)** (N = 2)	**ΔE_0**	**R_f**
pristine	0.07 ± 0.02 [(a)]	0.07 ± 0.02 [(a)]	0.05 ± 0.01	0.07 ± 0.02 [(a)]	2.2	24.1
0 cycles	0.08 ± 0.01 [(a)]	0.08 ± 0.01 [(a)]	0.06 ± 0.01	0.08 ± 0.01 [(a)]	-1.5	32.3
100 cycles	0.10 ± 0.01 [(a)]	0.10 ± 0.01 [(a)]	0.05 ± 0.01	0.10 ± 0.01 [(a)]	-0.3	26.1
200 cycles	0.09 ± 0.01 [(a)]	0.09 ± 0.01 [(a)]	0.06 ± 0.01	0.09 ± 0.01 [(a)]	-0.4	28.9
2.15 V (CHA)	0.10 ± 0.01 [(a)]	0.10 ± 0.01 [(a)]	0.05 ± 0.01	0.10 ± 0.01 [(a)]	-0.4	31.1
1.00 V (DCH)	0.13 ± 0.03 [(a)]	0.13 ± 0.03 [(a)]	0.08 ± 0.01	0.13 ± 0.03 [(a)]	-1.2	35.2
**Fe *K*-edge**	**σ_{Fe-C} (Å)** (N = 6)	**σ_{Fe-N} (Å)** (N = 6)	**σ_{Fe-Cu} (Å)** (N = 6)		**ΔE_0**	**R_f**
pristine	0.07 ± 0.01 [(b)]	0.07 ± 0.01 [(b)]	0.09 ± 0.01		0.7	25.0
0 cycles	0.06 ± 0.01 [(b)]	0.06 ± 0.01 [(b)]	0.10 ± 0.01		1.6	30.0
100 cycles	0.06 ± 0.07 [(b)]	0.06 ± 0.01 [(b)]	0.10 ± 0.01		1.0	27.6
200 cycles	0.07 ± 0.01 [(b)]	0.07 ± 0.01 [(b)]	0.10 ± 0.01		1.0	25.7
2.15 V (CHA)	0.07 ± 0.01 [(b)]	0.07 ± 0.01 [(b)]	0.09 ± 0.01		1.4	26.9
1.00 V (DCH)	0.07 ± 0.01 [(b)]	0.07 ± 0.01 [(b)]	0.09 ± 0.01		0.8	23.6
**Zn *K*-edge**	**σ_{Zn-O} (Å)**				**ΔE_0**	**R_f**
pristine	n/a					
0 cycles	0.10 ± 0.01 [(c)]				2.1	27.9
100 cycles	0.10 ± 0.01 [(c)]				1.1	23.2
200 cycles	0.10 ± 0.01 [(c)]				0.9	20.3
2.15 V (CHA)	0.10 ± 0.01 [(c)]				0.9	32.7
1.00 V (DCH)	0.10 ± 0.01 [(c)]				1.9	28.6

σ = Debye-Waller parameter, ΔE_0 = ionization energy correction, and R-factor filtered (R_f)

(a) The Debye-Waller parameters were minimized in a global fit across the Cu-N, Cu-C, and Cu-O shells, however, were fitted individually for each electrode since the coordination numbers were fixed.

(b) The Debye-Waller parameters were minimized in a global fit across the Fe-N and Fe-C shells, however, were fitted individually for each electrode since the coordination numbers were fixed.

(c) The Debye-Waller parameters were minimized in a global fit across all electrodes since the coordination numbers were not fixed for the Zn edge, to avoid overparameterization.

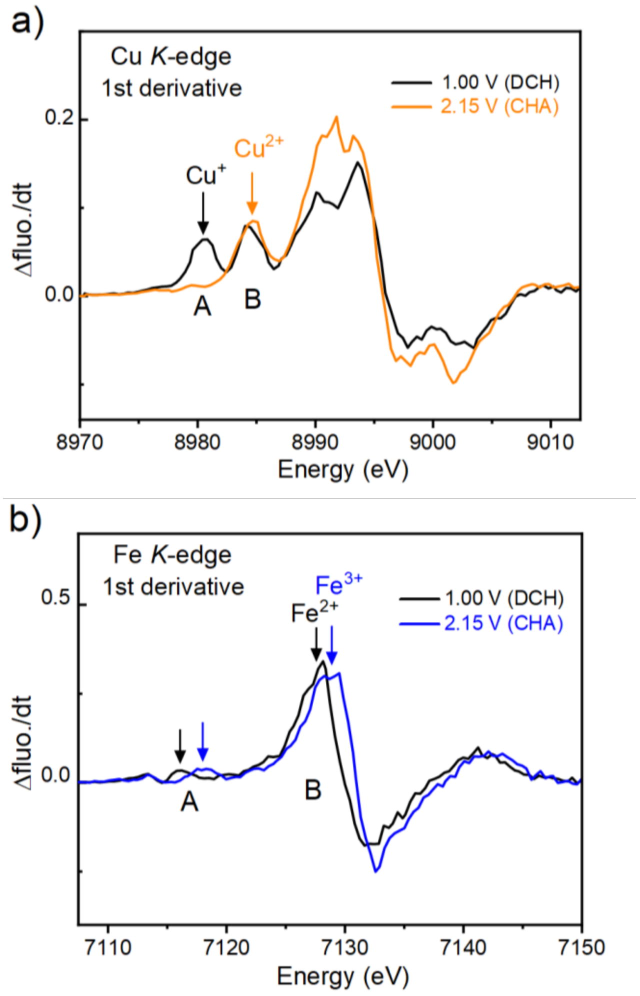

Figure S27. The first derivative of the EXAFS to demonstrate the changes in the XANES region in the discharged state (1.00 V) and in the charged state (2.15 V) of the CuHCF cathode. **(a)** Cu *K*-edge; peak A is the dipole 1s→4p transition of Cu^{+} and peak B of Cu^{2+}. **(b)** Fe *K*-edge; peak A indicates the quadruple 1s→3d transition (pre-edge), and peak B the dipole 1s→4p transition (main edge).

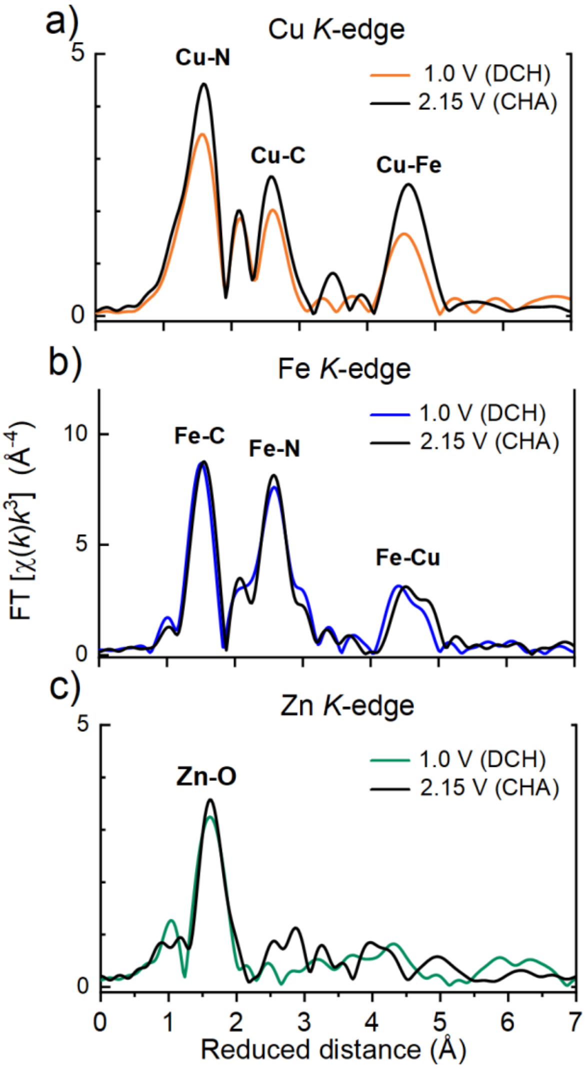

Figure S28. The k^3-weighted FT-EXAFS of the CuHCF cathode in the discharged (1.00 V) and charged (2.15 V) states during in situ conditions of the assembled Zn/CuHCF cell with 1M $ZnSO_4$ electrolyte**. (a)** Cu *K*-edge **(b)** Fe *K*-edge and **(c)** Zn *K*-edge.

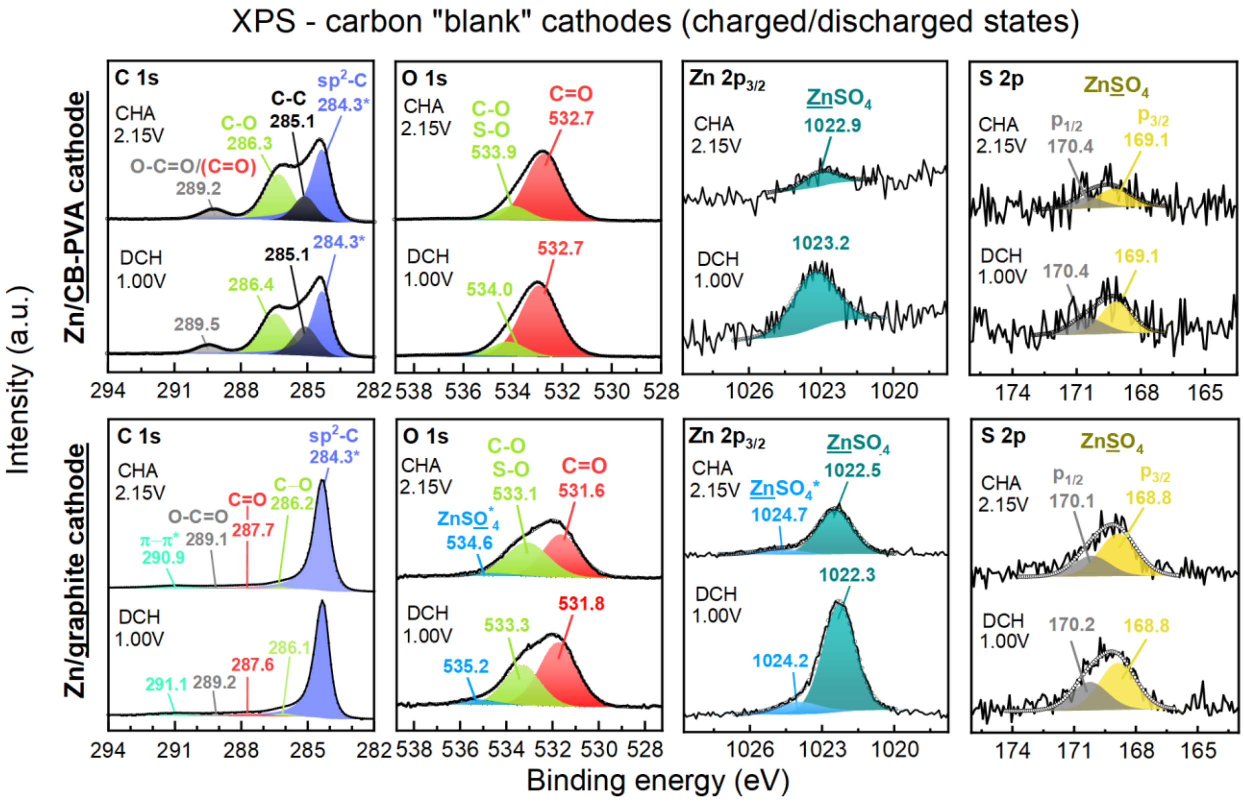

Figure S29. XPS spectra of the cathodes of the two carbon "blank" cells (Zn/CB-PVA and Zn/graphite). The spectra were collected after 200 cycles in 1 M $ZnSO_4$, and then stopped in either the charged (2.15 V) or the discharged (1.00 V) states. The CB-PVA spectra are shown as the top figures, and the graphite cathode as the bottom figures. All electrodes had been rinsed with ultrapure water prior to analysis to remove excess of the $ZnSO_4$ electrolyte; without this step, there were no differences between the charged/discharged states in the Zn 2p and S 2p spectra. All spectra were energy calibrated to the graphitic sp^2-C peak at 284.3 eV.[2,3] The blue component at higher B.E. in the Zn 2p spectrum (<u>Zn</u>SO_4^*) we propose might be related to a surface species of $ZnSO_4$, either crystalline $ZnSO_4$ or a smalller fraction of intercalated $ZnSO_4$ into graphite. The same component in the O 1s spectra at high B.E. may as well be this surface species, or water molecules in a different coordination environment. In the Zn 2p spectrum, it is visible that there is a slightly higher signal from Zn^{2+} (in $ZnSO_4$) in the discharged state (1.00 V) in both the CB-PVA and graphite cathodes, which is interesting since there is no difference between 1.00 V (DCH) and 2.15 V (CHA) for the washed CuHCF cathode shown in Figure 7a in the main manuscript. In these carbon-containing cathodes, there is on the other hand no difference in the SO_4^{2-} signal in the S 2p spectra between 1.00 V and 2.15 V, which is in contrast to CuHCF where there is a significant increase in the SO_4^{2-} signal at 2.15 V.

Table S11. Surface compositions from XPS of the CuHCF cathode and the carbon "blank" cathodes (CB-PVA and graphite) after 200 cycles in 1 M $ZnSO_4$, and stopped either in the charged (2.15 V) or the discharged (1.00 V) states. All samples had been washed with ultrapure water to remove excess of $ZnSO_4$ electrolyte before analysis. The compositions are reported as atomic %.

	**Elemental compositions from XPS (at. %)**						
**200 cycles (washed)**	**Cu 2p**	**Fe 2p**	**N 1s**	**C 1s**	**O 1s**	**Zn 2p**	**S 2p**
CuHCF, 2.15 V	0.50 (0.11)	0.92 (0.12)	14.3 (1.7)	64.7 (0.6)	17.8 (2.1)	1.23 (0.10)	0.95 (0.16)
CuHCF, 1.00 V	0.53 (0.29)	0.74 (0.04)	12.1 (0.4)	66.1 (0.6)	18.6 (0.7)	1.38 (0.44)	0.32 (0.10)
CB-PVA, 2.15 V	n/a	n/a	n/a	77.9 (0.5)	22.0 (0.4)	0.03 (0.02)	0.06 (0.03)
CB-PVA, 1.00 V	n/a	n/a	n/a	77.5 (0.4)	22.4 (0.4)	0.04 (0.02)	0.05 (0.03)
Graphite, 2.15 V	n/a	n/a	n/a	94.5 (0.5)	5.10 (0.50)	0.13 (0.03)	0.32 (0.05)
Graphite, 1.00 V	n/a	n/a	n/a	93.8 (0.2)	5.65 (0.20)	0.24 (0.01)	0.29 (0.05)

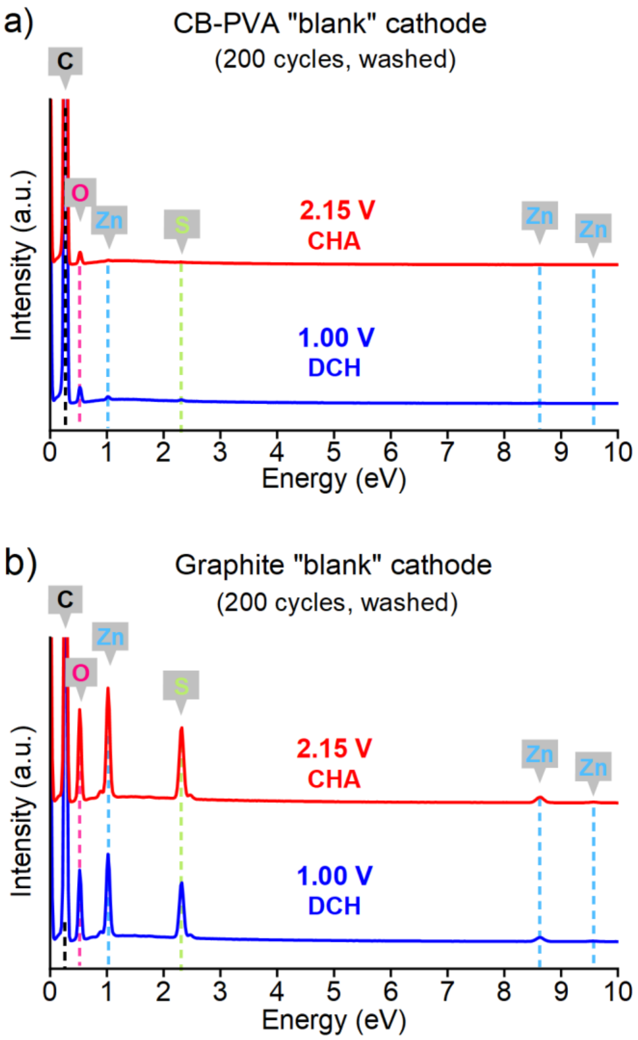

Figure S30. EDS spectra of the two carbon "blank" cathodes after 200 cycles between 1.00 V – 2.15 V at 2.5 mV s^{-1} in 1 M $ZnSO_4$, and stopped in either the charged (2.15 V) or the discharged (1.00 V) states. **(a)** The CB-PVA cathode. **(b)** The graphite cathode. The electrodes had been washed with ultrapure water prior to analysis to remove excess of $ZnSO_4$ electrolyte. All EDS spectra were normalized to the unit area, and both (a) and (b) have the same y-axis scale for comparison reasons.

Table S12. EDS elemental compositions of CuHCF and the two carbon "blank" cathodes (CB-PVA and graphite) after 200 cycles between 1.00 V – 2.15 V, and stopped in either the charged (2.15 V) or the discharged (1.00 V) states. The carbon content has been omitted for comparison reasons. The electrodes were washed with ultrapure water prior to analysis to remove excess of $ZnSO_4$ electrolyte. The standard errors are given in brackets and were estimated from the overall number of measurements of repetitions.

	**Elemental composition from EDS (at. %)**				
**200 cycles (washed)**	**C**	**O**	**Zn**	**S**	**K**
CuHCF, 2.15 V DCH	85.4 (1.8)	8.2 (1.6)	5.0 (0.4)	1.44 (0.08)	n/a
CuHCF, 1.00 V DCH	89.0 (0.8)	6.3 (0.8)	4.3 (0.4)	0.10 (0.04)	0.21 (0.02)
CB-PVA, 2.15 V DCH	97.7 (0.6)	2.2 (0.6)	0.06 (0.01)	0.01 (0.01)	n/a
CB-PVA, 1.00 V DCH	96.9 (0.5)	2.6 (0.2)	0.12 (0.01)	0.05 (0.01)	n/a
Graphite, 2.15 V DCH	80.9 (5.7)	14.4 (4.4)	2.25 (0.55)	2.50 (0.70)	n/a
Graphite, 1.00 V DCH	84.9 (1.1)	11.5 (0.8)	1.80 (0.19)	1.75 (0.19)	n/a

References

(1) Ojwang, D. O.; Grins, J.; Wardecki, D.; Valvo, M.; Renman, V.; Häggström, L.; Ericsson, T.; Gustafsson, T.; Mahmoud, A.; Hermann, R. P.; Svensson G. Structure Characterization and Properties of K-Containing Copper Hexacyanoferrate. *Inorg. Chem.* **2016**, *55* (12), 5924–5934. https://doi.org/10.1021/acs.inorgchem.6b00227.

(2) Moraes, A.; Assumpção, M. H. M. T.; Simões, F. C.; Antonin, V. S.; Lanza, M. R. V; Hammer, P.; Santos, M. C. Surface and Catalytical Effects on Treated Carbon Materials for Hydrogen Peroxide Electrogeneration. *Electrocatalysis* **2016**, *7* (1), 60–69. https://doi.org/10.1007/s12678-015-0279-5.

(3) Blume, R.; Rosenthal, D.; Tessonnier, J.-P.; Li, H.; Knop-Gericke, A.; Schlögl, R. Characterizing Graphitic Carbon with X-Ray Photoelectron Spectroscopy: A Step-by-Step Approach. *ChemCatChem* **2015**, *7* (18), 2871–2881. https://doi.org/https://doi.org/10.1002/cctc.201500344.

(4) Rehr, J. J.; Kas, J. J.; Vila, F. D.; Prange, M. P.; Jorissen, K. Parameter-Free Calculations of X-Ray Spectra with FEFF9. *Phys. Chem. Chem. Phys.* **2010**, *12* (21), 5503–5513. https://doi.org/10.1039/B926434E.

(5) Wardecki, D.; Ojwang, D. O.; Grins, J.; Svensson, G. Neutron Diffraction and EXAFS Studies of K2x/3Cu[Fe(CN)6]2/3·nH2O. *Cryst. Growth Des.* **2017**, *17* (3), 1285–1292. https://doi.org/10.1021/acs.cgd.6b01684.

(6) Mullaliu, A.; Aquilanti, G.; Stievano, L.; Conti, P.; Plaisier, J. R.; Cristol, S.; Giorgetti, M. Beyond the Oxygen Redox Strategy in Designing Cathode Material for Batteries: Dynamics of a Prussian Blue-like Cathode Revealed by Operando X-Ray Diffraction and X-Ray Absorption Fine Structure and by a Theoretical Approach. *J. Phys. Chem. C* **2019**, *123* (14), 8588–8598. https://doi.org/10.1021/acs.jpcc.8b12116.

(7) Giorgetti, M.; Guadagnini, L.; Tonelli, D.; Minicucci, M.; Aquilanti, G. Structural Characterization of Electrodeposited Copper Hexacyanoferrate Films by Using a Spectroscopic Multi-Technique Approach. *Phys. Chem. Chem. Phys.* **2012**, *14* (16), 5527–5537. https://doi.org/10.1039/C2CP24109A.

(8) Renman, V.; Ojwang, D. O.; Valvo, M.; Gómez, C. P.; Gustafsson, T.; Svensson, G. Structural-Electrochemical Relations in the Aqueous Copper Hexacyanoferrate-Zinc System Examined by Synchrotron X-Ray Diffraction. *J. Power Sources* **2017**, *369*, 146–153. https://doi.org/https://doi.org/10.1016/j.jpowsour.2017.09.079.

(9) Åkerblom, I. E.; Ojwang, D. O.; Grins, J.; Svensson, G. A Thermogravimetric Study of Thermal Dehydration of Copper Hexacyanoferrate by Means of Model-Free Kinetic Analysis. *J. Therm. Anal. Calorim.* **2017**, *129* (2), 721–731. https://doi.org/10.1007/s10973-017-6280-x.

(10) Trócoli, R.; La Mantia, F. An Aqueous Zinc-Ion Battery Based on Copper Hexacyanoferrate. *ChemSusChem* **2015**, *8* (3), 481–485. https://doi.org/10.1002/cssc.201403143.

(11) Ren, S.; Duan, X.; Liang, S.; Zhang, M.; Zheng, H. Bifunctional Electrocatalysts for Zn–Air Batteries: Recent Developments and Future Perspectives. *J. Mater. Chem. A* **2020**, *8* (13), 6144–6182. https://doi.org/10.1039/C9TA14231B.

(12) Busch, M.; Halck, N. B.; Kramm, U. I.; Siahrostami, S.; Krtil, P.; Rossmeisl, J. Beyond the Top of the Volcano? – A Unified Approach to Electrocatalytic Oxygen Reduction and Oxygen Evolution. *Nano Energy* **2016**, *29*, 126–135. https://doi.org/https://doi.org/10.1016/j.nanoen.2016.04.011.

(13) Ferrari, A. C. Raman Spectroscopy of Graphene and Graphite: Disorder, Electron–Phonon Coupling, Doping and Nonadiabatic Effects. *Solid State Commun.* **2007**, *143* (1), 47–57. https://doi.org/https://doi.org/10.1016/j.ssc.2007.03.052.

(14) Collins, D.; Luxton, T.; Kumar, N.; Shah, S.; Walker, V. K.; Shah, V. Assessing the Impact of Copper and Zinc Oxide Nanoparticles on Soil: A Field Study. *PLoS One* **2012**, *7* (8), e42663. https://doi.org/10.1371/journal.pone.0042663.

(15) Jain, A.; Ong, S. P.; Hautier, G.; Chen, W.; Richards, W. D.; Dacek, S.; Cholia, S.; Gunter, D.; Skinner, D.; Ceder, G.; Persson K. A. Commentary: The Materials Project: A Materials Genome Approach to Accelerating Materials Innovation. *APL Mater.* **2013**, *1* (1), 11002. https://doi.org/10.1063/1.4812323.
